# A phage-displayed disulfide constrained peptide discovery platform yields novel human plasma protein binders

**DOI:** 10.1371/journal.pone.0299804

**Published:** 2024-03-28

**Authors:** Xinxin Gao, Harini Kaluarachchi, Yingnan Zhang, Sunhee Hwang, Rami N. Hannoush

**Affiliations:** 1 Department of Early Discovery Biochemistry, Genentech, South San Francisco, California, United States of America; 2 Department of Peptide Therapeutics, Genentech, South San Francisco, California, United States of America; 3 Department of Biological Chemistry, Genentech, South San Francisco, California, United States of America; University of Nova Gorica, SLOVENIA

## Abstract

Disulfide constrained peptides (DCPs) show great potential as templates for drug discovery. They are characterized by conserved cysteine residues that form intramolecular disulfide bonds. Taking advantage of phage display technology, we designed and generated twenty-six DCP phage libraries with enriched molecular diversity to enable the discovery of ligands against disease-causing proteins of interest. The libraries were designed based on five DCP scaffolds, namely *Momordica charantia* 1 (Mch1), gurmarin, Asteropsin-A, antimicrobial peptide-1 (AMP-1), and potato carboxypeptidase inhibitor (CPI). We also report optimized workflows for screening and producing synthetic and recombinant DCPs. Examples of novel DCP binders identified against various protein targets are presented, including human IgG Fc, serum albumin, vascular endothelial growth factor-A (VEGF-A) and platelet-derived growth factor (PDGF). We identified DCPs against human IgG Fc and serum albumin with sub-micromolar affinity from primary panning campaigns, providing alternative tools for potential half-life extension of peptides and small protein therapeutics. Overall, the molecular diversity of the DCP scaffolds included in the designed libraries, coupled with their distinct biochemical and biophysical properties, enables efficient and robust identification of *de novo* binders to drug targets of therapeutic relevance.

## Introduction

Since the development of insulin therapy in the 1920s, peptides have been pursued for a variety of applications in both biotechnology and therapeutics. To date, more than 80 peptide therapeutics have been approved to treat a wide range of diseases, including cancer, osteoporosis, diabetes, multiple sclerosis and chronic pain [1, 2]. Peptides represent a distinctive class of pharmaceutical entities with their size fitting between small molecules and proteins, and they present unique biochemical, biophysical and pharmaceutical properties. The effectiveness of a given drug is also highly dependent on its intrinsic pharmacokinetics. Due to their small size, peptides and small proteins are usually rapidly eliminated from circulation as a result of metabolism and renal filtration [3]. To overcome this shortcoming, strategies have been developed to extend their *in vivo* half-life, including engineering peptides to interact with human plasma proteins (human IgG Fc or serum albumin), to delay renal clearance and intracellular catabolism [4]. For example, the long-acting insulin molecule detemir (Levemir®) was developed through fatty acylation of the ɛ-amino group of lysine B29 (which was designed to replace Thr-30 in native insulin) [5, 6]. This insulin analogue demonstrated a prolonged duration of action *in vivo*, most likely via binding to human serum albumin [7]. Recent identification of novel plasma protein binding peptides has greatly expended the toolbox of half-life extension for peptides and small proteins [8–10].

Cystine-knot peptides (CKPs), a subgroup of disulfide constrained peptides (DCP), usually contain 30–40 amino acid residues with conserved cysteines forming intramolecular disulfide bonds arranged in a knotted conformation (Cys I-Cys IV, Cys II-Cys V, Cys III-Cys VI connectivity). CKPs have gained much interest as attractive templates for drug discovery due to their hypervariable loops between the disulfide bonds, as well as remarkable chemical, thermal, and proteolytic stability owing to their constrained structures [11–17]. Naturally occurring DCPs/CKPs demonstrate a wide range of pharmacological activities [14, 18]. For example, two DCP-based drugs, Ziconotide [19, 20] (for treatment of severe chronic pain) and Linaclotide [21] (for treatment of constipation-predominant irritable bowel syndrome/chronic idiopathic constipation) have been developed utilizing their native pharmacologic activities. The high diversity in amino acid composition present in the loop regions flanking the conserved cysteines, which are responsible for their pharmacological activities, indicates that novel bioactive features could be developed through modification of the native sequences in these regions. This strategy has been adapted to engineering DCP variants to target kinases, proteases, GPCRs, growth factors, and protein-protein interactions through substituting the loop regions with known linear bioactive epitopes [22–28]. However, this strategy does not offer an effective way to systematically replace the amino acid composition in each loop in a high throughput fashion, therefore suffers from low efficiency and high failure rates. To circumvent this issue, a recent study demonstrated the use of mRNA display to discover potent FXIIa inhibitors from a library based on the DCP scaffold *Momordica cochinchinensis* trypsin inhibitor-II (MCoTI-II) [29]. Development of more *de novo* DCP libraries with high diversity will greatly expand the utility of DCPs in drug discovery.

Phage display [30–32], a technique involving the display of peptide libraries on the surfaces of bacteriophage (as fusions to the major (pVIII) or minor (pIII) bacteriophage coat protein), is a robust tool for high-throughput screening of different target-specific ligands. The technique allows for the construction of completely randomized peptide libraries which can be decoded by DNA sequencing. Due to its time-efficient and effective features, phage display based library selection has become one of the most prevalent screening technologies in both basic research and therapeutics [33]. To meet the demand, peptide phage libraries have been developed and utilized to identify target binding peptides [34]. The DCP family offers an attractive opportunity for phage library construction with high diversity. Taking advantage of the loop regions of these peptides, we have previously engineered phage libraries using eight DCP scaffolds as templates, including EETI-II (loop 1, 5, or 1/5 randomized), AVR9 (loop 4 randomized), Circulin-A (loop 4 randomized), conotoxin-MVIIA (loop 2 randomized), Huwentoxin (loop 2 randomized), Charybdotoxin (loop 1/2/3, loop 4, or C-term + loop 3/4/5 randomized), cellulose binding protein (CBD) (loop 1/3 or N-term + loop 3 randomized), and CBD amylose (loop 1/3 or N-term + loop 3 randomized) [35, 36]. Six out of the eight DCPs are CKPs, while CBD and CBD amylose, containing only four cysteines, do not form knotted conformation. These DCP phage libraries were used to identify binders against different protein targets successfully [35, 36]. Here, we expanded the molecular diversity of the DCP phage libraries through engineering additional 26 new DCP phage libraries based on five different DCP scaffolds, namely *Momordica charantia* 1 (Mch1) [37], gurmarin [38], Asteropsin-A [39], antimicrobial peptide-1 (AMP-1) [40], and potato carboxypeptidase inhibitor (CPI) [41]. Together with previously developed DCP phage libraries, the new set of libraries provide an efficient and robust approach to identify binders from multiple DCP scaffolds that possess distinct biochemical and biophysical properties. Furthermore, to facilitate DCP production, we optimized methods to produce synthetic or recombinant DCP binders. Using this new platform, we sought to discover peptides binding to human plasma protein to extend the half-life of peptides and small proteins. In this study, we have discovered a series of DCP variants that bind to human IgG Fc or human serum albumin with sub-micromolar affinity, as well as binders against human vascular endothelial growth factor-A (VEGF-A) and platelet-derived growth factor (PDGF). The human plasma protein binding DCPs demonstrate great specificity and good stability in human serum. The identification of these DCP binders showcases the versatility of the platform, and offers alternative solutions for potential half-life extension of peptides and small protein therapeutics.

## Materials and methods

### DCP phage library design and construction

The DCP phage libraries were generated as previously described [35, 36]. Five naturally occurring DCPs (Mch1, gurmarin, Asteropsin-A, AMP-1 and CPI) were selected as scaffolds for display on the surface of M13 bacteriophage. Each DCP was fused via the C-terminus to the N-terminus of M13 major coat protein (pVIII), with a gD (HSV-2 envelope glycoprotein D)-tag fused to the N-terminus of the DCP sequence. To prevent introduction of extra Cys within the loop region, libraries were generated with trinucleotide oligos where residues denoted with “X” were replaced with equimolar of 19 amino acids (Cys excluded) [36]. The extended DCP phage libraries contain 13 DCP frameworks as shown in S1 Table of S2 File.

### Design and construction of the fusion DCP protein expression plasmids

For constructing the fusion DCP protein expression plasmids, a modified pST239 vector with a STII secretion signal for periplasmic targeting was utilized (Genentech). The parental plasmid which was used for inserting the DCP of interest has an N-terminal His(6) and a soluble fusion tag corresponding to either MBP, SUMO or Ubiquitin followed by a TEV protease cutting site. Primers encoding the DCPs (Integrated DNA Technologies, Inc.) with codons optimized for *E*. *coli* expression were then sub-cloned into the vector such that the TEV recognition sequence (ENLYFQ/G) was followed immediately by the DCP sequence.

### Recombinant expression and purification of the fusion DCP proteins

Plasmids were transformed into 44H9 strain (developed in Genentech, used to generate recombinant proteins with disulfide bonds) [15, 17] and 4 mL overnight cultures were grown at 30°C in LB media with 50 μg/mL carbenicillin in 24-well UNIPLATE round bottom blocks (E&K Scientific). For overexpression, 4 mL cultures of soy C.R.A.P. phosphate limiting media (3.57 g (NH_4_)_2_SO_4_, 0.71 g Na citrate–2xH_2_O, 1.07 g KCl, 5.36 g Yeast Extract (certified), 5.36 g HycaseSF-Sheffield, pH adjusted with KOH to 7.3, volume adjusted to 872 mL with deionized water and supplemented with 110 mL 1 M MOPS, pH 7.3, 11 mL 50% glucose, 7 mL 1 M MgSO_4_) with 50 μg/mL carbenicillin was inoculated with 80 μL of the overnight culture and grown for additional 24 h at 30°C. Cells were then harvested by centrifugation at 3000 rpm for 0.5 h and stored as pellets at -80°C until purification. For purification of the fusion protein, the cell pellets were thawed on ice in the presence of 1 mL of chilled lysis buffer consisting of 50 mM Tris pH 8.0, 300 mM NaCl, 5 mM Imidazole, 5 mM MgCl_2_, 2 μL/mL Lyzonase (EMD Millipore), 1 tablet of cOmplete™, EDTA-free protease inhibitor cocktail (Roche) /50 mL of buffer. Resuspended cells where then transferred to a 48-well pyramid bottom storage plate (E&K Scientific) and sonicated for a total of 3 min with 1 min pulses. The soluble lysate fraction was isolated by centrifugation at 5200 rpms for 0.5 h. The resulting supernatant was then divided in half and filtered by passing through two UNIFILTER 96-well microplates (GE Healthcare). His(6) purification of the protein was automated using the Oasis liquid handler (Dynamic Devices, Inc.) and Ni-IMAC tips (PhyNexus Inc). Purified protein was then quantitated using Nanodrop and analyzed with SDS-PAGE.

### Folding analysis of recombinant DCPs by LC/MS

TEV protease was used to separate the DCPs from the protein tags. EDTA (0.5 mM) and TEV protease (0.025 mg/mL) were added to 10 μL of the purified fusion proteins from above and the reaction was left at room temperature overnight. The products were then analyzed using an analytical LC-MS (Agilent 1260 Infinity) equipped with a PLRP-S reversed phase column (Agilent, PL1912-1802) with a flow rate of 0.5 mL/min and linear gradient of 15–60% acetonitrile with 0.05% TFA over 6 min.

### Generation of synthetic DCPs

Linear peptides were synthesized and purified as described previously [35, 42, 43]. They were synthesized on a CS Bio peptide synthesizer (CS136M) with standard Fmoc synthesis on 2-chlorotrityl resin and cleaved from resin with TFA:EDT:TIPS:H_2_O 94:2:2:2. Linear peptides were then purified with a C4 or C18 reversed phase HPLC column. Small scale folding analysis was performed using various folding buffer for three days at room temperature (S2 Table in S2 File). The folding reactions from day 1, 2, and 3 were monitored by LC-MS to analyze folding efficiency. Typically, one major peak containing the three disulfide bonds corresponding to folded DCP was observed from the UV trace after 1 or 2 days. Large quantity of linear DCPs was then folded in the optimal folding buffer (for this study, most DCPs were folded at 0.5 mg/mL concentration in 0.1 M ammonium bicarbonate, pH 9.0, 2 mM reduced glutathione, 0.5 mM oxidized glutathione, 4% DMSO; or 0.1 M ammonium bicarbonate, pH 8.0, 1 mM reduced glutathione, 50% DMSO for 24 h at room temperature with shaking). Excess salt was removed by C18 Sep-Pak (Waters, cat # WAT043345) and the folded DCPs were lyophilized, reconstituted in DMSO:H_2_O (1:1) and then purified with a C18 reversed phase HPLC column. The final products were confirmed by LC-MS (the same analysis method as recombinant DCPs). Peptide content was calculated using amino acid analysis performed by the Proteomics Core facility at UC Davis (Davis, CA) or CS Bio (Milpitas, CA). Select peptides were synthesized by CS Bio (Milpitas, CA).

### Protein biotinylation

100 μL of 50 μM of protein of interest was incubated with 100 μM of EZ-link-NHS-PEG4-biotin (Thermo Fischer Scientific) for 2 h at room temperature. The reaction was quenched with 10 mM Tris pH 8.0, followed by dialysis into PBS overnight at 4°C. The final biotinylated protein was analyzed using LC-MS. Typically, each protein was labeled with 1–2 biotin molecules.

### Panning using the DCP phage libraries

The DCP phage libraries were pooled into three major groups as shown in S2 Table of S2 File: Lib 1, Lib 2, and Lib E. The libraries were subjected to four rounds of selection in solution on biotinylated human IgG Fc (Jackson Immunoresearch Labs Inc. cat# 009060008, Biotin-SP-ChromPure Human IgG, Fc fragment), biotinylated human serum albumin (Rockland Immunochemicals, Inc. cat# 009–0633), biotin-PDGF-bb (R&D Systems, cat# BT220-010/CF), biotin-VEGF-A (8–109, Genentech), or biotin-Ly6E-rat IgG2b Fc (Genentech) as described previously [35] with modification. Briefly, for the first round, 20 μg of biotinylated protein was incubated with the phage libraries for 2 h at 4°C, the mixture was incubated with 100 μL pre-blocked (with PBS containing 0.5% BSA, 2 h at room temperature) streptavidin beads (Dynabeads M-280 Streptavidin, ThermoFisher Scientific) for 15 min at room temperature to capture the phage bound to the biotinylated protein. The beads were then washed with PBS containing 0.05% tween 20 and the bound phages were subsequently eluted using 400 μL of 100 mM HCl and neutralized by adding 60 μL of 1 M Tris pH 11. The phage was amplified and purified for the next round. For round two, 10 μg biotinylated protein was used; for round three, 5 μg biotinylated protein was used and the protein-phage mixture was captured with neutravidin coated on 96-well plates; for round four, 2.5 μg biotinylated protein was used and the protein-phage mixture was captured with streptavidin coated on 96-well plates. To calculate the enrichment levels at rounds 3 and 4, 10 X serial diluted phage (eluted from panning against the target protein or BSA) was plated onto LB/carb plates and cultured overnight at 37°C. The enrichment fold was calculated by dividing the colony numbers from target protein panning by those from negative control (BSA only). For panning against human serum albumin, ovalbumin was used instead of BSA. After the 4^th^ round of panning where enrichment was typically observed, clones binding to target were analyzed by phage spot ELISA and the sequences of these clones were verified by Sanger sequencing. Eluted phage from third and fourth rounds was subjected to NGS using MiSeq (Illumina).

### Phage competition ELISA

To analyze the competitive binding to the target protein for the synthetic DCP and its phage counterpart displaying the same DCP, 384-well plates were coated with 2 μg/mL of neutravidin in PBS overnight at 4°C. The plates were blocked with PBS containing 0.5% BSA and incubated with biotinylated human IgG Fc or serum albumin for 20 min at room temperature. Serial diluted synthetic DCP was pre-incubated with phage for 30 min at room temperature then the mixture was added to the 384 well plates for 30 min at room temperature. The plates were washed and incubate with anti-M13 antibody (1:10,000, HRP conjugated, Creative Diagnostics, cat# CAB-655M) for 30 min at room temperature, followed by washing, development and quenching (1 M H3PO4). The absorbance at 450 nm was recorded to calculate the IC_50_ values.

### Surface Plasmon Resonance (SPR)

SPR measurements were carried out on a Biacore S200 instrument (GE Healthcare) with PBS-T (PBS with 0.05% Tween 20, pH 7.4) as the assay buffer as described previously [35, 42]. For kinetic measurements, biotinylated protein was captured on three separate sensor channels of a Series S SA Sensor chip (GE Healthcare). The chip was then blocked with 0.1 mg/mL free biotin. A concentration series of the DCP were injected to the chip. Data were double-referenced by subtracting the signal from a control sensor channel (coated with biotin only) and the signal from a buffer injection. Binding data were fitted using the manufacturer’s software (1:1 or two state binding model for single-cycle kinetics or steady-state model for multi-cycle kinetics).

### DCP serum stability assay

The DCPs were incubated in human serum (Sigma, cat# H1388) at a concentration of 0.2 mM (~ 0.7 mg/mL) at 37°C for 24 h. All samples (time 0 and 24 h) were flash frozen in liquid nitrogen at—20°C until sample preparation. To extract DCPs, 50 μL of sample was added to 100 μL of ice cold acetonitrile with 0.1% formic acid and mixed thoroughly with a Vortex mixer. The samples were then centrifuged at 14000 × rpm at 4°C for 30 minutes. 80 μL of the supernatant from each sample was diluted into 80 μL of water containing 0.1% formic acid and analyzed using LC-MS. From the HPLC UV trace (at 220 nm), the DCP peak area of the 24 h incubation sample was integrated and normalized to the DCP peak area of the 0 h incubation sample (set as 100%) [44].

## Results

### Design and generation of additional DCP phage libraries

**Fig 1:**
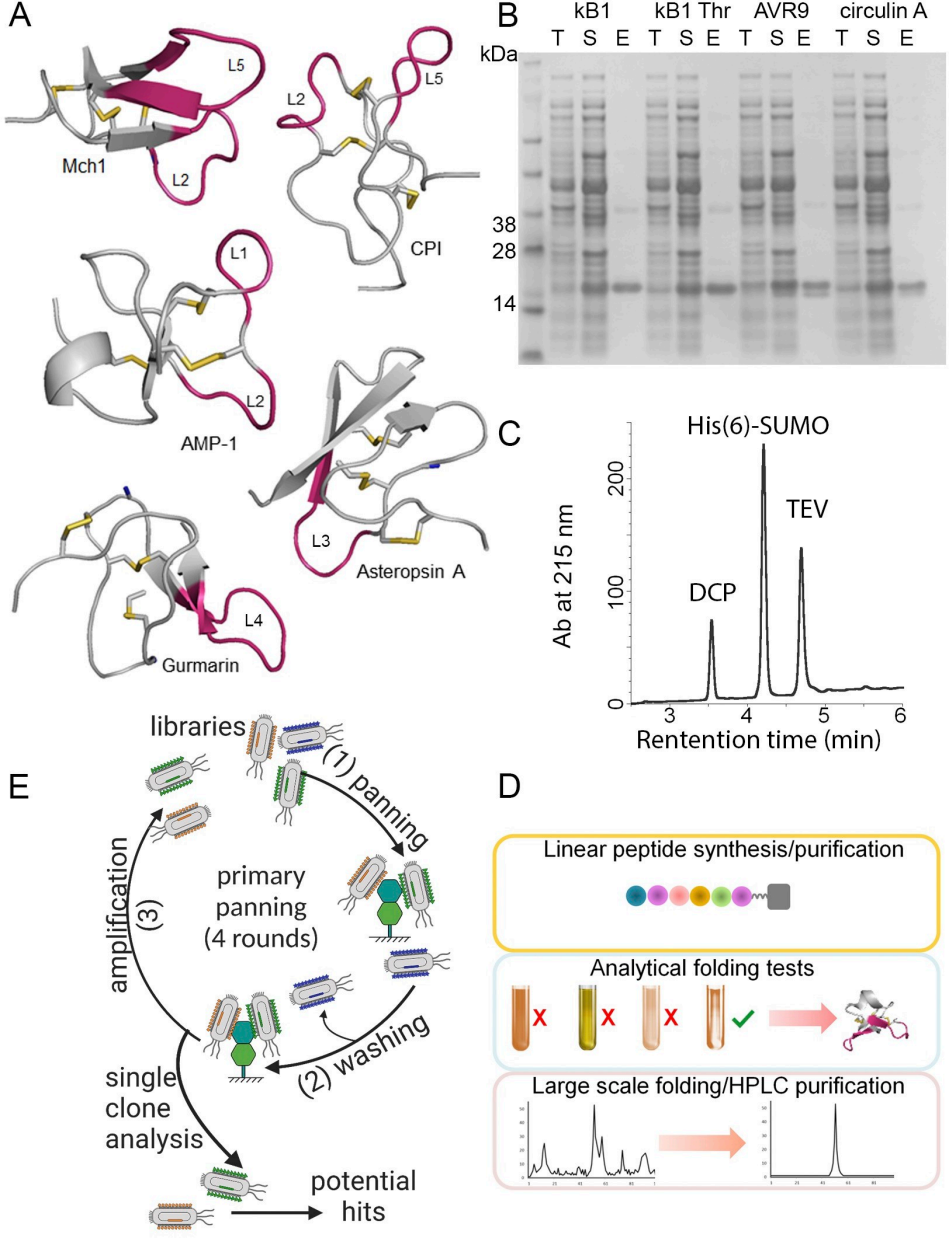
DCP phage library design and production. (A) Structures of wild-type DCPs utilized for constructing the DCP phage libraries. The residues and loops (L) highlighted in pink were subjected to hard randomization or extended to create libraries. Structures were derived from the following PDB files: Mch1 (2M2Q), gurmarin (1C4E), Asteropsin (2LQA), AMP-1 (1MMC), and CPI (4CPA). (B) Expression analysis of four wild-type DCPs with SUMO tag (kB1: kalata B1, MW: 15908.7 kDa; kB1 thr: kalata B1 thrombin binder, MW: 15913.8 kDa; AVR9, MW: 16629.5 kDa; circulin A, MW: 16225.2 kDa). Bacterial expression vectors were generated containing open reading frame with His(6) tag, SUMO, and a TEV protease cutting site at the N-termini of DCPs. The expression profile of the fusion proteins was analyzed with SDS-PAGE. T: total lysate; S: soluble fraction; E: elute. (C) Folding analysis of recombinant wild-type cellulose binding domain (CBD). After TEV protease digestion, the folding profile of CBD was analyzed with LC-MS to show success removal of the SUMO-His(6) tag and confirms the identity of fully oxidized CBD. (D) DCP production using chemical synthesis and thermodynamic oxidation. After linear peptide synthesis and purification, small scale folding analysis was performed using various folding conditions to determine the optimal folding condition yielding a major product containing the three disulfide bonds. The folding product then was desalted using a C18 column, lyophilized and purified through HPLC. The final product was lyophilized and analyzed with LC-MS. (E) Workflow of DCP phage panning. DCP phage libraries were used to select against specific protein targets, followed by washing and elution of the phage displaying DCPs binding to the target. The selection process was repeated four times to improve binder affinity as well as specificity. Individual phage containing DCP binders was isolated and ranked using phage spot ELISA, Sanger sequencing and NGS. The cartoon was created with BioRender.com.

To improve the diversity of the DCP phage libraries, we chose five wild-type DCP scaffolds (Mch-1, gurmarin, Asteropsin-A, AMP-1, and CPI) and randomized the amino acid sequence in their hypervariable loop regions to generate 26 new DCP phage libraries (Fig 1A and S1 Table in S2 File). These five DCPs come from phylogenetically diverse sources and display a wide range of pharmacological activities. Mch-1 [37], gurmarin (rat/mouse sweet-taste receptor inhibitor) [38], CPI (carboxypeptidase inhibitor) [41], and AMP-1 (antimicrobial peptide) [40] were isolated from plants (*Momordica charantia*, *Gymnema sylvestre*, potato, and *Amaranthus caudatus*, respectively), while Asteropsin-A (neuronal Ca^2+^ influx enhancer) [39] was identified from marine sponge. They demonstrate remarkable enzymatic and thermal stability [45, 46], and are amenable to sequence changes [47]. Select loops were randomized alone or in combination with other loops to maximize the diversity and therefore increase the probability of identifying diverse binders. For the Mch1 libraries, we randomized loop 2 (retained its original length, 7 residues), loop 5 (varied from 7 to 11 residues), or both loop 2 and 5 together, to generate seven libraries. For the gurmarin libraries, we deleted the N terminal pyroglutamic acid and randomized loop 4 (varied from 7 to 11 residues) to generate three libraries. Two Asteropsin-A libraries were generated with loop 3 randomized (varied from 6 to 8 residues). And seven AMP-1 libraries were constructed with randomized loop 1 (varied from 4 to 8 residues), loop 2 (retained its original length, 4 residues), or loop 1 and 2 together. Lastly, we generated seven CPI libraries consisting of randomized loop 2 (retained its original length, 5 residues), loop 5 (varied from 6 to 10 residues), or both loop 2 and 5 together, all with the first two amino acids (QQ) deleted (S1 Table in S2 File).

We constructed these libraries by fusing the N-terminal gD-tagged DCPs to the N terminus of the M13 major coat protein pVIII. The phage libraries were quality-controlled by next-generation sequencing (NGS) analysis before proceeding to the selection step. Combined with the previously engineered libraries [35, 36], we have developed in total fifty-nine individual DCP phage libraries based on thirteen DCP scaffolds (S1 Table in S2 File). To streamline the screening process, these DCP phage libraries were pooled into three groups (Lib E, containing EETI-II libraries; Lib 1, containing AVR9, Circulin-A, conotoxin-MVIIA, Huwentoxin, Charybdotoxin, CBD, CBD amylose libraries; and Lib 2, containing Mch1, gurmarin, Asteropsin-A, AMP-1, CPI libraries) for phage panning (S1 Table in S2 File).

### Development of a DCP discovery platform through optimized recombinant and synthetic DCP production methods

To develop an efficient DCP discovery platform, we sought to optimize the synthetic and recombinant DCP production methods. First, we selected several prototypical cysteine constrained scaffolds to test the efficiency of recombinant production of DCPs. Some of these scaffolds, such as AVR9, Circulin-A, conotoxin-MVIIA, Huwentoxin, Charybdotoxin and CBD have been included in the design of the original DCP phage libraries (Lib 1 in S1 Table of S2 File). We also included the cyclotide kalata B1 [13, 48, 49] and a thrombin inhibitor derived from it, kalata thrombin [50], as open-chain DCPs with a break in loop 2 (GTCNTPGCTCSWPVCTRNGLPVCGETCVG for kalata B1 and GTCNTPGCTCSWPVCIDGGRLMCGETCVG for kalata thrombin). A few bacterial expression systems have been successfully applied for expressing a number of disulfide rich proteins or naturally existing DCPs [51, 52]. To develop a generalizable method for high throughput DCP production, we first tested the commercially available pET vector system for expressing these wild-type DCPs in *E*.*coli* cytoplasm or in the oxidizing environment of the periplasm. Expression in the periplasmic environment was evaluated by fusing the wild-type DCP construct to the maltose-binding protein (MBP), which contained a MalE secretion signal for targeting. Of the nine DCPs we tested, three DCPs (conotoxin-MVIIA, Huwentoxin, and Charybdotoxin) could not be recovered after TEV protease digestion; whereas two DCPs (kalata B1 and Min23) were isolated as a mixture consisting of fully oxidized and partially oxidized peptides (S1, S2 Figs in S1 File). The remaining peptides (kalata thrombin, AVR9, Circulin-A, and CBD) were isolated as fully oxidized isomers with yields of ~10 mg/L (S1 Fig in S1 File). In a parallel effort, the feasibility of expressing DCPs in the reducing cytoplasm of bacteria was also assessed by replacing MBP with the disulfide isomerase, DsbC (S1 Fig in S1 File). The DsbC protein has been successfully used as a fusion tag for isolation of several disulfide rich proteins from *E*.*coli* cytoplasm, where the best yields were reported from the BL21(DE3) pLysS strain [53]. Thus, the expression of three different DCPs (kalata B1, Min23, CBD) was tested as fusions to DsbC in the pLysS strain. All three peptides were successfully purified from the soluble fraction with high yields (>10 mg/L) as fusions to DsbC (S1 Fig in S1 File). However, analysis of the purified material via LC-MS after cleavage with TEV protease indicated that every peptide contained a modification corresponding to an addition of 163–165 Da (S2 Fig in S1 File). Our attempts to identify the modification via peptide digestion followed by mass spectrometry methods were unsuccessful.

Given the partial success of isolating fully oxidized DCPs when targeted to the periplasm and the observed modification when expressed in the cytoplasm, we next explored utilizing a pST239 vector containing a heat-stable enterotoxin II (STII) secretion signal for protein expression. This particular vector backbone has been previously used for purifying functional full-length antibodies [54] as well as a large number of bispecific antibodies from *E*. *coli* periplasm [55]. We rationalized that the STII secretion signal which exports proteins co-translationally would result in higher success rate of obtaining fully oxidized DCPs by limiting exposure to the cytoplasmic environment compared to the post-translational secretion by MalE signal [56]. In addition, the expression of this plasmid can be conducted in a W3110 derivative strain which would enable the use of existing 10-liter bioreactor conditions for producing these fusion peptides in large scale if needed. Three different soluble fusion tags (MBP, SUMO and ubiquitin) were tested against nine different wild-type DCPs (kalata B1, kalata thrombin, AVR9, Circulin-A, conotoxin-MVIIA, Huwentoxin, MIN23, Charybdotoxin and CBD). As illustrated in S3-S5 Figs of S1 File, we were able to obtain fully oxidized DCP using any of the three tags (except MBP tagged AVR9), with MBP- and SUMO-tagged fusion proteins showing the highest yields. For the ubiquitin-tagged fusion protein expression, a truncation product with 9445 Da mass was observed routinely that varied in abundance (S5 Fig in S1 File). Since the size of the SUMO tag is smaller than MBP, which resulted in higher yields of DCP itself, we selected His(6)-SUMO-TEV-DCP as the construct to be utilized in the high throughput triaging platform. We chose TEV protease to remove the His(6)-SUMO due to its high efficiency and specificity. The His(6)-SUMO-TEV-DCP fusion proteins could be recombinantly overexpressed and purified using an automated liquid handler (Oasis) with Ni-IMAC tips (PhyNexus Inc). The expression profile of the fusion proteins was then analyzed with SDS-PAGE (Fig 1B and S4 Fig in S1 File). After TEV protease digestion, the folding (disulfide bond formation) of the DCPs was assessed by LC-MS (Fig 1C and S5 Fig in S1 File).

While recombinant expression methods proved successful, synthetic production of DCPs is traditionally used for large scale production and incorporation of chemical modifications. Previous studies have shown that DCPs could be produced synthetically by either folding the linear peptide in a redox buffer where the disulfides shuffle until they reach the most thermodynamically favorable conformation [57], or by orthogonal protection of the cysteine side chain during solid phase peptide synthesis followed by selective deprotection and disulfide bond formation one at a time [58]. We reasoned that the thermodynamic folding method is more time- and cost-efficient, and developed a generalizable approach for DCP production by testing different folding conditions of the linear peptides. Chemical folding conditions for DCPs were optimized with different buffer systems, organic solvents, and redox reagents (S2 Table in S2 File). We identified two optimal conditions (S2 Table in S2 File) under which majority of the linear peptides could be folded into one major folded DCP product with three disulfide bonds. The folded products were then analyzed using LC-MS (S6 Fig in S1 File). The optimal DCP folding conditions were then applied to large scale chemical production and purification (through desalting and HPLC) of DCPs (Fig 1D and S6 Fig in S1 File). The high throughput expression system developed here provides a robust and efficient method to generate a large number of DCPs in small quantities readily available for screening, while the chemical synthesis/folding method is suitable for large scale production (gram level) of DCPs, and addition of chemical moieties (such as unnatural amino acids and drug payloads) to DCPs.

We subsequently streamlined the DCP phage panning process for binder identification, production and validation. The DCP phage libraries were used to select against specific protein targets, followed by washing (to get rid of the non-specific binders) and elution of the phage displaying DCPs binding to the target (Fig 1E). The selection process was repeated four times to improve binder affinity as well as specificity. Individual phage containing DCP binders were isolated and ranked using phage spot ELISA, Sanger sequencing and NGS (Fig 1E and S7 Fig in S1 File). To produce DCP binders, firstly, small quantities of recombinant DCPs were obtained using the high throughput expression/purification system to test the folding of the DCP variants, then the top DCP candidates (selected based on the ELISA, NGS and expression data) were chemically synthesized/folded to produce larger quantities of DCPs for biochemical, biophysical and functional characterization (S7 Fig in S1 File). The best binders from this round of screening could be used as templates for further affinity maturation to achieve desired affinity [35]. This workflow described herein is highly efficient and provides a robust platform for developing DCP agonists/antagonists.

### Identification and production of DCP binders to human IgG Fc (Ig-DCP) and human serum albumin (Hs-DCP)

**Fig 2:**
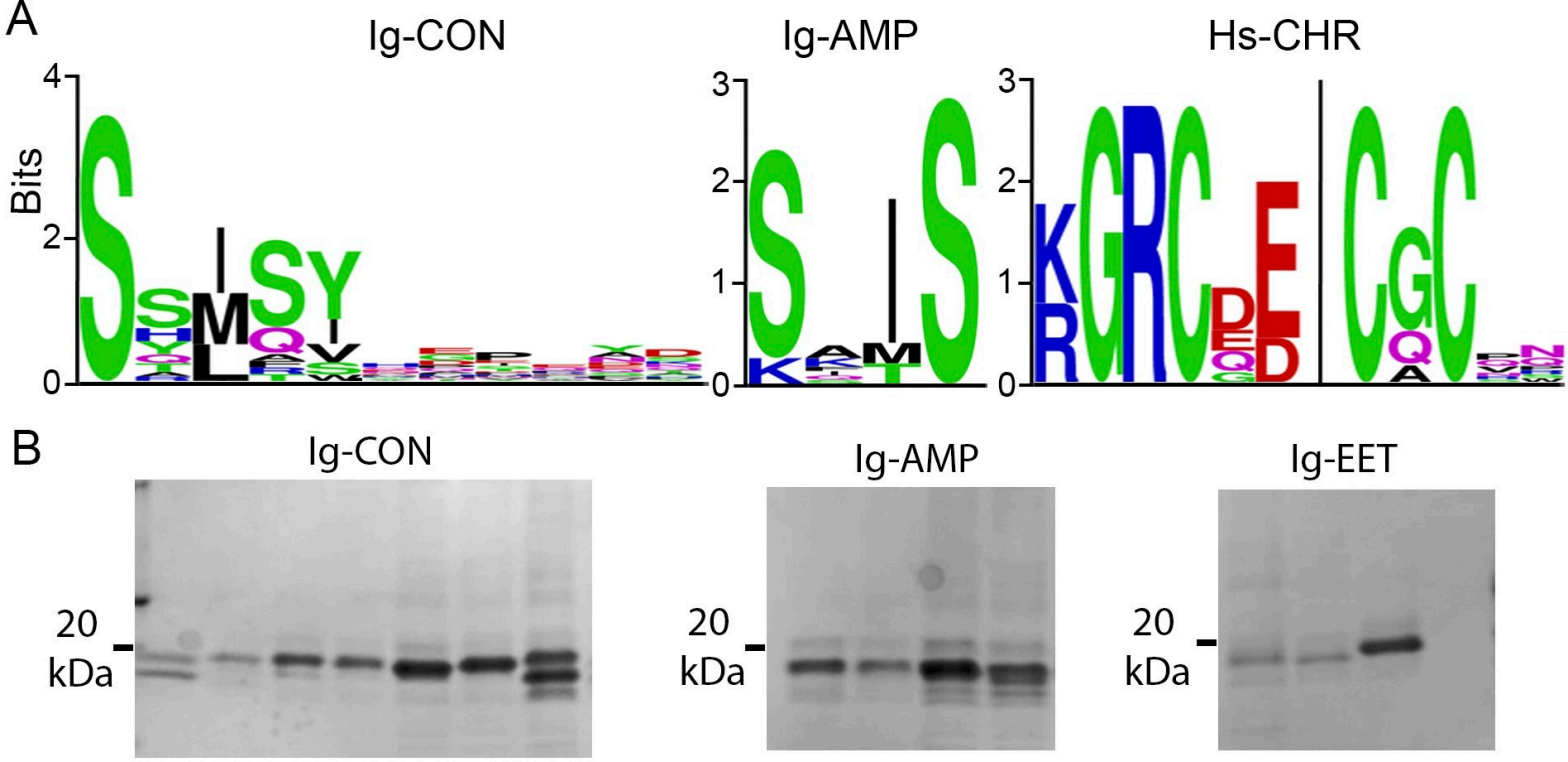
Identification of DCP binders to human IgG Fc or human serum albumin. (A) Representative sequence consensus analysis of top binders from phage spot ELISA. From left to right: Ig-CON DCPs (loop 2, 11 aa variants, n = 21); Ig-AMP DCPs (loop 2, 4 aa variants, n = 9); Hs-CHR DCPs (loop 3, 4, 5 and N-term, n = 9). (B) Recombinant production of Ig-DCPs (MW: 16–17 kD). Representative SDS-PAGE analysis of purified His(6)-SUMO-TEV-DCPs. Left panel: Ig-CON-1, MW: 16276.2 kDa; Ig-CON-2, 16625.6 kDa; Ig-CON-3, 16451.3 kDa; Ig-CON-4, 16283.2 kDa; Ig-CON-5, 15994.9 kDa; Ig-CON-6, 16438.4 kDa; Ig-CON-7, 16629.5 kDa; middle panel: Ig-AMP-1, MW: 16114.1 kDa; Ig-AMP-2, MW: 16057 kDa; Ig-AMP-3, MW: 16114 kDa; Ig-AMP-4, MW: 16073 kDa; right panel: Ig-EET-1, MW: 16555.5 kDa; Ig-EET-2, MW: 16306.2 kDa; Ig-EET-3, MW: 16171.1 kDa; Ig-EET-4, MW: 16415.4 kDa.

We tested our expanded DCP phage platform by screening for DCP binders against two plasma proteins, human IgG Fc and human serum albumin. After four rounds of selection, 1000 X, 100 X, or 30 X enrichment (calculated through dividing the number of binder phage colonies against the target by those against the control) was observed for panning against human IgG Fc using libraries Lib 1, Lib E, or Lib 2, respectively (S8 Fig in S1 File). For selection against human serum albumin, 30 X and 50 X enrichment was observed after four rounds of panning using Lib 1 or E, respectively. We then analyzed the target-binding phage using phage spot ELISA/Sanger sequencing and NGS (S9, S10 Figs in S1 File, S3-S10 Tables in S2 File). NGS offers high resolution overviews of the large sequence collections [59], while Sanger sequencing, together with phage spot ELISA, provides preliminary binding data of the lead peptides. Sanger sequencing and NGS analysis revealed several unique conserved sequence motifs from various DCP scaffolds such as AMP-1 (Ig-AMP, against human IgG Fc), conotoxin-MVIIA (Ig-CON, against human IgG Fc), EETI-II (Ig-EET, against human IgG Fc) and Charybdotoxin (Hs-CHR, against human serum albumin) (Fig 2A and S9, S10 Figs in S1 File). Interestingly, the same sequence motif “**S-X-I-S**” was identified from two different DCP scaffolds (conotoxin-MVIIA and AMP-1) through independent selection process (library group 1 and 2) (Fig 2A and S9, S10 Figs in S1 File), suggesting that this is likely a strong binding motif to IgG Fc and that Ig-CON and Ig-AMP DCPs might share overlapping epitopes on the target. Furthermore, Hs-CHR DCPs, containing the sequence motif “**D/E-E/D-I-C**” (Fig 2A), partially share a similar sequence motif to SA21, a previously reported human serum albumin binding peptide (RLI**EDIC**LPRWGCLWEDD) [10]. We then ranked the hits based on the information obtained from phage spot ELISA, Sanger sequencing and NGS, and selected top DCP variants for expression/folding analysis using the high throughput recombinant triaging platform described above. Analysis of the SUMO tagged DCPs by SDS-PAGE (Fig 2B and S11 Fig in S1 File) showed that majority of overexpressed DCP fusion protein contained minimal degradation, a sign of good stability. Further examination of the TEV protease treated samples using LC-MS revealed that > 70% of the selected variants (for both Ig-DCPs and Hs-DCPs) contained DCP products with three disulfide bonds (S12 Fig in S1 File and S11 Table in S2 File). We then selected the most interesting sequences based on binding (phage spot ELISA, Sanger sequencing and NGS) and expression data, and chemically synthesized/folded these DCPs (S13 Fig in S1 File and S12 Table in S2 File). The DCPs were chemically synthesized as linear peptides and folded in the optimized buffer system, desalted and purified with reversed phase HPLC. LC-MS was used to confirm the identity and high purity (> 97%) of the peptides (S13 Fig in S1 File).

### DCPs bind to human IgG1 Fc or human serum albumin with sub-micromolar affinity

**Fig 3:**
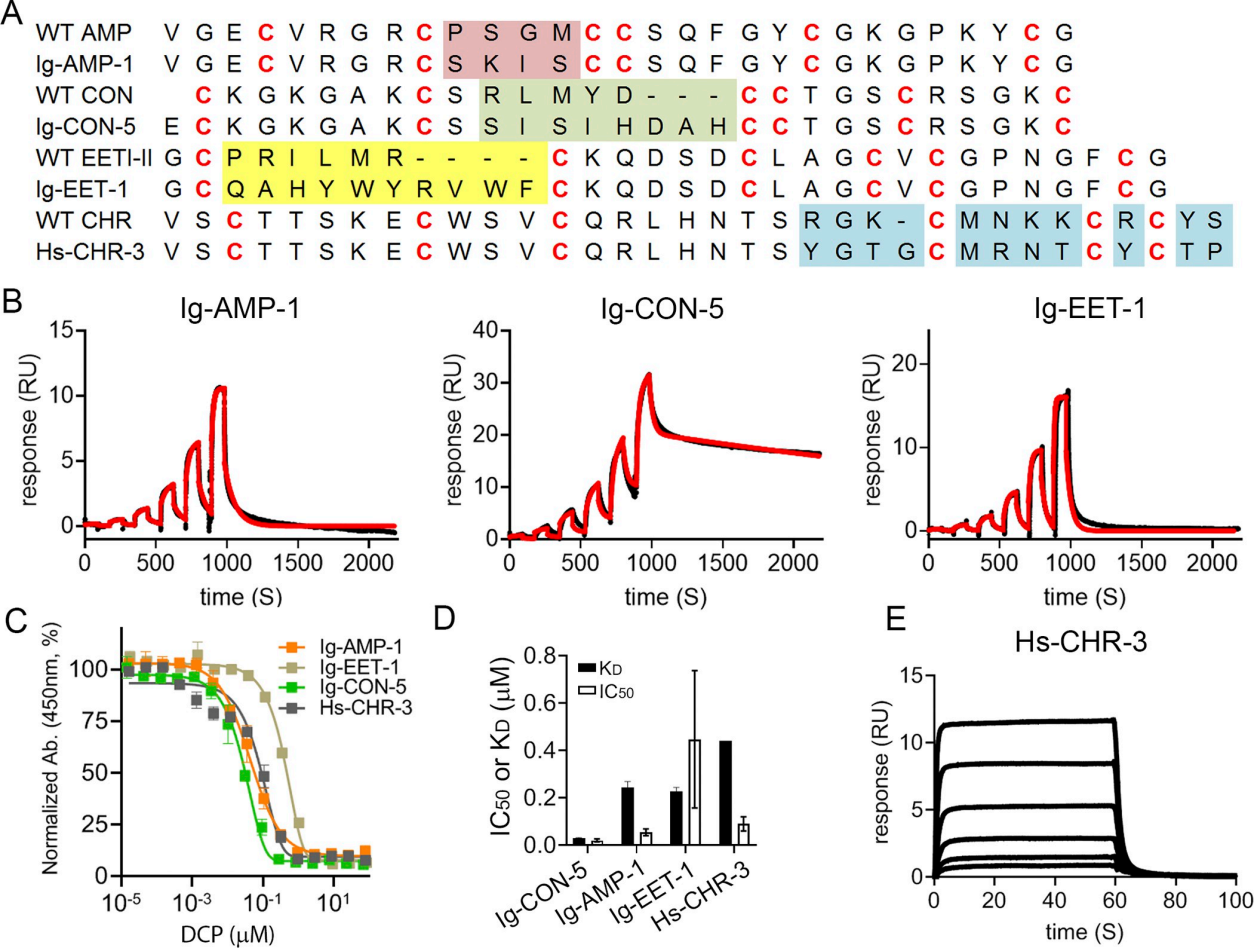
Synthetic DCPs bind to human IgG Fc or human serum albumin with sub-micromolar affinity. (A) Sequences of top DCP binders to human IgG Fc or human serum albumin. Loops that have been changed from the wild-type DCP sequences are highlighted in color. DCP variants with signal (binding against the target) to noise (binding against the control) (s/n) ratios (from phage spot ELISA) higher than 4 were selected and tested according to the procedure illustrated in Fig 1D, and the lead DCPs were chemically synthesized and analyzed with phage ELISA and SPR to identify the DCPs with the highest binding affinity from each scaffold. (B) Representative SPR sensorgrams showing dose response binding of top DCP binders to immobilized human IgG Fc (measured by single-cycle kinetics). Black: raw data; red: fitted data. (C) Phage competition ELISA shows synthetic DCPs compete with DCPs displayed on phage surface for binding against human IgG Fc or serum albumin. (D) Comparison of IC_50_ and K_D_ values of the top DCP binders against human IgG Fc or serum albumin. Open bar, IC_50_ values from phage ELISA; filled bar, K_D_ values from SPR. All values were averaged from three independent runs. Error bar: standard deviation from at least three independent experiments. (E) Representative SPR sensorgram showing dose response binding of Hs-CHR-3 to immobilized human serum albumin (measured by multi-cycle kinetics with a 3-fold serial dilution starting from 5 μM).

We then measured the IgG Fc binding affinity of these synthetic DCPs. Using SPR, we identified binders against human IgG Fc with sub-micromolar affinity (Fig 3A and 3B and S12 Table in S2 File). We also showed that the synthetic DCPs can compete with their own phage counterparts for binding to their cognate target proteins immobilized on the plate using phage competition ELISA (Fig 3C and 3D and S12 Table in S2 File). These results indicate that the synthetic DCPs shared the same folds and binding mode as DCPs displaying on phage surface. Furthermore, we performed large scale bioproduction of Ig-CON-5 to show that recombinant Ig-CON-5 shared similar affinity to synthetic Ig-CON-5 (S14 Fig in S1 File).

For the selection against human serum albumin, several synthetic DCPs from the Charybdotoxin scaffold demonstrated binding to human serum albumin in phage competition ELISA, with Hs-CHR-3 showing the lowest IC_50_ (Fig 3C and 3D and S12 Table in S2 File). SPR analysis further confirmed the result (K_D_ = 0.44 μM) (Fig 3A and 3E and S12 Table in S2 File). We speculate the lower success rate of human serum albumin panning (binders derived from only one scaffold) could be due to the interference of non-specific binding between the target protein and phage particles (rather than specific peptides displayed on them). This could be circumvented by modifying the panning protocol with more stringent washing conditions to reduce non-specific binders.

### Ig-DCPs and Hs-DCPs display high specificity to their targets and are stable in human serum

**Fig 4:**
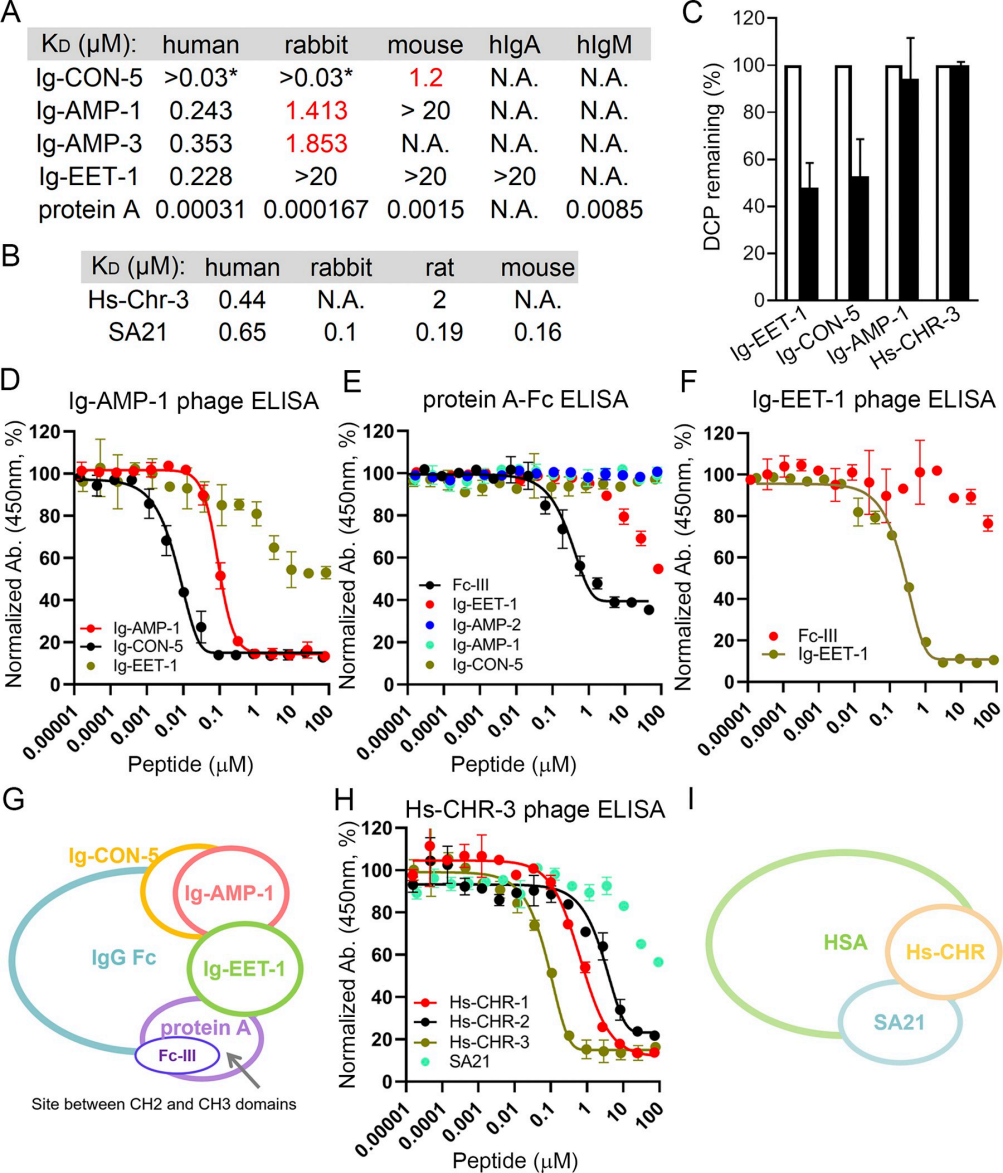
DCPs bind at distinct epitopes on IgG and human serum albumin. (A) Ig-DCPs bind less potently to rabbit and very weakly to mouse IgG Fc. They specifically bind to human IgG Fc over human IgA or IgM. Protein A was used as a control. Red: estimated values due to poor fitting. *: estimated value due to possible non-specific binding. (B) Hs-Chr-3 binds to human and rat (weakly), but not to rabbit or mouse albumin (N.A., not applicable). All K_D_ values were obtained using SPR. SA21 was used as a control. (C) Select Ig- and Hs-DCPs are stable in 100% human serum for at least 24 h at 37°C. DCP samples were analyzed using LC-MS and percentage of remaining peptide was calculated. Open bar, 0 h; solid bar: 24 h. Error bar: standard deviation from three independent experiments. (D) Ig-AMP-1 and Ig-CON-5 bind to a similar epitope. In the phage competition ELISA assay, Ig-AMP-1 and Ig-CON-5, but not Ig-EET-1, potently inhibited the interaction between biotinylated hIgG Fc and phage displaying Ig-AMP-1. (E) In the protein A-Fc interaction ELISA, the hIgG Fc binding peptide, Fc-III, but not Ig-CON-5 or Ig-AMP-1, disrupted the protein A (immobilized on the plate)-hIgG Fc binding. Ig-EET-1 also partially disrupted the binding between protein A and hIgG Fc. (F) Ig-EET-1 binds to a different epitope from Fc-III. In the phage competition ELISA assay, Ig-EET-1, but not Fc-III, potently inhibited the interaction between biotinylated hIgG Fc and phage displaying Ig-EET-1. Error bar: standard deviation from duplicate samples. Representative data from at least three independent experiments are shown. (G) Model of the mode of action of Ig-EET-1, Ig-AMP-1, Ig-CON-5 and Fc-III. Fc-III binds to the protein A binding pocket (the hinge between CH2 and CH3 domains) on hIgG Fc, whereas Ig-EET-1 binds to an epitope partially overlapping with the protein A binding site. Fc-III and Ig-EET-1 do not share binding epitopes. Ig-CON-5 and Ig-AMP-1 largely share the same binding epitope on hIgG Fc, which is away from the protein A binding pocket. They both partially share binding epitopes with Ig-EET-1. (H) Hs-CHR DCPs share partial binding epitope with SA21. In the phage competition ELISA assay, all synthetic Hs-CHR DCPs potently inhibited the interaction between biotinylated human serum albumin (immobilized on the plate) and phage displaying Hs-CHR-3. SA21 partially disrupted the interaction. Error bar: standard deviation from duplicate samples. Representative data from at least three independent experiments are shown. (I) Model of the mode of action of Hs-CHR DCPs. Hs-DCPs bind to the similar epitope on human serum albumin, which partially overlap with the SA21 binding site.

To assess the cross-species specificity of the Ig-DCPs, we compared the binding affinity of Ig-CON-5, Ig-AMP-1, Ig-AMP-3 and Ig-EET-1 to human, rabbit, and mouse IgG Fc by SPR. Sequence alignment of human, rabbit, rat and mouse IgG Fc (gamma chain) reveal that human and rabbit IgG Fc share 72% identity while human and rat/mouse IgG Fc are less identical (65% and 64%) (S13 Table in S2 File). Indeed, in comparison with human IgG Fc, Ig-DCPs bind less potently to rabbit and very weakly to mouse IgG Fc (Fig 4A). In normal human serum, IgG accounts for ~80% of the total immunoglobulin, with ~15% IgA, ~5% IgM, ~0.2% IgD, and trace amount of IgE [60]. We tested the binding capacity of the DCPs against IgA and IgM using SPR and demonstrated that these peptides only bind to IgG (Fig 4A). Similarly, Hs-CHR-3 showed high specificity to human serum albumin, with lower or undetectable binding affinity against rabbit, rat and mouse serum albumin (Fig 4B), probably due to the relatively low sequence identity (71–72%) shared between these different species (S14 Table in S2 File). Taken together, the data here indicate the Ig-DCPs and Hs-DCPs are highly specific to their individual targets.

The stability of plasma protein binding peptides is critical for half-life extension. Degradation, aggregation, or enzymatic digestion of these peptides in human blood could lead to faster *in vivo* clearance. To examine the stability of these DCPs in human blood, we incubated Ig-EET-1, Ig-CON-5, Ig-AMP-1 and Hs-CHR-3 in 100% human serum for 24 h at 37°C, then quantified the percentages of remaining peptides using LC-MS. Two DCPs, Ig-AMP-1 and Hs-CHR-3, showed remarkable stability, with 100% of the original DCP molecules retained after 24 h at 37°C in human serum (Fig 4C). For Ig-EET-1 and Ig-CON-5, even though LC-MS analysis did not identify any small peptide fragments, it is still not clear if the 40–50% loss of peptides after 24 h is due to aggregation, degradation or enzymatic digestion of the peptides. Further analysis is required to elucidate the cause of the instability.

### DCPs bind to distinct epitopes on IgG Fc and human serum albumin

We then used phage competition ELISA to further investigate the binding mode of the DCPs. In this assay setup, various synthetic DCPs compete with phage displaying one specific DCP immobilized on the plate. As shown in Fig 4D and S15 Fig in S1 File, synthetic Ig-EET-1, but not Ig-AMP-1 or Ig-CON-5, blocked the interaction between phage displaying Ig-EET-1 and human IgG Fc (immobilized on the plate). Moreover, synthetic Ig-AMP-1 and Ig-CON-5, but not Ig-EET-1, potently inhibited the interaction between phage displaying Ig-AMP-1 or Ig-CON-5 and human IgG Fc (immobilized on the plate). These data indicate that Ig-AMP-1 and Ig-CON-5 bind to a similar epitope, which partially overlaps with the binding epitope of Ig-EET-1. The results are consistent with the observation that Ig-AMP-1 and Ig-CON-5, but not Ig-EET-1, share the same sequence motif “**S-X-I-S**” (Figs 2A and 3A).

To further map the binding epitopes of the Ig-DCPs on human IgG Fc, we developed a protein A-human IgG Fc competition ELISA. We took advantage of the fact that protein A binds at the CH2-CH3 interface of IgG Fc. Leveraging a previously identified human IgG Fc binding peptide, Fc-III [9], as a positive control, we showed that it disrupted the binding between protein A and human IgG Fc as expected. This observation is consistent with a previous report demonstrating that Fc-III binds to the interface between CH2 and CH3 domains of IgG Fc [9], a binding site recognized by a number of other proteins (including FcRn, protein A and protein G) (Fig 4E). DCP Ig-EET-1 partially disrupted the binding between protein A and human IgG Fc, indicating partial overlap between the binding sites of protein A and Ig-EET-1. In contrast, neither Ig-AMP-1 nor Ig-CON-5 competed with protein A, suggesting a different binding mode (Fig 4E). In the Ig-EET-1 phage competition ELISA, Fc-III did not compete with phage displaying Ig-EET-1 (Fig 4F), indicating they bind to different epitopes. Taking together, the data point to a model in which Ig-AMP-1 and Ig-CON-5 bind to a similar epitope that is distinct from the protein A binding site at the CH2-CH3 interface (Fig 4G). Ig-EET-1 binds to a region that partially overlaps with both the Ig-AMP-1/Ig-CON-5 and protein A sites, but does not compete with Fc-III (Fig 4G). We also demonstrated that synthetic Hs-CHR DCPs competed with the Hs-CHR-3 phage, and SA21 could modestly inhibit the binding between Hs-CHR-3 phage and human serum albumin (Fig 4H). The findings imply Hs-CHR-1 and -3 bind to a similar epitope on human serum albumin, which partially overlap with the SA21 binding site (Fig 4I).

### Accelerated discovery of DCPs against VEGF-A (V-DCP), PDGF (P-DCP) and Ly6E (L-DCP)

**Fig 5:**
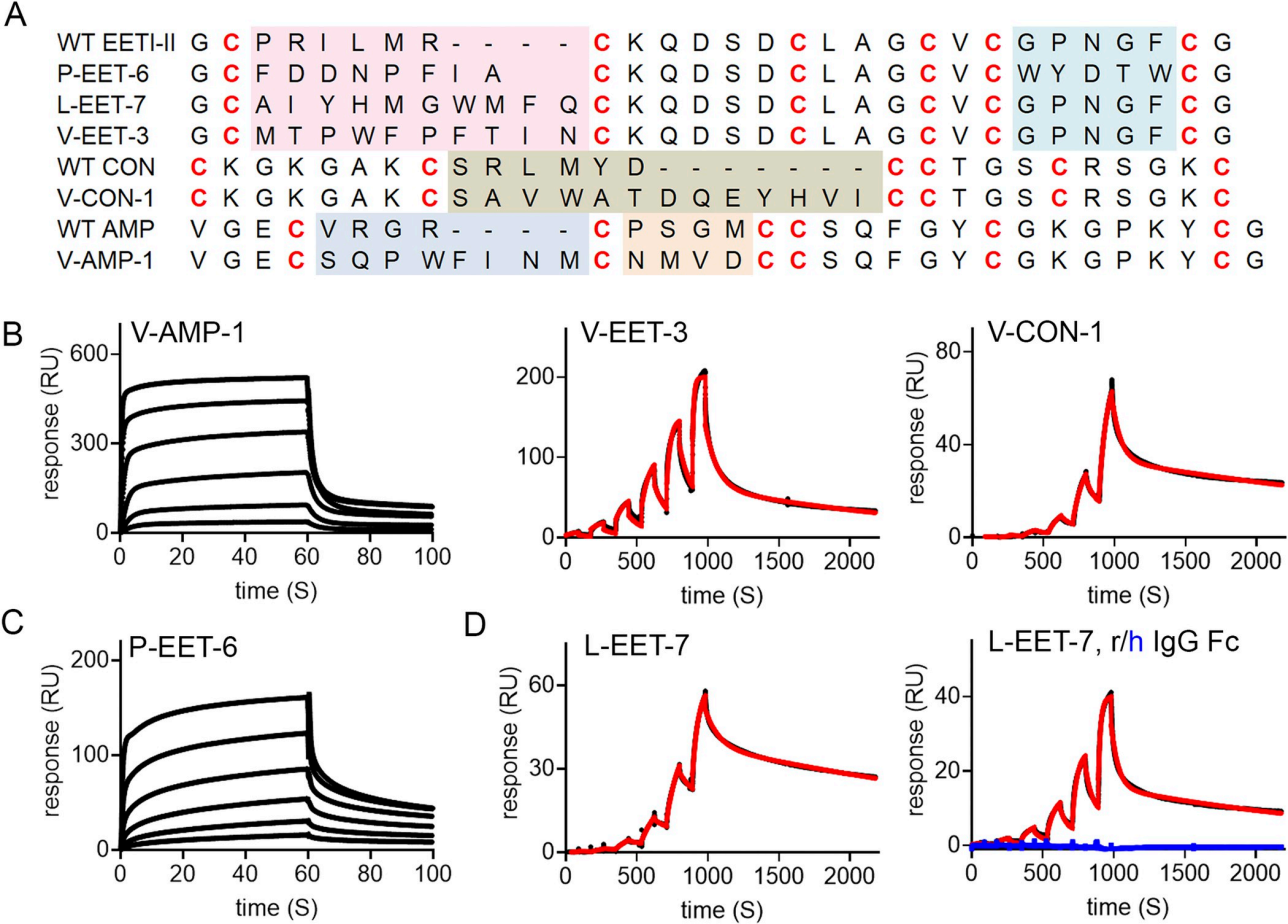
Discovery of DCP binders to human VEGF-A (V-DCP), human PDGF (P-DCP) or Ly6E (L-DCP). (A) Sequences of V-DCP, P-DCP or L-DCP. Loops that have been changed from the wild-type DCP sequences are highlighted in color. (B, C) Representative SPR sensorgrams showing dose response binding of top DCP binders to immobilized protein targets (V-EET-3 and V-CON-1 were measured by single-cycle kinetics; V-AMP-1 and P-EET-6 were measured by multi-cycle kinetics with a 3-fold serial dilution starting from 50 μM). Black: raw data; red: fitted data. (D) Representative SPR sensorgrams showing dose response binding of L-EET-7 to immobilized Ly6E-rat IgG Fc (left panel; black: raw data; red: fitted data), or rat/human IgG Fc (right panel; black: raw data against rat IgG Fc; red: fitted data against rat IgG Fc; blue: raw data against human IgG Fc). All were measured by single-cycle kinetics.

With the current set up of the discovery platform, it usually takes several weeks to complete the process. To improve the efficiency of the platform, we explored if the discovery timeline can be accelerated. As a test case, we used the libraries to select against three structurally and functionally different proteins, human VEGF-A, PDGF, and Ly6E. Here we relied heavily on NGS analysis (S14, S15 Tables in S2 File) to speed up the hit identification process. For VEGF-A and PDGF, we used biotinylated proteins. For Ly6E, we used biotinylated Ly6E fused to rat Fc to evaluate if different forms of protein would affect the selection outcome. Using the information from NGS, we were able to identify DCP binders against VEGF-A from three different scaffolds (Fig 5A and 5B and S16 Table in S2 File), namely AMP-1 (V-AMP), EETI-II (V-EET), and conotoxin (V-CON), and a binder against PDGF from the EETI-II scaffold (P-EET-6, Fig 5A and 5C and S16 Table in S2 File). It is perhaps not surprising that the DCP against Ly6E-rat Fc identified from the EETI-II scaffold, L-EET-7, also binds to rat Fc with a similar affinity (Fig 5D and S17 Table in S2 File), since we have demonstrated that DCPs against human IgG Fc can be identified from multiple scaffolds. L-EET-7 specifically binds to rat Fc over human Fc, consistent with our findings that DCPs against Fc show good cross-species specificity (Fig 4A). The data indicate that while different forms of proteins might offer certain advantages (such as flexibility for immobilization), caution needs to be taken to ensure specificity during the selection process. Under circumstances where the Fc-fused protein is the desired form, a counter-selection with free Fc protein should be performed to eliminate binders against Fc. Nevertheless, the findings here demonstrated that the extended DCP phage libraries can be used to identify binders against a wide range of protein antigens, and accelerated discovery of DCP binder sequences (in ~5 days) can be achieved using NGS analysis alone.

## Discussion

In this study, we describe a robust and highly effective DCP phage display platform with improved diversity to identify DCP-derived binders against targets of therapeutic interest. The efficient selection process combined with recombinant and chemical production of the hits yields specific and stable DCP binders. Using this platform, we identified new binders against different proteins (including human IgG Fc, serum albumin, VEGF-A and PDGF) from multiple DCP scaffold backgrounds. The Ig- and Hs-DCPs showed sub-micromolar affinity against their targets. They demonstrated excellent species/isoform specificity and were stable in human serum. Identification of these human plasma protein DCP binders exemplifies the application of the platform, and could offer alternative tools for half-life extension of peptides and small proteins in general.

Here we expanded the DCP phage libraries by selecting five new scaffolds (Mch-1, gurmarin, Asteropsin-A, AMP-1, and CPI) with different biochemical/biophysical and pharmacological properties to enhance the diversity of the libraries. The high diversity of the extended libraries not only contributes to the high success rate of the selection, but it also helps to obtain a panel of binders with broad topology, epitope coverage, and biochemical/biophysical properties, partially inherited from the parent wild-type DCPs. From the primary panning against human IgG Fc, we identified sub-micromolar DCP binders from three different DCP scaffolds, namely Ig-CON-5, Ig-AMP-1, and Ig-EET-1. It is noteworthy that in all three lead DCP binders, only one single loop of the wild-type scaffold was modified (Fig 3A). This leaves room for additional affinity maturation at other loop regions. For example, in Ig-CON-5, loop 1, 3, and 4 can be subjected to hard randomization to enhance the affinity, and all the loops can be soft randomized to further fine tune the affinity [35]. Moreover, unnatural amino acids could be readily incorporated into these DCPs, unlike antibodies. This offers another means to improve desired properties including affinity, solubility or stability. Other approaches towards optimization of DCP binders could also be incorporated into the platform due to the versatility of the panning process. The panning protocol could be modified to include stringent washing steps, protease digestion or temperature controlled incubation to screen for binders with high affinity or stability. Furthermore, when coupled with whole cell panning, the platform could be applied to selection of DCPs binding to specific cancer cells [61] or internalizing into specific subcellular locations [62].

Some of the wild-type DCPs described in this work have previously been produced by synthetic chemical methods. For instance, gurmarin was produced by thermodynamic oxidation of the linear peptide in a Tris–HCl based redox buffer, or by using orthogonal cysteine side chain protecting groups allowing stepwise selective formation of disulfide bonds [63]. We prefer thermodynamic folding over the orthogonal oxidation method for most cases due to the overall yield, time, cost, and simplicity of the approach [63]. The disadvantage of this method, however, is that different DCPs often require different folding conditions. To circumvent this, through extensive evaluation of different oxidation buffer systems, we identified two nearly universal buffer systems in which a large number of synthetic linear DCP hits could be folded systematically and efficiently. We further showed that the chemically synthesized/folded DCPs could compete with DCPs displayed on phage for binding to their targets (Fig 3), suggesting that they shared the same overall fold.

Although the synthetic method is great for UAA incorporation, DCP synthesis is often associated with high cost, especially with a large number of hits. Recombinant DCP production offers an alternative way to generate DCPs in a streamlined and high throughput manner. Production of functional DCPs in *E*. *coli* requires formation of the disulfide bonds. It has been shown that for Fab production, the PhoA promoter has higher secretion ability than the T7 promoter, and the signal peptide STII demonstrate higher extracellular secretion efficiency than pelB [64, 65]. We took advantage of the PhoA-STII expression system where the DCP construct is targeted to the periplasmic space for oxidative folding, providing a universal method for biological production of DCPs with good yield and high throughput. The method also enables scalability and the production of isotope (^15^N/^13^C) labeled DCPs [52] for NMR studies. Information obtained from structure-activity relationship studies using NMR spectroscopy will be of great use to guide affinity maturation or stability improvement of the lead DCP molecules.

The use of plasma protein binding ligands has proven to be an effective strategy to achieve half-life extension of peptide and small protein therapeutics. Conjugation of chemical moieties, such as lipids or PEG chains, has been extensively used for this purpose [66, 67]. However, the long-term safety of PEGylation remains elusive [68]. Lipidation also exhibits other inherent limitations such as hydrophobicity and lack of specificity [69]. Peptides that bind to plasma proteins could circumvent these shortcomings, but they also have liabilities such as low serum stability, thereby limiting their use in systemic therapeutic applications [70, 71]. During drug discovery campaigns, tremendous efforts are typically dedicated to stabilize peptides against proteases that are present in serum. Replacing the labile natural amino acids with unnatural amino acids is one of the preferred methods to improve peptide stability [72]. For instance, a recent study demonstrated that the intestinal stability of oxytocin (OT) can be dramatically improved (*t*_1/2_^SIF^ >24 h (SIF–simulated intestinal fluid)) by grafting OT-like sequences into loops of inhibitory cystine-knot (ICK) peptides [73]. Here we developed stable DCP-based plasma protein binding peptides (Ig-AMP-1 and Hs-CHR-3) without engineering UAAs into the peptide sequence. The intrinsic stability introduced by their disulfide constrained conformation, together with the great specificity, makes CKPs/DCPs attractive scaffolds for development of half-life extension tools. It is noteworthy that future exploration of the *in vivo* half-life and immunogenicity of small proteins conjugated to these DCPs is warranted, as binding to serum proteins with longer half-life could alter the immunogenicity of the peptides.

In summary, the DCP discovery platform described encompassing phage-displayed DCP libraries combined with small-scale high throughput recombinant production and large-scale synthetic production enables rapid identification of DCP binders that could have applications in peptide therapeutics development. In our experience, the high diversity and different conformations of the DCP binders could be beneficial in cases where antibody discovery campaigns failed to yield functional molecules and therefore serve as an alternative modality for controlling the function of target proteins and their signaling in cells.

## Supporting information

S1 File (PDF)

S2 File (PDF)

## References

[1] Lau JL, Dunn MK (2018) Therapeutic peptides: Historical perspectives, current development trends, and future directions. Bioorg Med Chem 26: 2700–2707. doi: 10.1016/j.bmc.2017.06.052 28720325

[2] Muttenthaler M, King GF, Adams DJ, Alewood PF (2021) Trends in peptide drug discovery. Nat Rev Drug Discov 20: 309–325. doi: 10.1038/s41573-020-00135-8 33536635

[3] McGregor DP (2008) Discovering and improving novel peptide therapeutics. Curr Opin Pharmacol 8: 616–619. doi: 10.1016/j.coph.2008.06.002 18602024

[4] Zorzi A, Linciano S, Angelini A (2019) Non-covalent albumin-binding ligands for extending the circulating half-life of small biotherapeutics. Medchemcomm 10: 1068–1081. doi: 10.1039/c9md00018f 31391879 PMC6644573

[5] Home P, Kurtzhals P (2006) Insulin detemir: from concept to clinical experience. Expert Opin Pharmacother 7: 325–343. doi: 10.1517/14656566.7.3.325 16448327

[6] Le Floch JP (2010) Critical appraisal of the safety and efficacy of insulin detemir in glycemic control and cardiovascular risk management in diabetics. Diabetes Metab Syndr Obes 3: 197–213. doi: 10.2147/dmsott.s7315 21437089 PMC3047990

[7] Owens DR (2011) Insulin preparations with prolonged effect. Diabetes Technol Ther 13 Suppl 1: S5–14. doi: 10.1089/dia.2011.0068 21668337

[8] Datta-Mannan A, Boyles J, Huang L, Jin ZY, Peariso A, et al. (2019) Engineered FcRn Binding Fusion Peptides Significantly Enhance the Half-Life of a Fab Domain in Cynomolgus Monkeys. Biotechnol J 14: e1800007. 29802766 10.1002/biot.201800007

[9] DeLano WL, Ultsch MH, de Vos AM, Wells JA (2000) Convergent solutions to binding at a protein-protein interface. Science 287: 1279–1283. doi: 10.1126/science.287.5456.1279 10678837

[10] Dennis MS, Zhang M, Meng YG, Kadkhodayan M, Kirchhofer D, et al. (2002) Albumin binding as a general strategy for improving the pharmacokinetics of proteins. J Biol Chem 277: 35035–35043. doi: 10.1074/jbc.M205854200 12119302

[11] Ackerman SE, Currier NV, Bergen JM, Cochran JR (2014) Cystine-knot peptides: emerging tools for cancer imaging and therapy. Expert Rev Proteomics 11: 561–572. doi: 10.1586/14789450.2014.932251 25163524

[12] Agwa AJ, Huang YH, Craik DJ, Henriques ST, Schroeder CI (2017) Lengths of the C-Terminus and Interconnecting Loops Impact Stability of Spider-Derived Gating Modifier Toxins. Toxins (Basel) 9. doi: 10.3390/toxins9080248 28805686 PMC5577582

[13] Colgrave ML, Craik DJ (2004) Thermal, chemical, and enzymatic stability of the cyclotide kalata B1: the importance of the cyclic cystine knot. Biochemistry 43: 5965–5975. doi: 10.1021/bi049711q 15147180

[14] Craik DJ, Daly NL, Waine C (2001) The cystine knot motif in toxins and implications for drug design. Toxicon 39: 43–60. doi: 10.1016/s0041-0101(00)00160-4 10936622

[15] Gao XX, Stanger K, Kaluarachchi H, Maurer T, Ciepla P, et al. (2016) Cellular uptake of a cystine-knot peptide and modulation of its intracellular trafficking. Scientific Reports 6. doi: 10.1038/srep35179 27734922 PMC5062073

[16] Herzig V, King GF (2015) The Cystine Knot Is Responsible for the Exceptional Stability of the Insecticidal Spider Toxin omega-Hexatoxin-Hv1a. Toxins (Basel) 7: 4366–4380. 26516914 10.3390/toxins7104366 PMC4626739

[17] Stanger K, Maurer T, Kaluarachchi H, Coons M, Franke Y, et al. (2014) Backbone cyclization of a recombinant cystine-knot peptide by engineered Sortase A. FEBS Lett 588: 4487–4496. doi: 10.1016/j.febslet.2014.10.020 25448598

[18] Daly NL, Craik DJ (2011) Bioactive cystine knot proteins. Curr Opin Chem Biol 15: 362–368. doi: 10.1016/j.cbpa.2011.02.008 21362584

[19] Rauck RL, Wallace MS, Leong MS, Minehart M, Webster LR, et al. (2006) A randomized, double-blind, placebo-controlled study of intrathecal ziconotide in adults with severe chronic pain. J Pain Symptom Manage 31: 393–406. doi: 10.1016/j.jpainsymman.2005.10.003 16716870

[20] Williams JA, Day M, Heavner JE (2008) Ziconotide: an update and review. Expert Opin Pharmacother 9: 1575–1583. doi: 10.1517/14656566.9.9.1575 18518786

[21] Lembo AJ, Kurtz CB, Macdougall JE, Lavins BJ, Currie MG, et al. (2010) Efficacy of linaclotide for patients with chronic constipation. Gastroenterology 138: 886–895 e881. doi: 10.1053/j.gastro.2009.12.050 20045700

[22] D’Souza C, Henriques ST, Wang CK, Cheneval O, Chan LY, et al. (2016) Using the MCoTI-II Cyclotide Scaffold To Design a Stable Cyclic Peptide Antagonist of SET, a Protein Overexpressed in Human Cancer. Biochemistry 55: 396–405. doi: 10.1021/acs.biochem.5b00529 26685975

[23] Hilpert K, Wessner H, Schneider-Mergener J, Welfle K, Misselwitz R, et al. (2003) Design and characterization of a hybrid miniprotein that specifically inhibits porcine pancreatic elastase. J Biol Chem 278: 24986–24993. doi: 10.1074/jbc.M212152200 12700244

[24] Kimura RH, Jones DS, Jiang L, Miao Z, Cheng Z, et al. (2011) Functional mutation of multiple solvent exposed loops in the Ecballium elaterium trypsin inhibitor-II cystine knot miniprotein. PLoS ONE 6: e16112. doi: 10.1371/journal.pone.0016112 21364742 PMC3041754

[25] Kimura RH, Levin AM, Cochran FV, Cochran JR (2009) Engineered cystine knot peptides that bind alphavbeta3, alphavbeta5, and alpha5beta1 integrins with low-nanomolar affinity. Proteins 77: 359–369. doi: 10.1002/prot.22441 19452550 PMC5792193

[26] Krause S, Schmoldt H-U, Wentzel A, Ballmaier M, Friedrich K, et al. (2007) Grafting of thrombopoietin-mimetic peptides into cystine knot miniproteins yields high-affinity thrombopoietin antagonists and agonists. FEBS J 274: 86–95. doi: 10.1111/j.1742-4658.2006.05567.x 17147697

[27] Garcia AE, Camarero JA (2010) Biological activities of natural and engineered cyclotides, a novel molecular scaffold for peptide-based therapeutics. Curr Mol Pharmacol 3: 153–163. doi: 10.2174/1874467211003030153 20858197 PMC3328131

[28] Waldmann H, Valeur E, Gueret SM, Adihou H, Gopalakrishnan R, et al. (2017) New Modalities for Challenging Targets in Drug Discovery. Angew Chem Int Ed Engl. doi: 10.1002/anie.201611914 28186380

[29] Liu W, de Veer SJ, Huang YH, Sengoku T, Okada C, et al. (2021) An Ultrapotent and Selective Cyclic Peptide Inhibitor of Human beta-Factor XIIa in a Cyclotide Scaffold. J Am Chem Soc 143: 18481–18489. 34723512 10.1021/jacs.1c07574

[30] Sidhu SS (2000) Phage display in pharmaceutical biotechnology. Curr Opin Biotechnol 11: 610–616. doi: 10.1016/s0958-1669(00)00152-x 11102798

[31] Smith GP (1985) Filamentous fusion phage: novel expression vectors that display cloned antigens on the virion surface. Science 228: 1315–1317. doi: 10.1126/science.4001944 4001944

[32] Tonikian R, Zhang Y, Boone C, Sidhu SS (2007) Identifying specificity profiles for peptide recognition modules from phage-displayed peptide libraries. Nat Protoc 2: 1368–1386. doi: 10.1038/nprot.2007.151 17545975

[33] Pande J, Szewczyk MM, Grover AK (2010) Phage display: concept, innovations, applications and future. Biotechnol Adv 28: 849–858. doi: 10.1016/j.biotechadv.2010.07.004 20659548

[34] Wu CH, Liu IJ, Lu RM, Wu HC (2016) Advancement and applications of peptide phage display technology in biomedical science. J Biomed Sci 23: 8. doi: 10.1186/s12929-016-0223-x 26786672 PMC4717660

[35] Hansen S, Zhang Y, Hwang S, Nabhan A, Li W, et al. (2022) Directed evolution identifies high-affinity cystine-knot peptide agonists and antagonists of Wnt/beta-catenin signaling. Proc Natl Acad Sci U S A 119: e2207327119. 36343233 10.1073/pnas.2207327119 PMC9674260

[36] Zhou L, Cai F, Li Y, Gao X, Wei Y, et al. (2024) Disulfide-constrained peptide scaffolds enable a robust peptide-therapeutic discovery platform. doi: 10.1371/journal.pone.0300135 PMC10977697 38547109

[37] He WJ, Chan LY, Clark RJ, Tang J, Zeng GZ, et al. (2013) Novel inhibitor cystine knot peptides from Momordica charantia. PLoS One 8: e75334. doi: 10.1371/journal.pone.0075334 24116036 PMC3792974

[38] Imoto T, Miyasaka A, Ishima R, Akasaka K (1991) A novel peptide isolated from the leaves of Gymnema sylvestre—I. Characterization and its suppressive effect on the neural responses to sweet taste stimuli in the rat. Comp Biochem Physiol A Comp Physiol 100: 309–314. doi: 10.1016/0300-9629(91)90475-r 1685952

[39] Li H, Bowling JJ, Fronczek FR, Hong J, Jabba SV, et al. (2013) Asteropsin A: an unusual cystine-crosslinked peptide from porifera enhances neuronal Ca2+ influx. Biochim Biophys Acta 1830: 2591–2599. doi: 10.1016/j.bbagen.2012.11.015 23201194 PMC4137556

[40] Broekaert WF, Marien W, Terras FR, De Bolle MF, Proost P, et al. (1992) Antimicrobial peptides from Amaranthus caudatus seeds with sequence homology to the cysteine/glycine-rich domain of chitin-binding proteins. Biochemistry 31: 4308–4314. doi: 10.1021/bi00132a023 1567877

[41] Blanco-Aparicio C, Molina MA, Fernandez-Salas E, Frazier ML, Mas JM, et al. (1998) Potato carboxypeptidase inhibitor, a T-knot protein, is an epidermal growth factor antagonist that inhibits tumor cell growth. J Biol Chem 273: 12370–12377. doi: 10.1074/jbc.273.20.12370 9575190

[42] Gao X, Maziere A, Beard R, Klumperman J, Hannoush RN (2021) Fatty acylation enhances the cellular internalization and cytosolic distribution of a cystine-knot peptide. iScience 24: 103220. doi: 10.1016/j.isci.2021.103220 34712919 PMC8529511

[43] Kschonsak YT, Gao X, Miller SE, Hwang S, Marei H, et al. (2024) Potent and selective binders of the E3 ubiquitin ligase ZNRF3 stimulate Wnt signaling and intestinal organoid growth. Cell Chem Biol 31: 1–12. 10.1016/j.chembiol.2023.11.006 38056465

[44] Hansen S, Nile AH, Mehta SC, Fuhrmann J, Hannoush RN (2019) Lead Optimization Yields High Affinity Frizzled 7-Targeting Peptides That Modulate Clostridium difficile Toxin B Pathogenicity in Epithelial Cells. J Med Chem 62: 7739–7750. doi: 10.1021/acs.jmedchem.9b00500 31429553

[45] Li H, Bowling JJ, Su M, Hong J, Lee BJ, et al. (2014) Asteropsins B-D, sponge-derived knottins with potential utility as a novel scaffold for oral peptide drugs. Biochim Biophys Acta 1840: 977–984. doi: 10.1016/j.bbagen.2013.11.001 24225326 PMC4139099

[46] Hass GM, Ryan CA (1981) Carboxypeptidase Inhibitor from Potatoes. Methods in Enzymology 80: 778–791.

[47] Chavez MI, Andreu C, Vidal P, Aboitiz N, Freire F, et al. (2005) On the importance of carbohydrate-aromatic interactions for the molecular recognition of oligosaccharides by proteins: NMR studies of the structure and binding affinity of AcAMP2-like peptides with non-natural naphthyl and fluoroaromatic residues. Chemistry-a European Journal 11: 7060–7074. doi: 10.1002/chem.200500367 16220560

[48] Daly NL, Gustafson KR, Craik DJ (2004) The role of the cyclic peptide backbone in the anti-HIV activity of the cyclotide kalata B1. FEBS Lett 574: 69–72. doi: 10.1016/j.febslet.2004.08.007 15358541

[49] Goransson U, Craik DJ (2003) Disulfide mapping of the cyclotide kalata B1—Chemical proof of the cyclic cystine knot motif. Journal of Biological Chemistry 278: 48188–48196. 12960160 10.1074/jbc.M308771200

[50] Getz JA, Rice JJ, Daugherty PS (2011) Protease-resistant peptide ligands from a knottin scaffold library. ACS Chem Biol 6: 837–844. doi: 10.1021/cb200039s 21615106 PMC3158827

[51] de Marco A (2009) Strategies for successful recombinant expression of disulfide bond-dependent proteins in Escherichia coli. Microb Cell Fact 8: 26. 19442264 10.1186/1475-2859-8-26 PMC2689190

[52] Klint JK, Senff S, Saez NJ, Seshadri R, Lau HY, et al. (2013) Production of recombinant disulfide-rich venom peptides for structural and functional analysis via expression in the periplasm of E. coli. PLoS One 8: e63865. doi: 10.1371/journal.pone.0063865 23667680 PMC3646780

[53] Nozach H, Fruchart-Gaillard C, Fenaille F, Beau F, Ramos OH, et al. (2013) High throughput screening identifies disulfide isomerase DsbC as a very efficient partner for recombinant expression of small disulfide-rich proteins in E. coli. Microb Cell Fact 12: 37. 23607455 10.1186/1475-2859-12-37 PMC3668227

[54] Simmons LC, Reilly D, Klimowski L, Raju TS, Meng G, et al. (2002) Expression of full-length immunoglobulins in Escherichia coli: rapid and efficient production of aglycosylated antibodies. J Immunol Methods 263: 133–147. doi: 10.1016/s0022-1759(02)00036-4 12009210

[55] Spiess C, Merchant M, Huang A, Zheng Z, Yang NY, et al. (2013) Bispecific antibodies with natural architecture produced by co-culture of bacteria expressing two distinct half-antibodies. Nat Biotechnol 31: 753–758. doi: 10.1038/nbt.2621 23831709

[56] Zhou Y, Liu P, Gan Y, Sandoval W, Katakam AK, et al. (2016) Enhancing full-length antibody production by signal peptide engineering. Microb Cell Fact 15: 47. doi: 10.1186/s12934-016-0445-3 26935575 PMC4776426

[57] Chang JY, Canals F, Schindler P, Querol E, Aviles FX (1994) The disulfide folding pathway of potato carboxypeptidase inhibitor. J Biol Chem 269: 22087–22094. 8071332

[58] Kamolkijkarn P, Prasertdee T, Netirojjanakul C, Sarnpitak P, Ruchirawat S, et al. (2010) Synthesis, biophysical, and biological studies of wild-type and mutant psalmopeotoxins-Anti-malarial cysteine knot peptides from Psalmopoeus cambridgei. Peptides 31: 533–540. doi: 10.1016/j.peptides.2010.01.001 20067814

[59] Ravn U, Didelot G, Venet S, Ng KT, Gueneau F, et al. (2013) Deep sequencing of phage display libraries to support antibody discovery. Methods 60: 99–110. doi: 10.1016/j.ymeth.2013.03.001 23500657

[60] Loh RK, Vale S, McLean-Tooke A (2013) Quantitative serum immunoglobulin tests. Aust Fam Physician 42: 195–198. 23550242

[61] McGuire MJ, Gray BP, Li S, Cupka D, Byers LA, et al. (2014) Identification and characterization of a suite of tumor targeting peptides for non-small cell lung cancer. Sci Rep 4: 4480. doi: 10.1038/srep04480 24670678 PMC3967199

[62] Umlauf BJ, Mercedes JS, Chung CY, Brown KC (2014) Identification of a novel lysosomal trafficking peptide using phage display biopanning coupled with endocytic selection pressure. Bioconjug Chem 25: 1829–1837. doi: 10.1021/bc500326x 25188559 PMC4198098

[63] Eliasen R, Andresen TL, Conde-Frieboes KW (2012) Handling a tricycle: orthogonal versus random oxidation of the tricyclic inhibitor cystine knotted peptide gurmarin. Peptides 37: 144–149. doi: 10.1016/j.peptides.2012.06.016 22771618

[64] Gundinger T, Kittler S, Kubicek S, Kopp J, Spadiut O (2022) Recombinant Protein Production in E. coli Using the phoA Expression System. Fermentation-Basel 8.

[65] Luo M, Zhao M, Cagliero C, Jiang H, Xie Y, et al. (2019) A general platform for efficient extracellular expression and purification of Fab from Escherichia coli. Appl Microbiol Biotechnol 103: 3341–3353. doi: 10.1007/s00253-019-09745-8 30887174

[66] Koehler MF, Zobel K, Beresini MH, Caris LD, Combs D, et al. (2002) Albumin affinity tags increase peptide half-life in vivo. Bioorg Med Chem Lett 12: 2883–2886. doi: 10.1016/s0960-894x(02)00610-8 12270169

[67] Ostergaard S, Paulsson JF, Kofoed J, Zosel F, Olsen J, et al. (2021) The effect of fatty diacid acylation of human PYY3-36 on Y2 receptor potency and half-life in minipigs. Sci Rep 11: 21179. doi: 10.1038/s41598-021-00654-3 34707178 PMC8551270

[68] van Witteloostuijn SB, Pedersen SL, Jensen KJ (2016) Half-Life Extension of Biopharmaceuticals using Chemical Methods: Alternatives to PEGylation. ChemMedChem 11: 2474–2495. doi: 10.1002/cmdc.201600374 27775236

[69] Bech EM, Pedersen SL, Jensen KJ (2018) Chemical Strategies for Half-Life Extension of Biopharmaceuticals: Lipidation and Its Alternatives. ACS Med Chem Lett 9: 577–580. doi: 10.1021/acsmedchemlett.8b00226 30034579 PMC6047018

[70] Bottger R, Hoffmann R, Knappe D (2017) Differential stability of therapeutic peptides with different proteolytic cleavage sites in blood, plasma and serum. PLoS One 12: e0178943. doi: 10.1371/journal.pone.0178943 28575099 PMC5456363

[71] Jenssen H, Aspmo SI (2008) Serum stability of peptides. Methods Mol Biol 494: 177–186. doi: 10.1007/978-1-59745-419-3_10 18726574

[72] Lu J, Xu H, Xia J, Ma J, Xu J, et al. (2020) D- and Unnatural Amino Acid Substituted Antimicrobial Peptides With Improved Proteolytic Resistance and Their Proteolytic Degradation Characteristics. Front Microbiol 11: 563030. doi: 10.3389/fmicb.2020.563030 33281761 PMC7688903

[73] Kremsmayr T, Aljnabi A, Blanco-Canosa JB, Tran HNT, Emidio NB, et al. (2022) On the Utility of Chemical Strategies to Improve Peptide Gut Stability. J Med Chem 65: 6191–6206. doi: 10.1021/acs.jmedchem.2c00094 35420805 PMC9059125

