# Supplementary material for: A phage-displayed disulfide constrained peptide discovery platform yields novel human plasma protein binders
Source: PLoS One. 2024 Mar 28;19(3):e0299804. doi: 10.1371/journal.pone.0299804 (PMC10977726; doi:10.1371/journal.pone.0299804)
Supplement: S1 File — (PDF) [file pone.0299804.s001.pdf]

Supporting Information

A phage-displayed disulfide constrained peptide discovery platform yields novel human plasma protein binders

Xinxin Gao[1,2]*, Harini Kaluarachchi[1], Yingnan Zhang[1,3], Sunhee Hwang[1,2] and Rami N. Hannoush[1]*

Departments of [1]Early Discovery Biochemistry, [2]Peptide Therapeutics, [3]Biological Chemistry, Genentech, South San Francisco, California

* Corresponding author: gao.xinxin@gene.com and ramihannoush@gmail.com

A

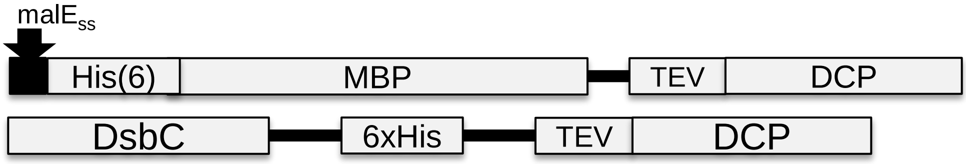

	DCP	Strain	Yield (mg/L)
1	kalata B1	Rosetta™2(DE3)	1-5
2	kalata Thrombin	Rosetta™2(DE3)	>10
3	AVR9	Rosetta™2(DE3)	>10
4	Circulin-A	Rosetta™2(DE3)	>10
5	Conotoxin-MVIIA	Rosetta™2(DE3)	0
6	Huwentoxin	Rosetta™2(DE3)	0
7	Min23	Rosetta™2(DE3)	>10
8	CBD	Rosetta™2(DE3)	>10
9	Charybdotoxin	Rosetta™2(DE3)	0
10	kalataB1	BL21(DE3)pLysS	>10
11	Min23	BL21(DE3)pLysS	>10
12	CBD	BL21(DE3)pLysS	>10

Fully oxidized DCP
Mixture of fully/partially oxidized DCP isomers
DCP not isolated successfully
DCP with modification(s)

B

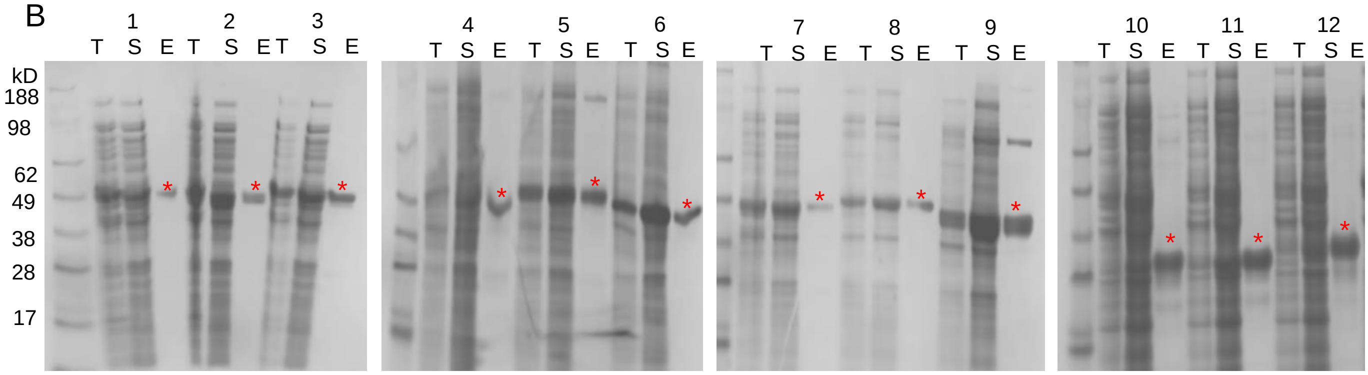

Figure S1. Summary (A) and SDS PAGE analysis (B) of pET vector expression of wild type DCPs with MBP or Dsbc at 17 °C for 24 h. Proteins were over-expressed in Rosetta™2(DE3) or BL21(DE3)pLysS cells and purified using Ni-IMAC tips on an automated liquid handler (Oasis). The folding profile (disulfide bond formation) was analyzed using LC-MS. Protein concentrations were calculated using their absorbance at 280 nm and individual extinction coefficient. T: total lysate; S: soluble fraction; E: elute; *: purified protein.

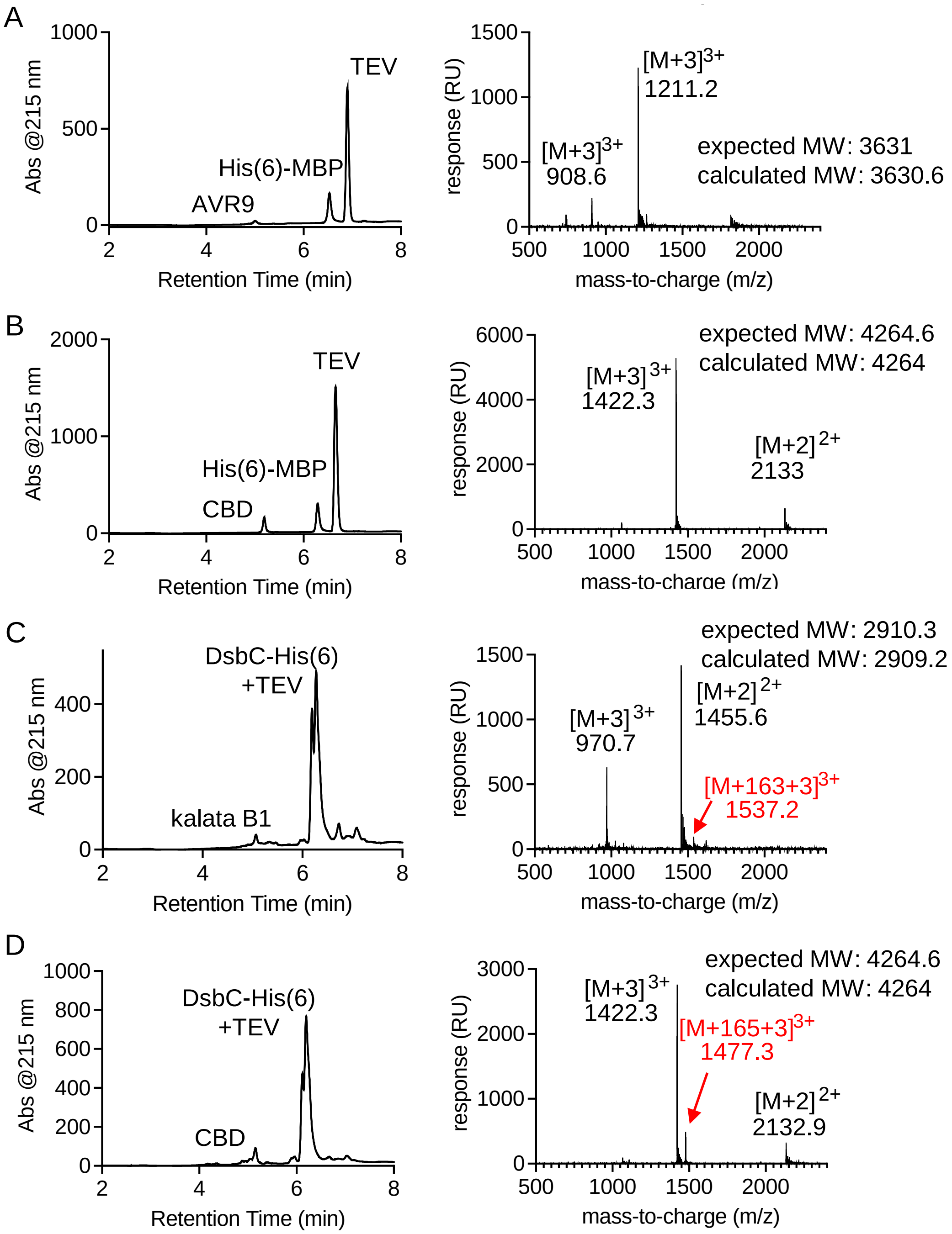

Figure S2. Examples of LC-MS analysis of pET vector expression of wild type DCPs with MBP or DsbC at 17 °C for 24 h. (A) His(6)-MBP-AVR9; (B) His(6)-MBP-CBD; (C) DcbC-His(6)-kalata B1, (D) DcbC-His(6)-CBD. For DcbC-His(6)-DCPs, a M+163 or M+165 Da modified impurity was identified in all samples (red arrow in C and D). All DCPs were purified as fusion protein then digested with TEV protease.

A

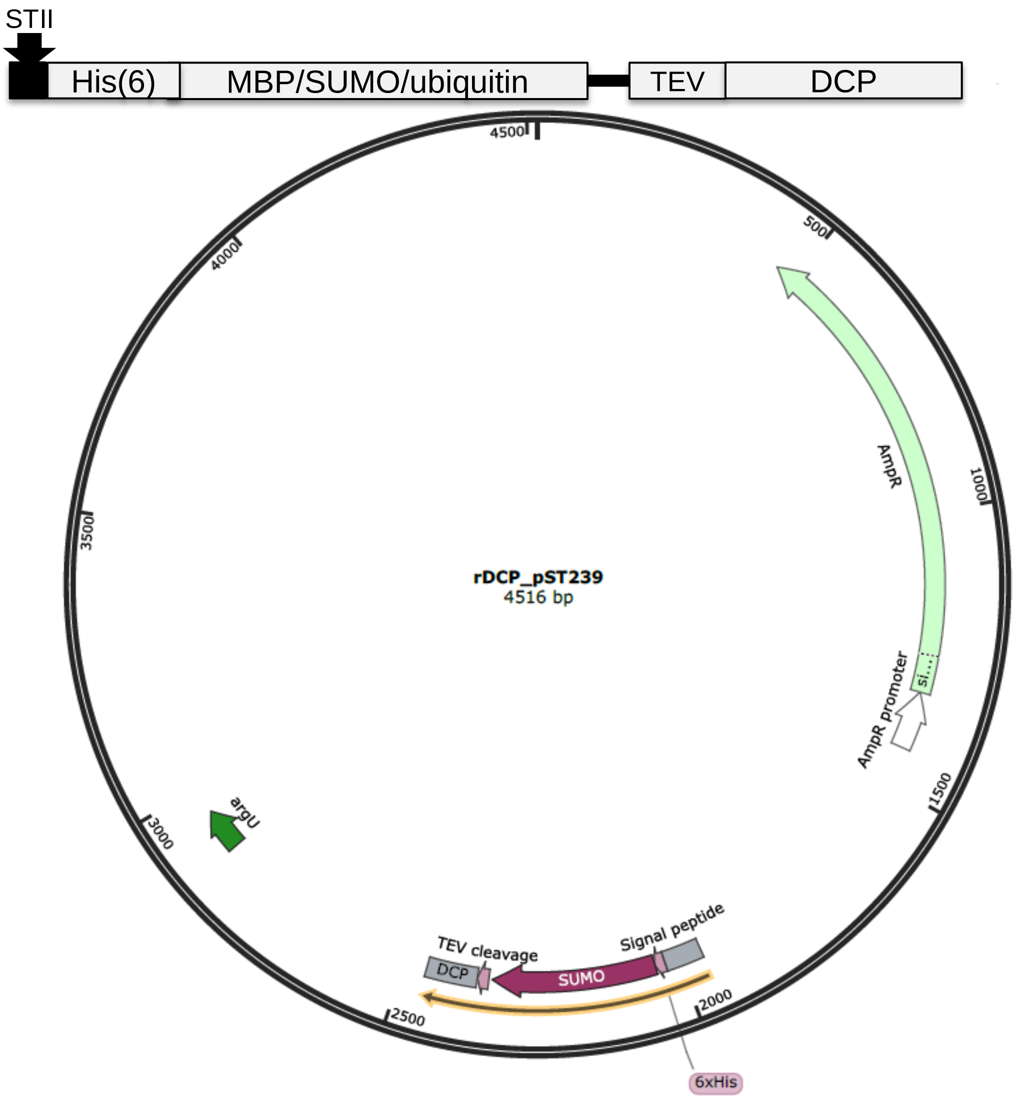

B

DCP	MBP (mg/L)	SUMO (mg/L)	Ubiquitin (mg/L)
	44H9 strain	44H9 strain	44H9 strain
kalata B1	>10	5-10	5-10
kalata thrombin	>10	>10	1-5
AVR9	0	>10	1-5
Circulin-A	>10	>10	1-5
conotoxin-MVIIA	>10	>10	1-5
Huwentoxin	1-5	>10	1-5
Min23	>10	>10	--
CBD	>10	5-10	>10
Charybdotoxin	1-5	1-5	1-5

- Single fully oxidized peptide
- Mixture of fully oxidized peptide isomers.
- Mixture of fully and partially oxidized peptide isomers
- DCP not isolated successfully

Figure S3. Summary of pST239 vector expression of wild type DCPs with MBP, SUMO or ubiquitin at 30 °C for 24 h. (A) Vector map of pST239. (B) All proteins were over-expressed in 44H9 cells and purified using Ni-IMAC tips on an automated liquid handler (Oasis). The folding profile (disulfide bond formation) was analyzed using LC-MS. Protein concentrations were calculated using their absorbance at 280 nm and individual extinction coefficient.

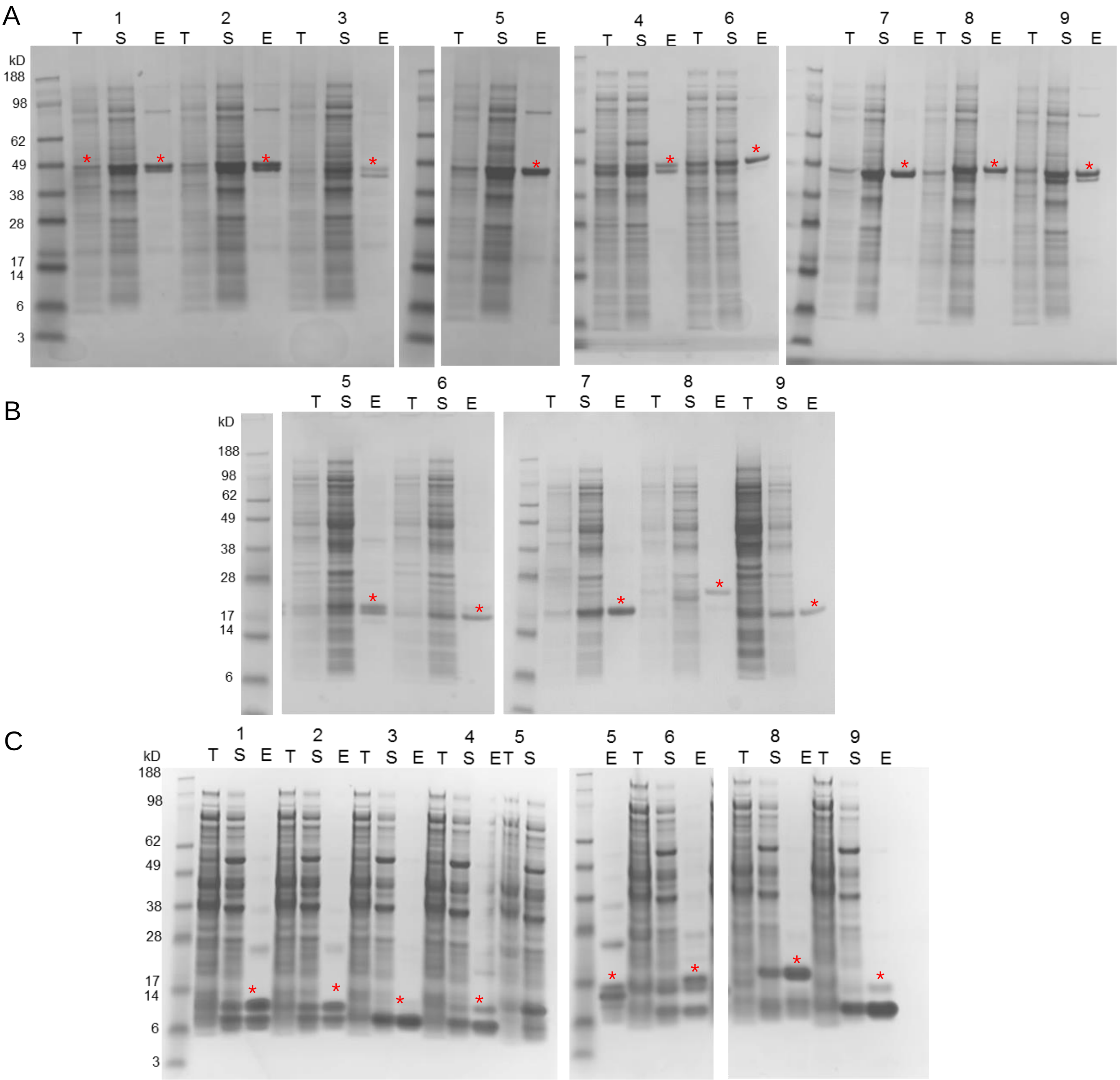

Figure S4. SDS-PAGE analysis of pST239 vector expression of wild type DCPs at 30 °C for 24 h. Bacterial expression vectors were generated containing ORF with His(6) tag, protein tag (A, MBP; B, SUMO; C, ubiquitin), and a TEV protease cutting site at the N-termini of DCPs. The fusion proteins were overexpressed and purified using Ni-IMAC tips on an automated liquid handler (Oasis). The expression profile of the fusion proteins was analyzed with SDS-PAGE. DCP1: kalata B1, DCP2: kalata thrombin binder, DCP3: AVR9, DCP4: Circulin A, DCP5: conotoxin-MVIIA, DCP6: Huwentoxin, DCP7: MIN23, DCP8: CBD, DCP9: Charybdotoxin. SDS-PAGE analysis of His(6)-SUMO-kalata B1/kalata thrombin binder/AVR9/Circulin A (sample 1-4 in panel B) is shown in Fig 1B. T: total lysate; S: soluble fraction; E: elute; *: purified protein.

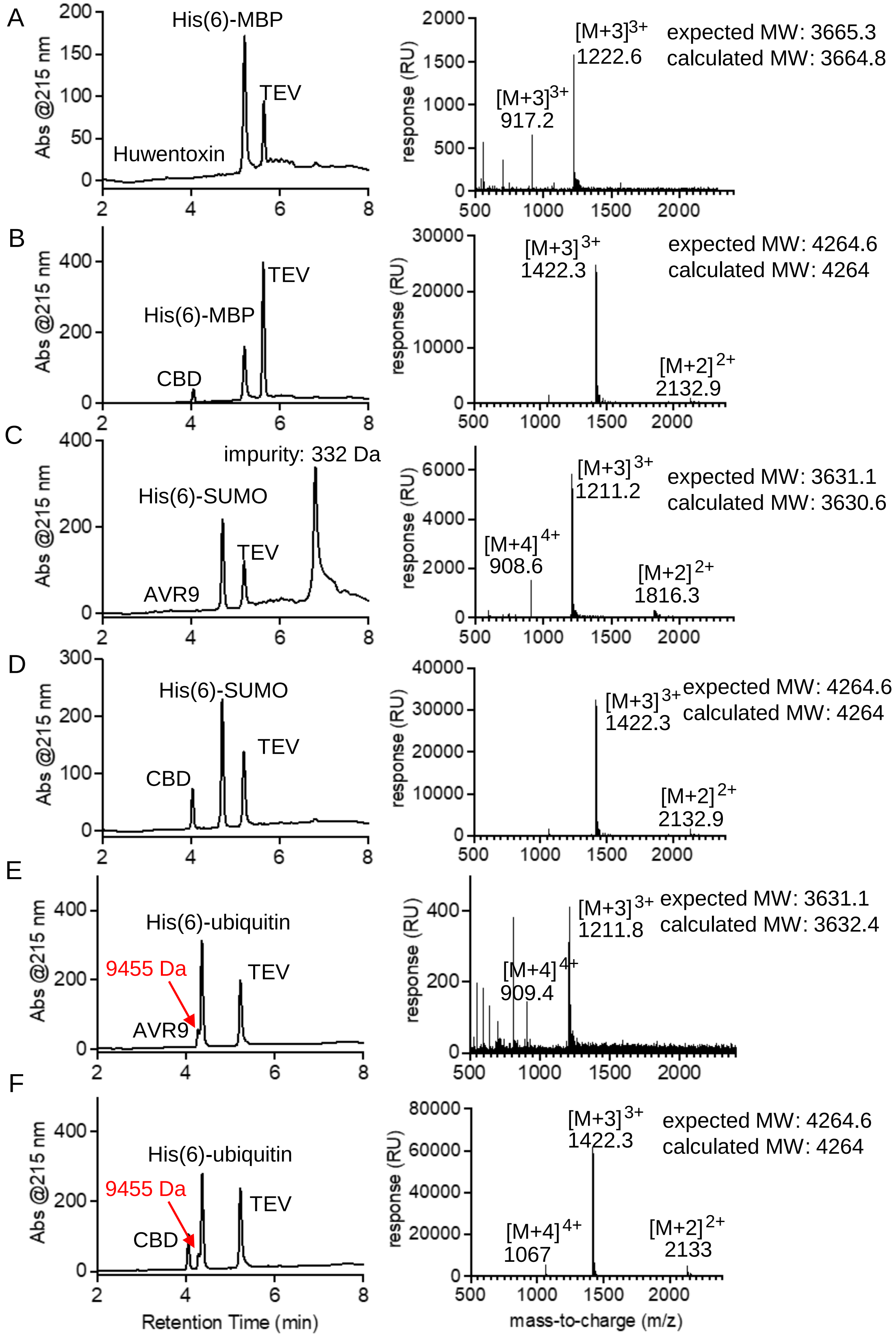

Figure S5. Examples of LC-MS analysis of pST239 vector expression of wild type DCPs at 30 °C for 24 h. (A) His(6)-MBP-Huwentoxin; (B) His(6)-MBP-CBD; (C) His(6)-SUMO-AVR9; (D) His(6)-SUMO-CBD; (E) His(6)-ubiquitin-AVR9; (F) His(6)-ubiquitin-CBD. For ubiquitin tagged DCPs, a 9455 Da truncation was identified in all samples (red arrow in E and F). All DCPs were purified as fusion protein then digested with TEV protease.

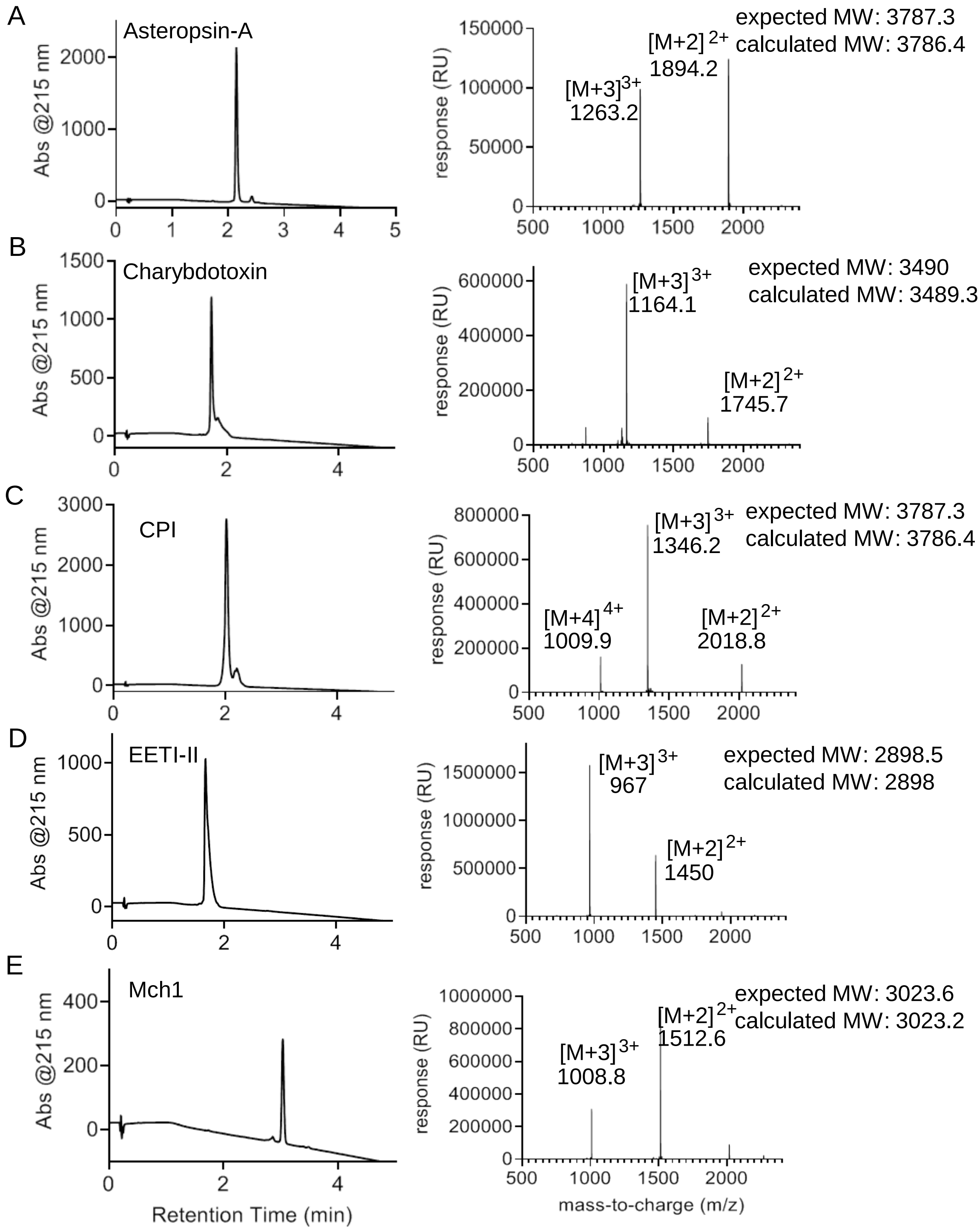

Figure S6. Examples of LC-MS analysis of synthetic WT DCPs. DCPs were folded in either 1) 0.1 M NH_4HCO_3, pH 9.0, 2 mM reduced glutathione (GSH), 0.5 mM oxidized glutathione (GSSG), 4 % DMSO, or 2) 0.1 M NH_4HCO_3, pH 9.0, 1 mM GSH, 50 % DMSO, at 0.5 mg/mL for 24 h at room temperature with shaking and purified through HPLC. A: Asteropsin-A; B: Charybdotoxin; C: CPI; D: EETI-II; E: Mch1.

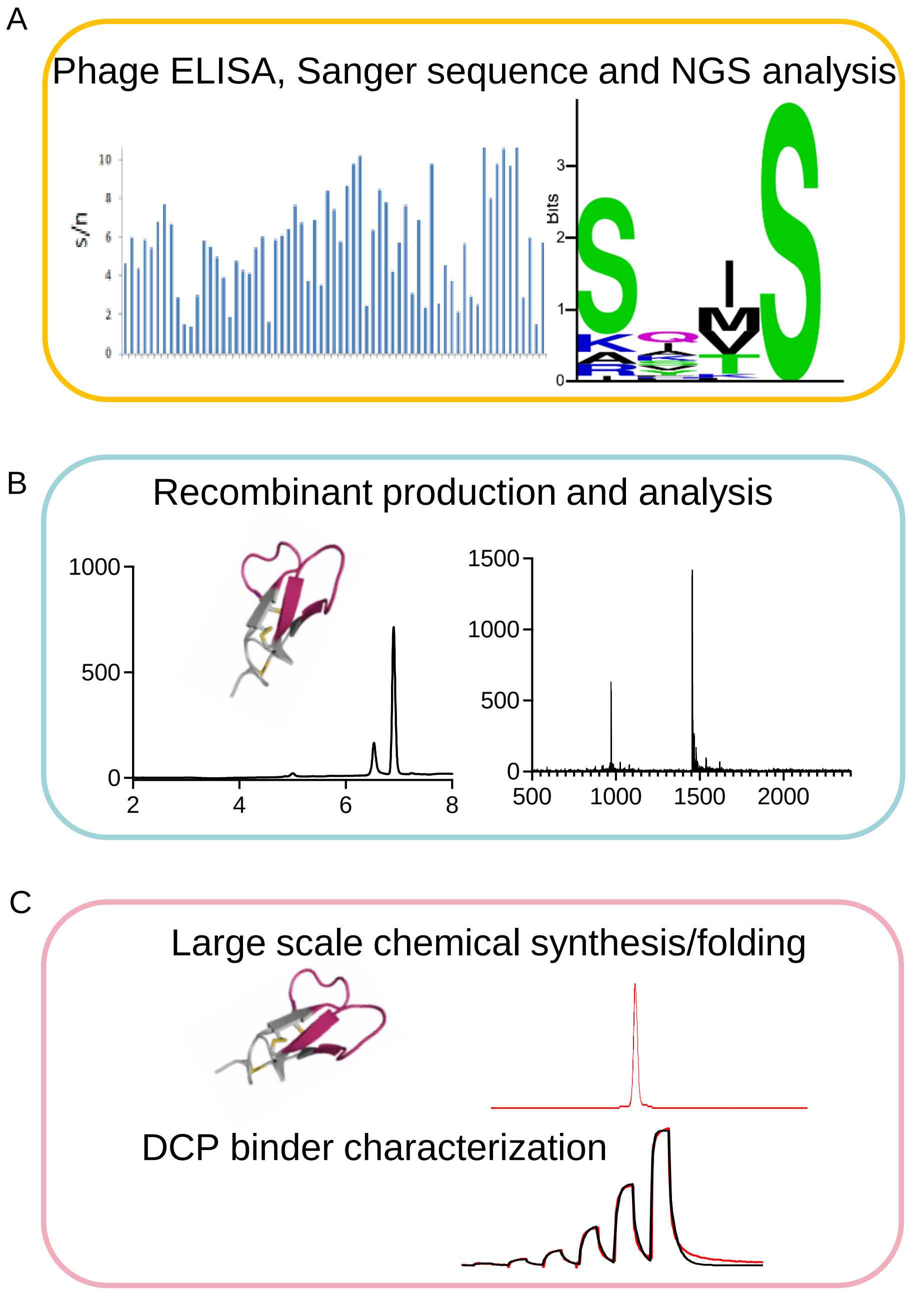

Figure S7. Schematic of streamlined DCP phage panning platform to facilitate identification, production and validation of DCP binders. A) Following selection with DCP libraries against the target, potential hits are screened using Sanger sequencing, NGS and phage spot ELISA. B) Oxidation (formation of disulfide bonds) of the hits are tested with the recombinant expression system. C) The best hits based on the two steps are chemically synthesized and oxidized, and subjected to further characterize with binding and functional assays.

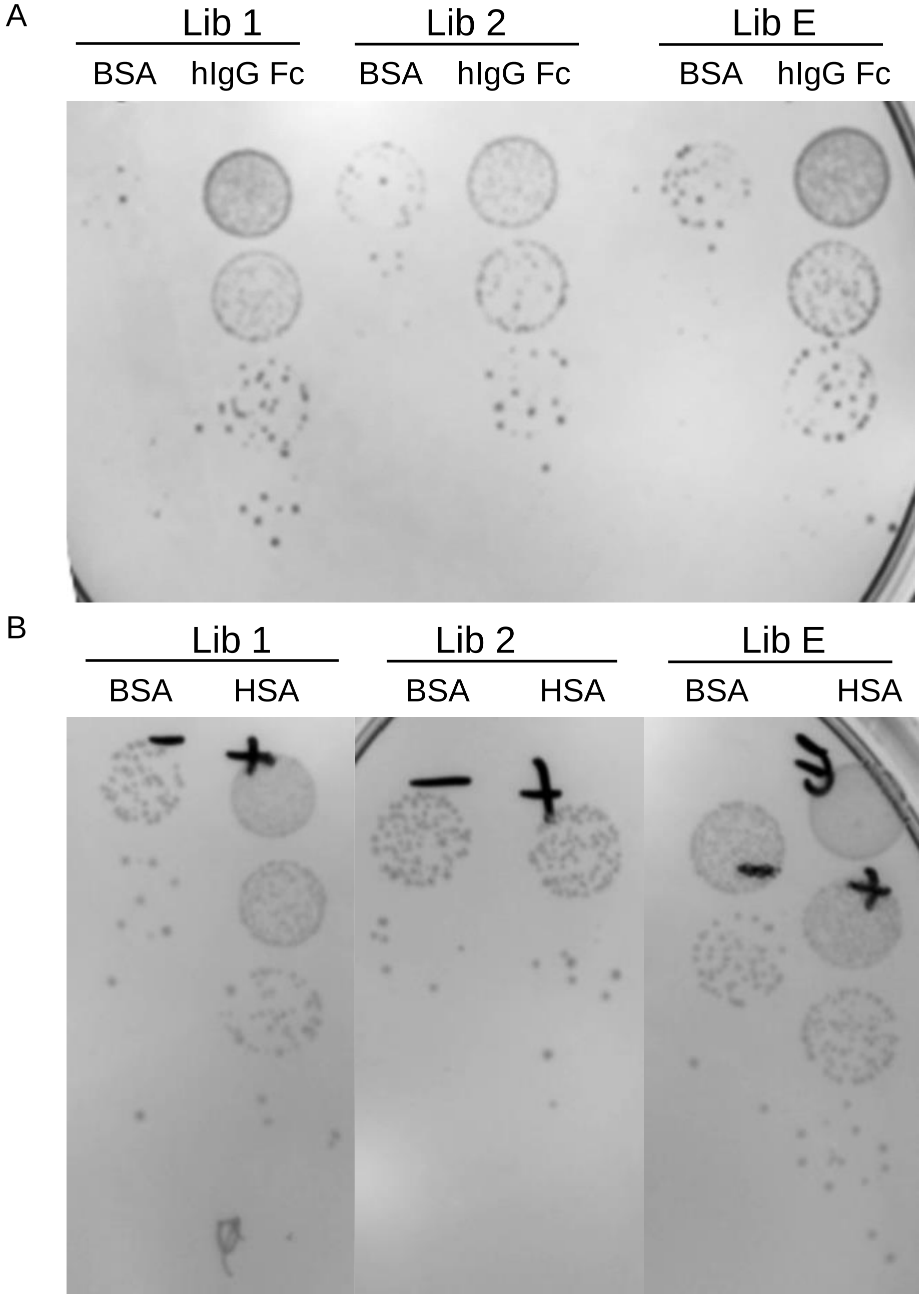

Figure S8. Selection of DCPs binding to human IgG Fc or human serum albumin. After four rounds of panning, binder enrichment against the two targets was reached for various DCP libraries. A) human IgG Fc: 1000 X, 30 X, 100 X for lib 1, 2, E; B) human serum albumin: 30 X, 0 X, 50 X for lib 1, 2, E. Fold of enrichment was calculated through dividing the number of binder phage colonies against the target by those against the control (BSA only). BSA, bovine serum albumin; HSA, human serum albumin. Refer to Table S1 for details of Lib 1, 2, and E.

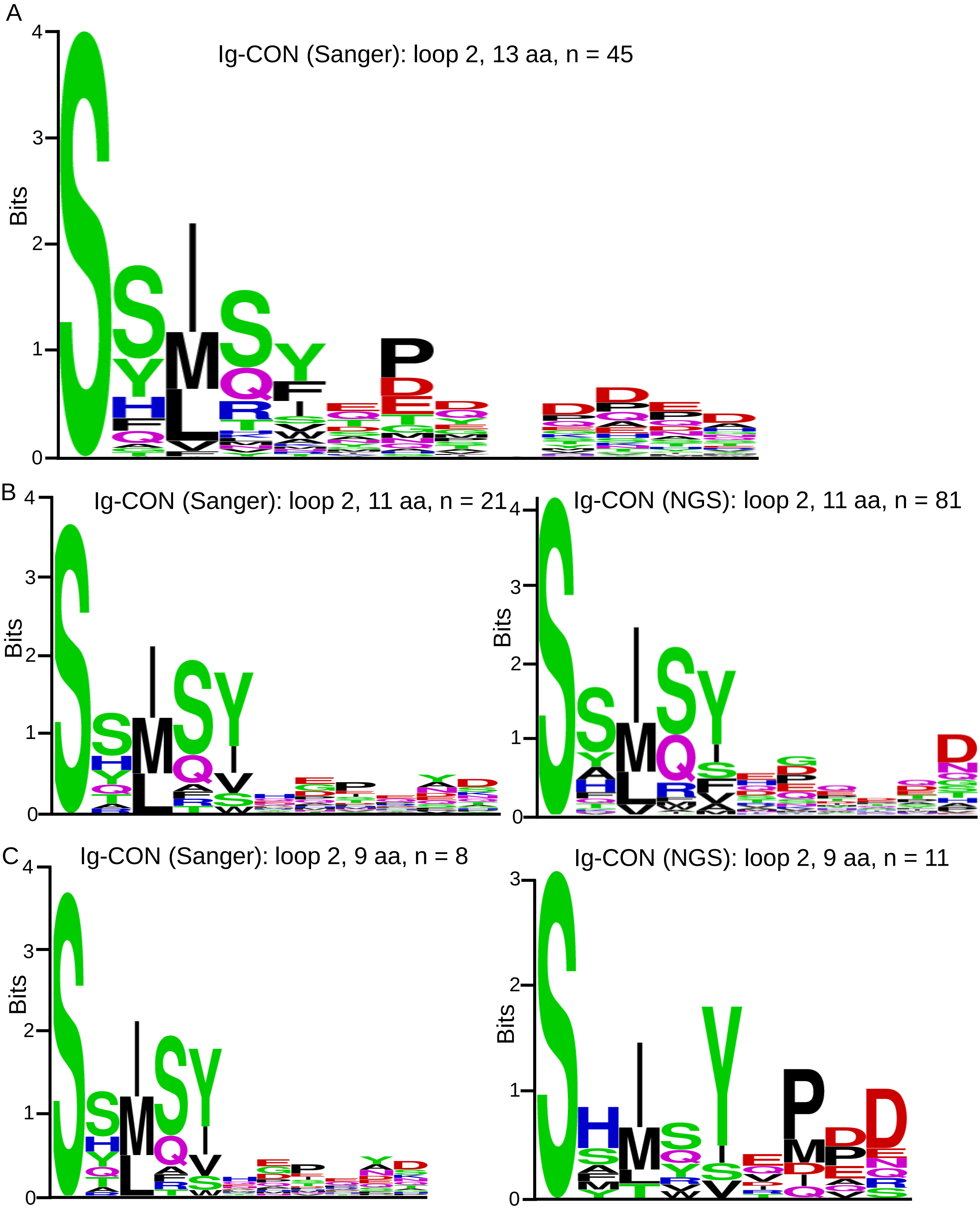

Figure S9. Sequence alignment for Ig-CON loop 2. Top hits with high S/N values were used. Data were analyzed by WebLogo. Ig-Con, DCPs against human IgG Fc identified from the conotoxin library.

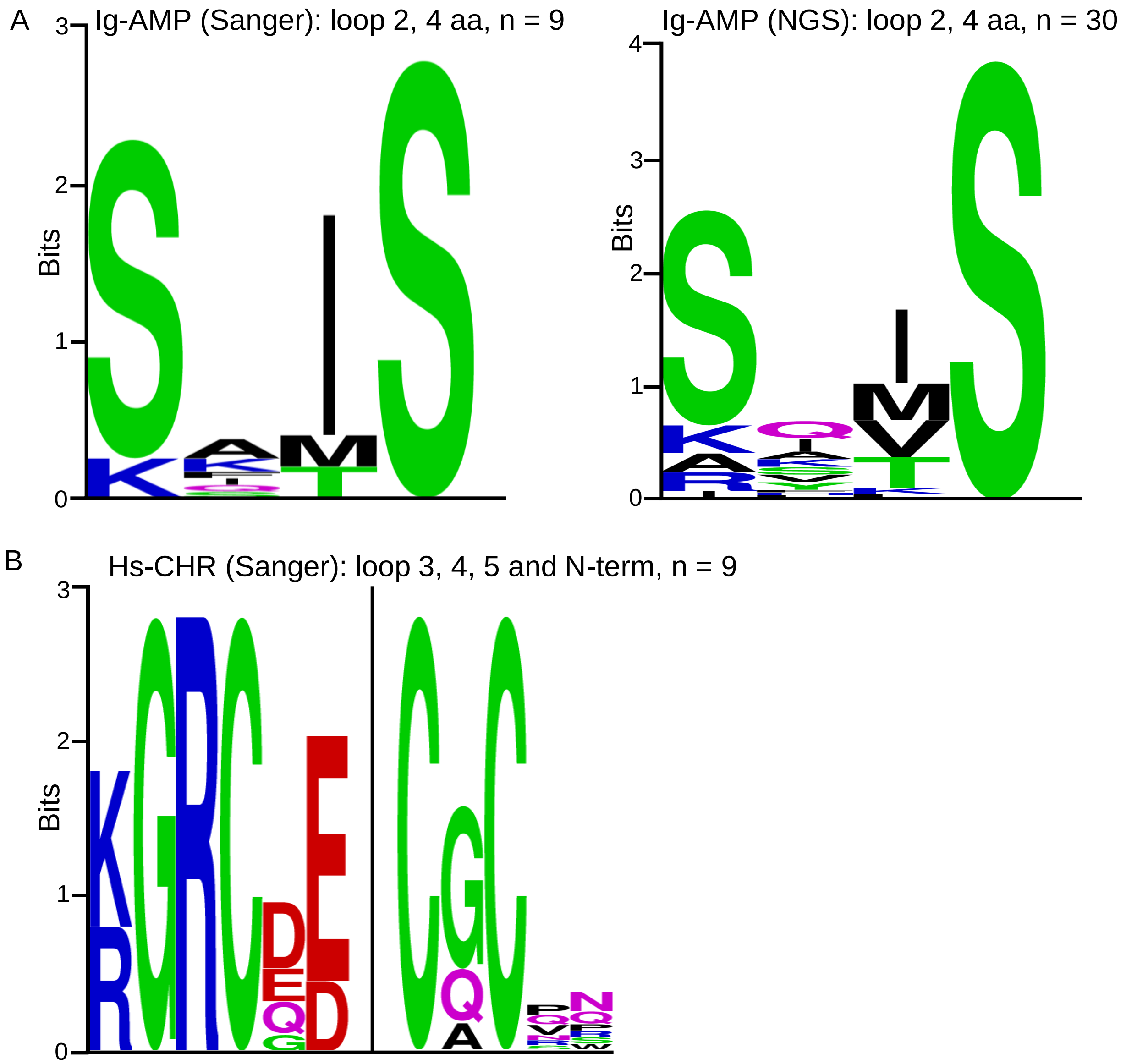

Figure S10. Sequence alignment for Ig- and Hs-DCPs. (A) Ig-AMP, loop 2. (B) Hs-CHR, loop 3, 4, 5 and N-term. Top hits with high S/N values were used. Data were analyzed by WebLogo. Ig-AMP, DCPs against human IgG Fc identified from AMP libraries. Hs-CHR, DCPs against human serum albumin identified from charybdotoxin libraries.

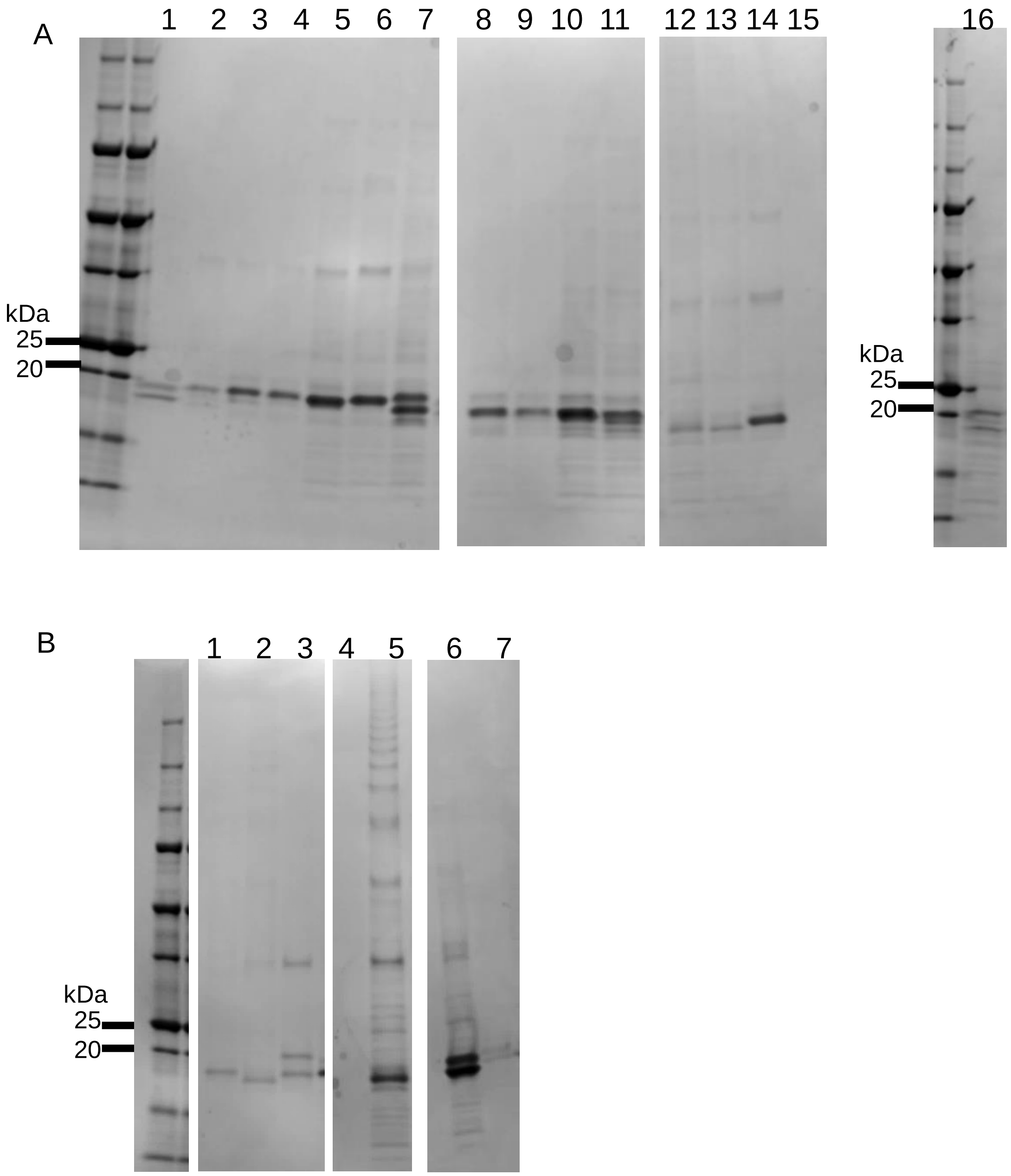

Figure S11. Recombinant expression of Ig and Hs-DCPs. Purified His(6)-SUMO-DCP proteins were analyzed using SDS-PAGE. (A) 1–7: Ig-CON-1–7; 8–11: Ig-AMP-1–4; 12-15: Ig-EET-1–4; 16: Ig-CPI-1. (B) 1–3: Hs-CHR-1-3; 4–5: Hs-EET-1, 2; 6–7: Hs-CON-n1, n2. Select cropped images are also shown in Fig 2. Ig-EET/Ig-CPI, DCPs against human IgG Fc identified from EETI-II/CPI libraries. Hs-EET/Hs-CON, DCPs against human serum albumin identified from EETI-II/conotoxin libraries.

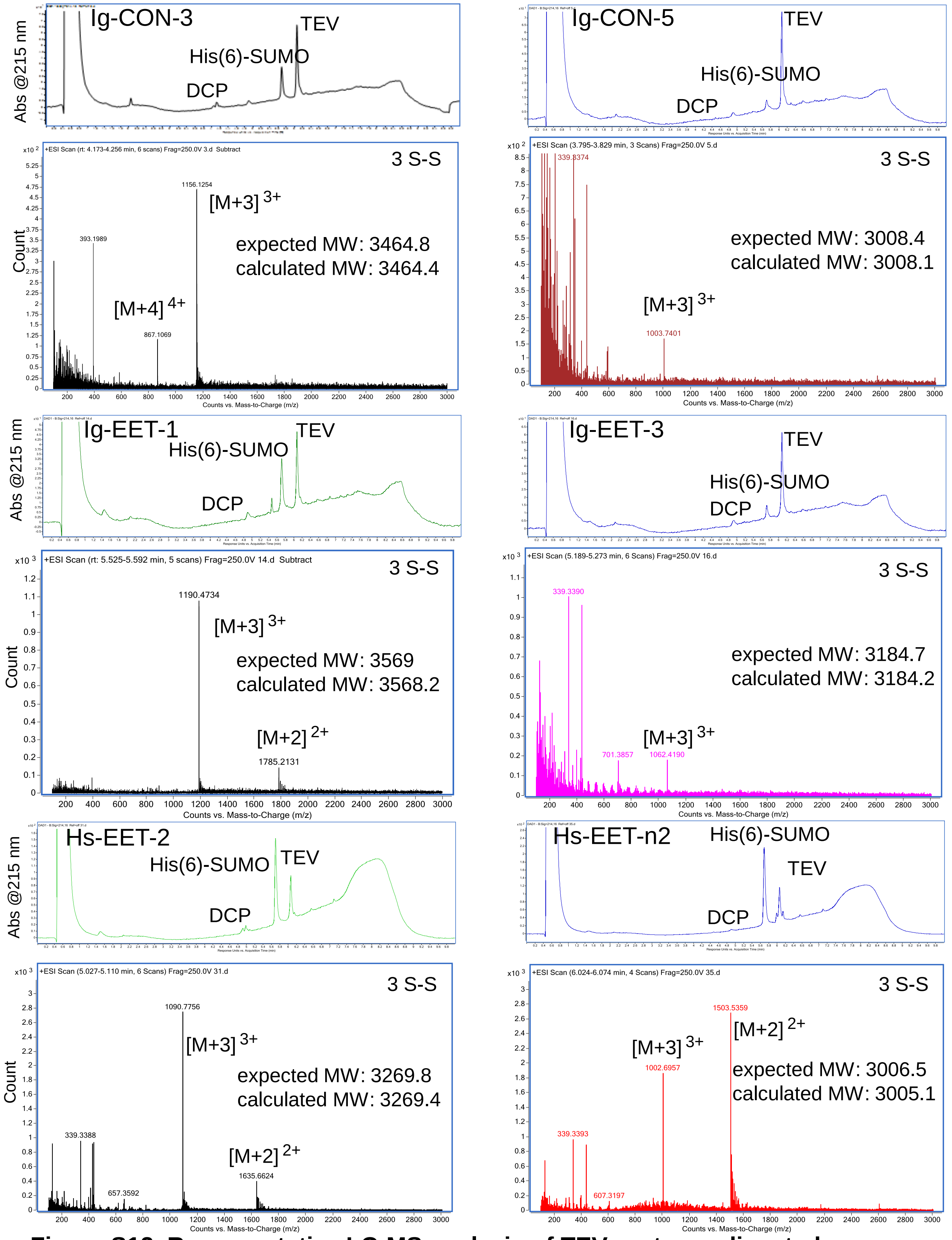

Figure S12. Representative LC-MS analysis of TEV protease digested recombinant Ig and Hs-DCPs. Purified His(6)-SUMO-DCP proteins were digested with TEV protease and analyzed using SDS-PAGE. MS analysis of the DCPs (labeled on the UV trace, upper panel) is shown in the lower panel.

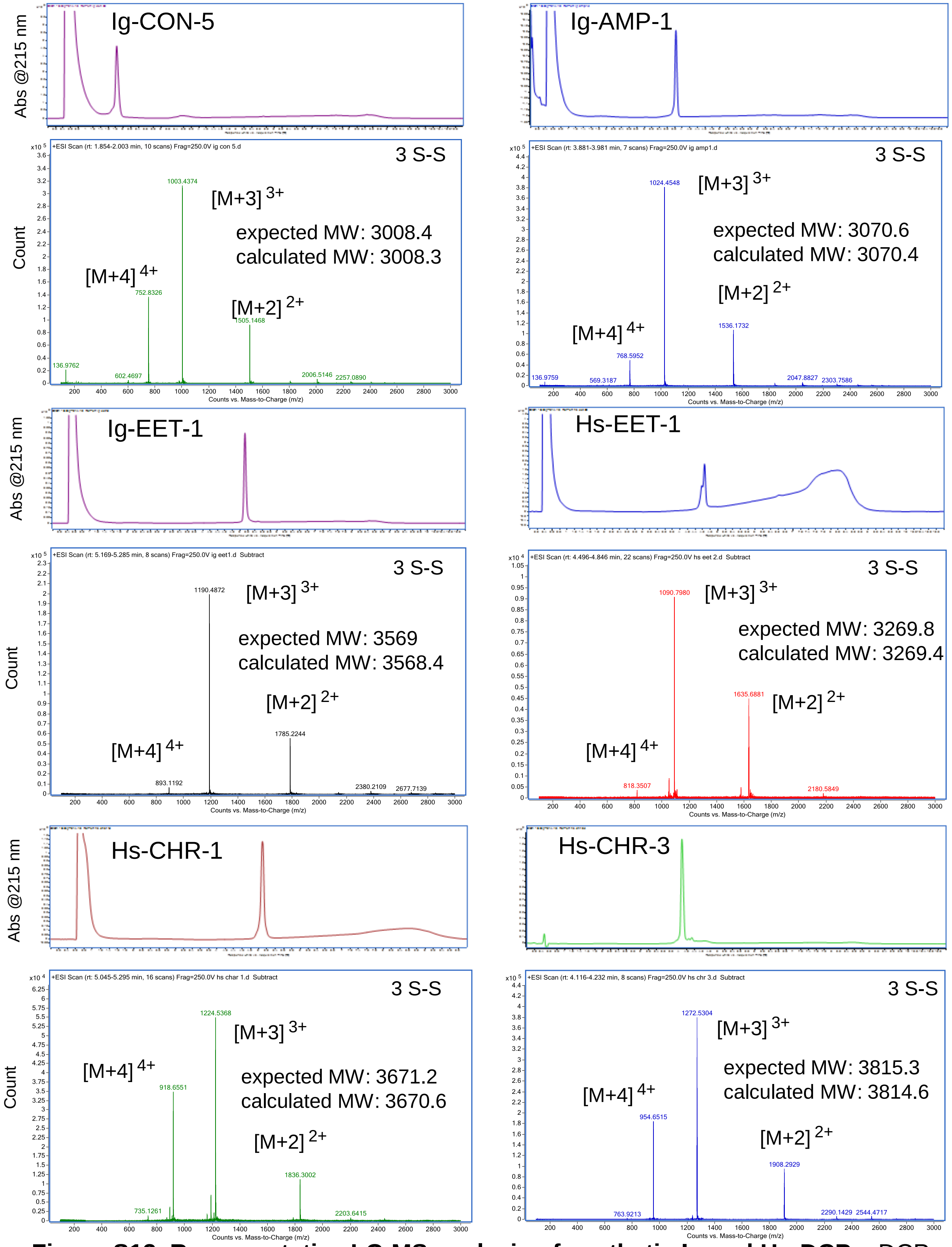

Figure S13. Representative LC-MS analysis of synthetic Ig and Hs-DCPs. DCPs were folded in the same buffer system as Figure S6 and purified through HPLC.

A

yield	K_D (His(6)-SUMO-DCP)	K_D (His(6)-SUMO+DCP)
17 mg/L	22.2 nM	10 nM

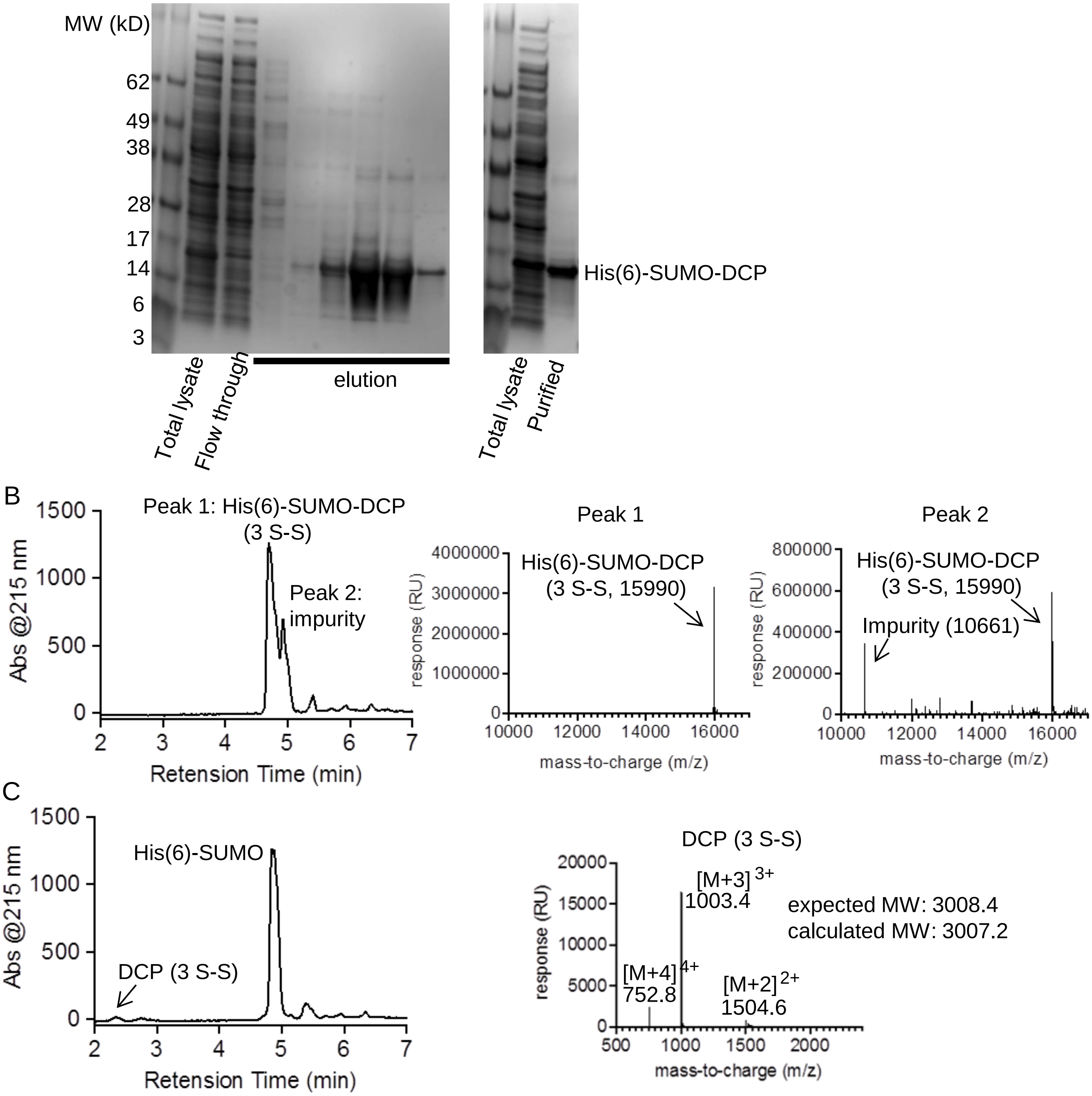

Figure S14. Recombinant Ig-CON-5 shows the same affinity as synthetic Ig-CON-5. The DCP-fusion protein was produced from 500 mL E. coli culture and purified using Ni-NTA resin. (A) Purified His(6)-SUMO-DCP was analyzed with SDS-PAGE. Binding affinity was measured using SPR. (B-C) LC-MS analysis of recombinant His(6)-SUMO-DCP (B) as well as TEV protease digested His(6)-SUMO+DCP (C) confirms formation of 3 disulfide bonds.

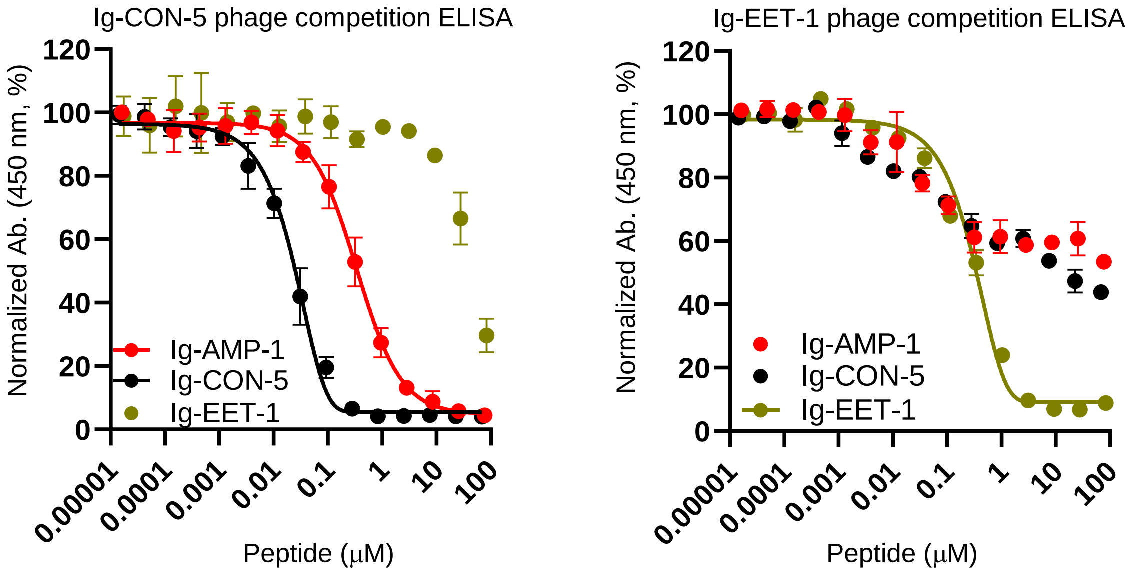

Figure S15. MOA studies of Ig- and Hs-DCPs. Phage competition ELISA indicates Ig-EET-1 binds to a different epitope from Ig-AMP-1 or Ig-CON-5. In this assay, phage displaying Ig-DCPs competes with various synthetic DCPs for binding to biotinylated hIgG Fc (immobilized on the plate). Error bar: standard deviation from duplicate samples. Representative data from at least three independent experiments are shown.

A

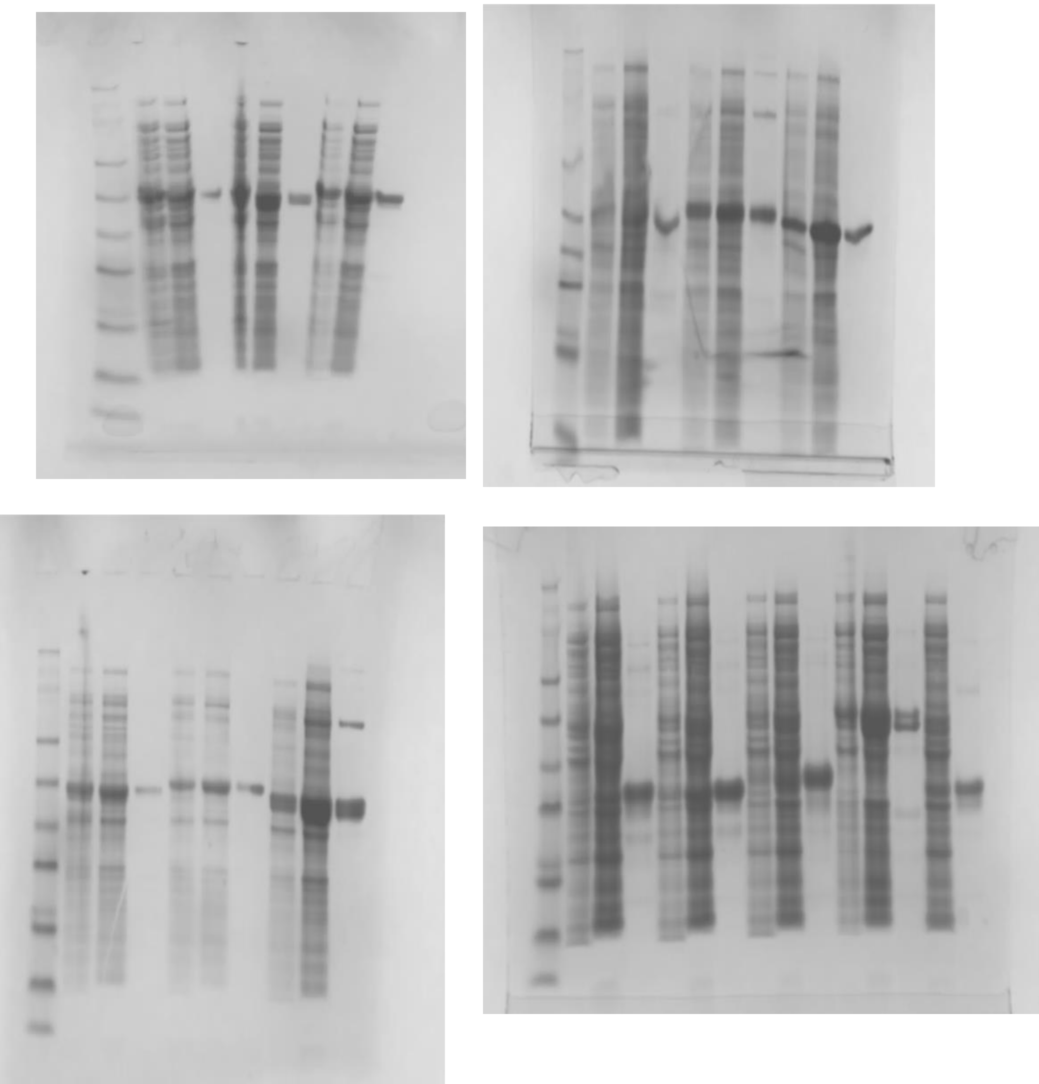

B

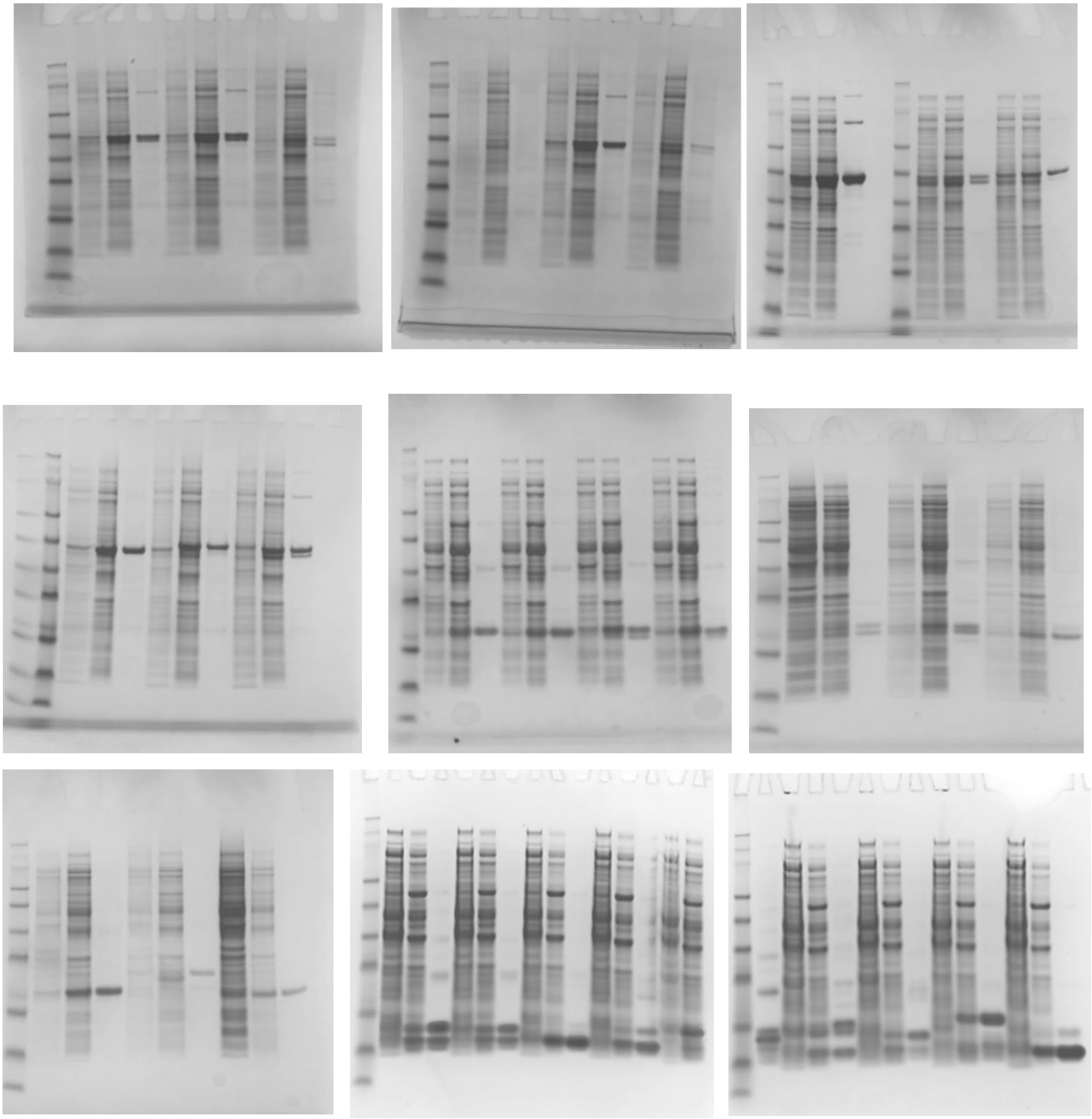

C

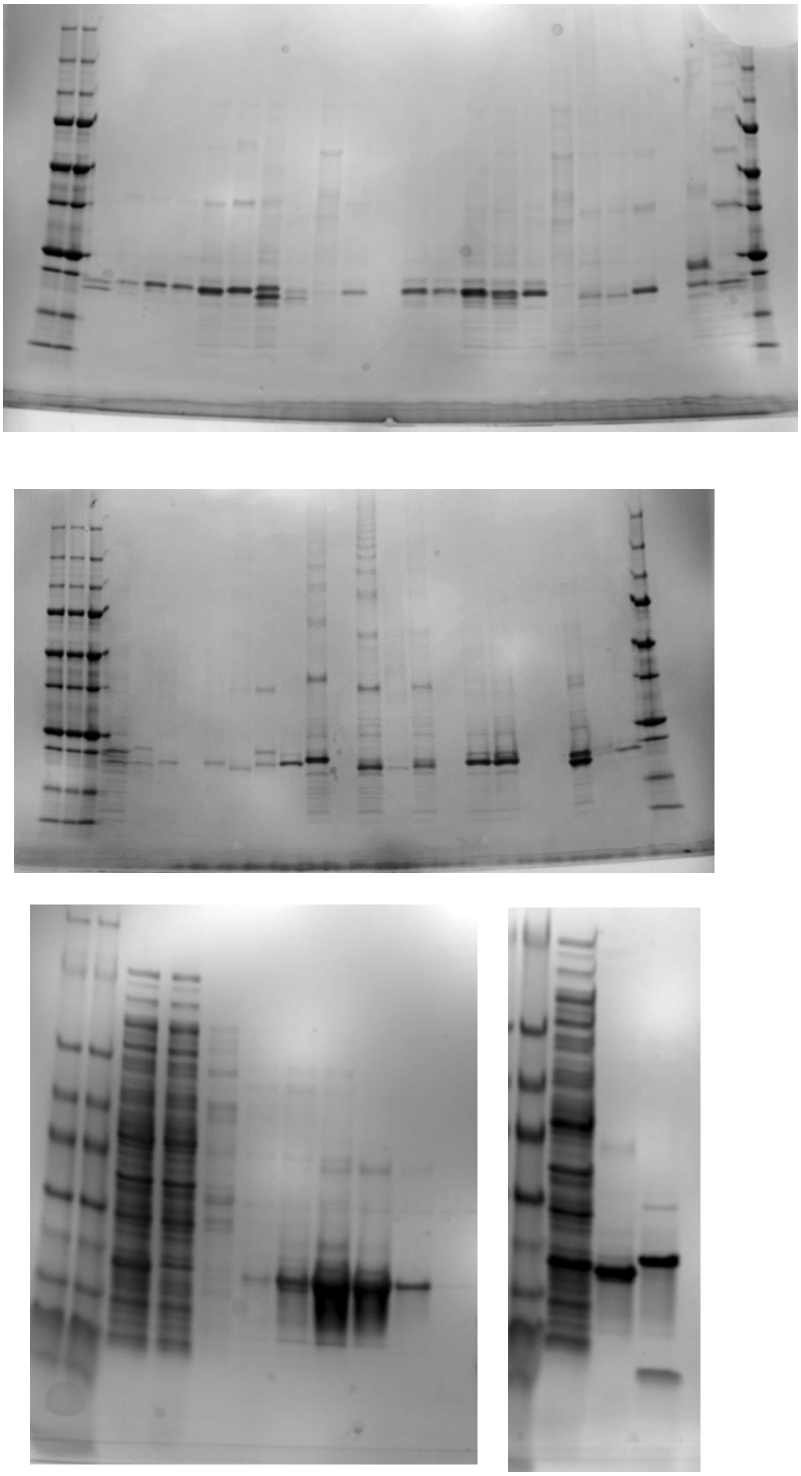

Figure S16. Full images of SDS-PAGE shown in Fig S1 (A), Fig 1B and S3 (B), Fig 2B, S10 and S14 (C).
