# Supplementary material for: A phage-displayed disulfide constrained peptide discovery platform yields novel human plasma protein binders
Source: PLoS One. 2024 Mar 28;19(3):e0299804. doi: 10.1371/journal.pone.0299804 (PMC10977726; doi:10.1371/journal.pone.0299804)
Supplement: S2 File — (PDF) [file pone.0299804.s002.pdf]

Table S1 . Construction of DCP phage libraries. 59 DCP phage libraries were generated based on 13 different DCP scaffolds. The residues highlighted in red were hard randomized or extended to create libraries derived from the native sequences. All Cys residues were kept to maintain the DCP scaffold. The libraries were pooled into three sets (lib E, 1 and 2) for phage panning.

group	scaffold	library sequences
Lib E	EETI-II	GC$X_6/X_8/X_{10}$CKQDSDCLAGCVCGPNGFCG
		GCPRILMRCKQDSDCLAGCVCX_5CG
		GC$X_6/X_8/X_{10}$CKQDSDCLAGCVC X_5CG
Lib 1	AVR9	AFDCLGQCGRCDFHKLQC$X_6/X_8/X_{10}/X_{12}/X_{14}/X_{16}$CNSSCTR
	Circulin-A	WIPCISAALGCSCKNKVCY$X_5/X_7/X_9/X_{11}/X_{13}$CGESCV
	Conotoxin-MVIIA	CKGKGAKCS$X_6/X_8/X_{10}/X_{12}$CCTGSCRSGKC
	Huwentoxin	ACKGVFDAC$X_6/X_8/X_{10}/X_{12}$ECCPNRVCSDKHKWCKWKL
	Charybdotoxin	VSCTTSKECWSVCQRLHNTSKGGCX_4CTCGP
		VSCTTSKECWSVCQRLHNTSXGXCX$_4$CXCXX
		VSCTX_3ECWXVCQX_2HNTSKGGCQGSFCTCGP
	CBD	GPTQSHYGQCGGXGYXGX_3CASGTTCQX_6SQCLPGAS
		GPTQSXXGXCGGIGYSGPTVCASGTTCQVLXPXXSXCLPGAS
	CBD amylose	GPTQSHYGQCGGXGYXGXXXCSGTTCQXXXXXXSQCLPGAS
		GPTQSXXGXCGGIGYVGGRYCSGTTCQHPXHXXSXCLPGAS
Lib 2	Mch1	GCAGKSCX_7CDAGCFCLPVGIVAGVCV
		GCAGKSCNILGSDPCDAGCFC$X_7/X_9/X_{11}$CV
		GCAGKSCX_7CDAGCFC$X_7/X_9/X_{11}$CV
	gurmarin	QCVKKDELCIPYYLDCCEPLEC$X_7/X_9/X_{11}$CIG
	Asteropsin-A	EGCAFEGESCNVQFYPCCX_6/X_8CIPGNPDGTCYYL
	AMP-1	VGEC$X_4/X_6/X_8$CPSGMCCSQFGYCGKGPKYCG
		VGECVRGRCX_4CCSQFGYCGKGPKYCG
		VGEC$X_4/X_6/X_8$CX_4CCSQFGYCGKGPKYCG
	CPI	HADPICNKPCX_5CSGAWFCQACWNSARTCGPYVG
		HADPICNKPCKTHDDCSGAWFCQAC$X_6/X_8/X_{10}$CGPYVG
		HADPICNKPCX_5CSGAWFCQAC$X_6/X_8/X_{10}$CGPYVG

Table S2. Oxidative folding conditions tested for synthetic linear peptides. Folding buffer with different buffering agent, organic solvent, redox agent, and pH value were used to test the optimal folding conditions for DCPs. Most DCPs can be folded in one of the two buffer systems: 1) 0.1 M NH_4HCO_3, pH 9.0, 2 mM reduced glutathione (GSH), 0.5 mM oxidized glutathione (GSSG), 4 % DMSO, or 2) 0.1 M NH_4HCO_3, pH 9.0, 1 mM

GSH, 50 % DMSO, at 0.5 mg/mL for 24 h at room temperature with shaking (highlighted in orange).

buffer	pH	redox agent	organic solvent
0.05 M NH_3Ac	8.5	2 mM GSH, 0.5 mM GSSG	10% DMSO
0.1 M NH_3Ac	8.5	2 mM GSSG	5% DMSO
0.1 M NH_3Ac	8.5	2 mM GSH, 0.5 mM GSSG	
0.1 M NH_3Ac	8.5	2 mM GSH, 0.5 mM GSSG	
0.1 M NH_3Ac	8.5	2 mM GSH, 0.5 mM GSSG	20% DMSO
0.1 M NH_3Ac	8.5	2 mM GSH, 0.5 mM GSSG	25% DMSO
0.1 M NH_3Ac	8.5	2 mM GSH, 0.5 mM GSSG	25% isopropanol
0.1 M NH_3Ac	8.5	2 mM GSH, 0.5 mM GSSG	5% DMSO
0.1 M NH_3Ac	8.5	2 mM GSH, 0.5 mM GSSG	50% acetic acid
0.1 M NH_3Ac	8.5	2 mM GSH, 0.5 mM GSSG	50% acetone
0.1 M NH_3Ac	8.5	2 mM GSH, 0.5 mM GSSG	50% ethanol
0.1 M NH_3Ac	8.5	2 mM GSH, 0.5 mM GSSG	50% isopropanol
0.1 M NH_3Ac	8.5	2 mM GSH, 0.5 mM GSSG	50% isopropanol
0.1 M NH_3Ac	8.5	2 mM GSH, 0.5 mM GSSG	50% methanol
0.1 M NH_3Ac	8.5	5 mM GSH	5% DMSO
0.1 M NH_3Ac	9	2 mM GSH, 0.5 mM GSSG	
0.1 M NH_3Ac	8.5	2 mM GSH, 0.5 mM GSSG	10% DMSO
0.1 M NH_4HCO_3	8		
0.05 M NH_4HCO_3	8.5	2 mM GSH, 0.5 mM GSSG	10% DMSO
0.1 M NH_4HCO_3	8.5	2 mM GSH, 0.5 mM GSSG	10% DMSO
0.1 M NH_4HCO_3	8	2 mM GSH, 0.5 mM GSSG	
0.1 M NH_4HCO_3	8	2 mM GSH, 0.5 mM GSSG	20% DMSO
0.1 M NH_4HCO_3	8.5	2 mM GSH, 0.5 mM GSSG	20% DMSO
0.1 M NH_4HCO_3	8.5	2 mM GSH, 0.5 mM GSSG	50% isopropanol
0.1 M NH_4HCO_3	9	2 mM GSH, 0.5 mM GSSG	4% DMSO
0.1 M NH_4HCO_3	9	2 mM GSH, 0.5 mM GSSG	20% DMSO
0.1 M NH_4HCO_3	8.5	2 mM GSH, 0.5 mM GSSG	
0.1 M NH_4HCO_3	8.5	2 mM GSH, 0.5 mM GSSG	10% DMSO
0.1 M NH_4HCO_3	8.5	2 mM GSH, 0.5 mM GSSG	
0.1 M NH_4HCO_3	8.5	2 mM GSH, 0.5 mM GSSG	10% DMSO
0.1 M NH_4HCO_3	9	1 mM GSH	50 % DMSO
water			

Table S3. Sanger sequencing and phage spot ELISA results of lib 1 panning against human IgG Fc. Single colonies (each representing a single phage population displaying multiple copies of one DCP variant) were manually picked and grown in 96 well plates for 20 h at 37 °C. Individual clones were sequenced and the phage

supernatant (1:3 in ELISA buffer) was used to test the binding to human IgG Fc in a phage spot ELISA assay. n: the number of clones; S: binding signal against the human IgG Fc; N: binding signal against the control (BSA). Top hits with high S/N values from each scaffold are shown.

conotoxin scaffold	S	S/N	n
CKGKGAKCSSISYSEMGDNQSCCTGSCRSGKC	1.50	12.45	1
CKGKGAKCSSMSVLDTHYDCCTGSCRSGKC	1.43	12.43	1
CKGKGAKCSSISMFDQDPQATCCTGSCRSGKC	1.43	11.84	1
CKGKGAKCSSMQYQDAFQESYCCTGSCRSGKC	1.30	11.25	1
CKGKGAKCSHLQYVMPQQTCCTGSCRSGKC	1.49	11.23	1
CKGKGAKCSQLSINDMYMQPGCCTGSCRSGKC	1.25	10.91	1
CKGKGAKCSAISSTMVMDDPHCCTGSCRSGKC	1.69	10.85	1
CKGKGAKCSSLSYHEIQSPCCTGSCRSGKC	1.51	10.77	1
CKGKGAKCSYISSQDINSSCCTGSCRSGKC	1.57	10.75	1
CKGKGAKCSSVSYAEDWGHQGCCTGSCRSGKC	1.44	10.75	1
CKGKGAKCSSMSFQAEVYDATCCTGSCRSGKC	1.37	10.70	1
CKGKGAKCSYISVQGQGADCCTGSCRSGKC	1.61	10.64	1
CKGKGAKCSSMSFEPSDDAPACCTGSCRSGKC	1.60	10.64	1
CKGKGAKCSSISVSTDFTASACCTGSCRSGKC	1.43	10.55	1
CKGKGAKCSSLRYEDTIAEEQCCTGSCRSGKC	1.28	10.51	1
CKGKGAKCSFIQNAPDPVPTDCCTGSCRSGKC	1.23	10.31	1
CKGKGAKCSSISISEHRASCCTGSCRSGKC	1.51	10.16	1
CKGKGAKCSYMSATDDMQHMNCCTGSCRSGKC	1.34	10.15	1
CKGKGAKCSHISIQHGDNKDVCCTGSCRSGKC	1.22	10.06	1
CKGKGAKCSSLQIGTPEPQPPCCTGSCRSGKC	1.32	10.04	1
CKGKGAKCSYIRYESAYTPYTCCTGSCRSGKC	1.22	9.93	1
CKGKGAKCSQMSYSMYNSDTVCCTGSCRSGKC	1.40	9.80	1
CKGKGAKCSYISVETQNQSPACCTGSCRSGKC	1.46	9.75	1
CKGKGAKCSYMRIEETSPPIDCCTGSCRSGKC	1.55	9.73	1
CKGKGAKCSSMSIHGDTNKCCTGSCRSGKC	1.33	9.73	1
CKGKGAKCSSIQYHEPFANCCTGSCRSGKC	1.17	9.69	1
CKGKGAKCSYLSANGYIKSNYCCTGSCRSGKC	1.33	9.65	1
CKGKGAKCSSMSFNQTQDEAHCCTGSCRSGKC	1.27	9.55	1
CKGKGAKCSGIMYMPQQYPYDCCTGSCRSGKC	1.43	9.49	1
CKGKGAKCSSLSYTGPVYYCCTGSCRSGKC	1.23	9.48	1
CKGKGAKCSYLQYAEDTKQESCCTGSCRSGKC	1.43	9.46	1
CKGKGAKCSSVRYEPIIEHQHCCTGSCRSGKC	1.36	9.41	1
CKGKGAKCSYLVREDQRGAQDCCTGSCRSGKC	1.08	9.22	1
CKGKGAKCSSLRFQGQEDKEQCCTGSCRSGKC	1.20	9.21	1
CKGKGAKCSSISSYPQTYDERCCTGSCRSGKC	1.50	9.18	1

CKGKGAKCSFIQWTPSYQDPQCCTGSCRSGKC	1.51	9.16	1
CKGKGAKCSYMSINAPAEDCCTGSCRSGKC	1.34	9.11	1
CKGKGAKCSAISVYQMWDTCCTGSCRSGKC	1.40	9.08	1
CKGKGAKCSSMTYGGGQPQTDCCTGSCRSGKC	1.61	8.97	1
CKGKGAKCSHLRSEFFPQDCCTGSCRSGKC	1.31	8.92	1
CKGKGAKCSTISYDGSRDNCCTGSCRSGKC	1.20	8.91	1
CKGKGAKCSSIQWHNPEPDCCTGSCRSGKC	1.22	8.79	1
CKGKGAKCSSIQFMPESHQDACCTGSCRSGKC	1.43	8.78	1
CKGKGAKCSSISTYNFQDYNNCCTGSCRSGKC	1.26	8.69	1
CKGKGAKCSHMSFQPQRSAPDCCTGSCRSGKC	1.28	8.58	1
CKGKGAKCSSMFYEPEEFSCCTGSCRSGKC	1.50	8.51	1
CKGKGAKCSSITSSEYQVDNRCCTGSCRSGKC	1.25	8.39	1
CKGKGAKCSQIKYDPEPDGEHCCTGSCRSGKC	1.55	8.38	1
CKGKGAKCSYMRFEPMDTTHCCTGSCRSGKC	1.43	8.36	1
CKGKGAKCSAIQYNESTCCTGSCRSGKC	1.27	8.34	1
CKGKGAKCSFIHWTPDVQVEECCTGSCRSGKC	1.02	8.31	1
CKGKGAKCSTITYQTPTPENSCCTGSCRSGKC	1.19	8.30	1
CKGKGAKCSQMSYNTSQEACCTGSCRSGKC	1.18	8.26	1
CKGKGAKCSSINYTPYEEDHDCCTGSCRSGKC	1.24	8.20	1
CKGKGAKCSQMSYMEPEVQCCTGSCRSGKC	1.54	8.01	1
CKGKGAKCSHITYQQYQYQCCTGSCRSGKC	1.33	7.99	1
CKGKGAKCSSLQVDPYWDDQGCCTGSCRSGKC	1.17	7.74	1
CKGKGAKCSSIAYRHEPNKCCTGSCRSGKC	1.36	7.71	1
CKGKGAKCSSIQMDEGDCCTGSCRSGKC	1.10	7.66	1
CKGKGAKCSHLQWTPVEEPSECCTGSCRSGKC	1.55	7.66	1
AMP-1 scaffold	S	S/N	n
VGECVRGRCSSISCCSQFGYCGKGPKYCG	1.17	6.88	1
VGECVRGRCSAISCCSQFGYCGKGPKYCG	1.12	5.77	1
VGECVRGRCSKISCCSQFGYCGKGPKYCG	0.77	5.75	1
VGECVRGRCSQISCCSQFGYCGKGPKYCG	0.61	4.45	1
VGECVRGRCKITSCCSQFGYCGKGPKYCG	0.67	4.19	1
VGECVRGRCSKISCCSQFGYCGKGPKYCG	0.78	3.93	1
VGECVRGRCSFISCCSQFGYCGKGPKYCG	0.51	3.10	1
VGECVRGRCSAISCCSQFGYCGKGPKYCG	0.59	2.98	1
VGECVRGRCSAMSCCSQFGYCGKGPKYCG	0.26	1.07	1
EETI-II scaffold	S	S/N	n
GCGFWYHKWFCKQDSDCLAGCVCSKTGCG	1.47	8.77	3
GCVHGYWYRQWLCKQDSDCLAGCVCQPAQ	1.38	7.27	3
GCGYWYMQWFCKQDSDCLAGCVCRPKPEC	1.39	6.88	10
GCHASYWYQKWFCKQDSDCLAGCVCWQEN	1.35	6.51	8

GCGFWYMQWFCKQDSDCLAGCVCDPMGNC	0.63	4.87	1
GCQAHYWYRVWFCKQDSDCLAGCVCGPNG	0.95	4.72	1
GCHMGYWYMMWFCKQDSDCLAGCVCAPKP	1.01	4.13	1
GCDQGYWYHYWFCKQDSDCLAGCVCGPNG	0.51	2.98	1

Table S4. Sanger sequencing and phage spot ELISA results of panning against human serum albumin. Single colonies (each representing a single phage population displaying multiple copies of one DCP variant) were manually picked and grown in 96 well plates for 20 h at 37 °C. Individual clones were sequenced and the phage supernatant (1:3 in ELISA buffer) was used to test the binding to human serum albumin in a phage spot ELISA assay. n: the number of clones; S: binding signal against the human serum albumin; N: binding signal against the control (BSA). Top hits with high S/N values from each scaffold are shown.

Charybdotoxin scaffold	S	S/N	n
VSCTTSKECWSVCQRLHNTSYGTGCMRNTCYCTP	0.89	15.44	2
VSCTTSKECWSVCQRLHNTSRGRCDEICGCVN	0.79	11.45	2
VSCTTSKECWSVCQRLHNTSKGRCDEICQCNS	0.52	10.90	1
VSCTTSKECWSVCQRLHNTSRGRCGEICACQP	0.57	9.41	2
VSCTTSKECWSVCQRLHNTSRGRCEEICGCSN	0.57	8.42	3
VSCTTSKECWSVCQRLHNTSKGRCDEICGCRW	1.08	7.28	1
VSCTTSKECWSVCQRLHNTSKGRCEEICQCQR	0.27	5.05	1
VSCTTSKECWSVCQRLHNTSKGRCQDICGCPQ	0.22	4.55	1
EETI-II scaffold	S	S/N	n
GCLYKKWHAACKQDSDCLAGCVCYFSQSCG	0.68	5.47	1
GCLYKKWHADCKQDSDCLAGCVCHFSQSCG	0.64	5.95	1
GCLYRKWHNQCKQDSDCLAGCVCHWYQSCG	0.59	6.20	1

Table S5. NGS results for lib 1 panning against human IgG Fc. Top sequences are shown. S, number of the sequences shown in selection against the target protein; N, number of the sequences shown in selection against BSA + 1.

sequence	S	N	S/N
CKGKGAKCSTISYDGSRDNCCTGSCRSGKC	52	1	52
CKGKGAKCSSVQADDYGYSLFTGSCRSGKC	51	1	51
CKGKGAKCSSMSYEPVDCCTGSCRSGKC	37	1	37
CKGKGAKCSSIQWHNPEPDCCTGSCRSGKC	55	2	28
CKGKGAKCSAISSTIQKQDCCTGSCRSGKC	27	1	27
CKGKGAKCSSMSSRWMDMDCCTGSCRSGKC	26	1	26
CKGKGAKCSSLSYESTPQDCCTGSCRSGKC	209	9	23
CKGKGAKCSKLFYTPPDVVGSCHSGKC	41	2	21
CKGKGAKCSYMIYEEPIQHCCTGSCRSGKC	56	3	19

CKGKGAKCSSLQYQETRNDCCTGSCRSGKC	65	4	16
CKGKGAKCSHLRSEFFPQDCCTGSCRSGKC	134	9	15
CKGKGAKCSQLSIRSENQDCCTGSCRSGKC	27	2	14
CKGKGAKCSSMSYAKQQQDCCTGSCRSGKC	37	3	12
CKGKGAKCSVISYSPVQQGCCTGSCRSGKC	119	11	11
CKGKGAKCSAIRYQQQDDTCCTGSCRSGKC	37	4	9.3
CKGKGAKCSSLSYHEIQSPCCTGSCRSGKC	37	5	7.4
CKGKGAKCSSVSIHAIDTNCCTGSCRSGKC	29	4	7.3
CKGKGAKCSTIQFRPQAPDCCTGSCRSGKC	41	7	5.9
CKGKGAKCSQIFYSQQTANCCTGSCRSGKC	62	11	5.6
CKGKGAKCSSMWYDGGQPDCCTGSCRSGKC	27	6	4.5
CKGKGAKCSTISSHMVDHDCCTGSCRSGKC	64	15	4.3
CKGKGAKCSSIRFDELPENCCTGSCRSGKC	31	8	3.9
CKGKGAKCSSIQYHEPFANCCTGSCRSGKC	53	15	3.5
CKGKGAKCSSISYNSTTTTCCTGSCRSGKC	32	11	2.9
CKGKGAKCSHIMFKMEDPHCCTGSCRSGKC	68	24	2.8
CKGKGAKCSFISSMQMEEDCCTGSCRSGKC	37	16	2.3
CKGKGAKCSSMSVLDTHYDCCTGSCRSGKC	38	17	2.2
CKGKGAKCSHTWYEPANCCTGSCRSGKC	88	44	2
CKGKGAKCSSISINQETTDCCTGSCRSGKC	119	97	1.2
CKGKGAKCSSIQYIPDAMDCCTGSCRSGKC	36	41	0.9

Table S6. NGS results for lib 2 panning against human IgG Fc. Top sequences are shown. S, number of the sequences shown in selection against the target protein; N, number of the sequences shown in selection against BSA + 1.

sequence	S	N	S/N
CKGKGAKCSQITIKDYSNQCCTGSCRSGKC	138	1	138
VGECVRGRCSAISCCSQFGYCGKGPKYCG	118	1	118
CKGKGAKCSQISAEVNDSCCTGSCRSGKC	111	1	111
VGECVRGRCSKISCCSQFGYCGKGPKYCG	86	1	86
CKGKGAKCSYMIYEEPIQHCCTGSCRSGKC	517	7	74
CKGKGAKCSEIHYFMEDPQCCTGSCRSGKC	64	1	64
VGECVRGRCSYISCCSQFGYCGKGPKYCG	53	1	53
VGECVRGRCSQISCCSQFGYCGKGPKYCG	158	3	53
VGECVRGRCSQVSCCSQFGYCGKGPKYCG	48	1	48
VGECVRGRCSSISCCSQFGYCGKGPKYCG	42	1	42
VGECVRGRCSFISCCSQFGYCGKGPKYCG	32	1	32
CKGKGAKCSYLSKFQETCCTGSCRSGKC	28	1	28
VGECVRGRCKIVSCCSQFGYCGKGPKYCG	19	1	19
CKGKGAKCSHISHSTQPCCTGSCRSGKC	17	1	17

VGECVRGRCSAMSCCSQFGYCGKGPKYCG	13	1	13
VGECVRGRCKITSCCSQFGYCGKGPKYCG	13	1	13
VGECVRGRCSSVSCCSQFGYCGKGPKYCG	13	1	13
VGECVRGRCSSMSCCSQFGYCGKGPKYCG	22	2	11
VGECVRGRCRITSCCSQFGYCGKGPKYCG	11	1	11
VGECVRGRCIIKSCCSQFGYCGKGPKYCG	10	1	10
VGECVRGRCSKMSCCSQFGYCGKGPKYCG	10	1	10
VGECVRGRCSQMSCCSQFGYCGKGPKYCG	29	3	9.7
VGECVRGRCSRISCCSQFGYCGKGPKYCG	9	1	9
VGECVRGRCSHISCCSQFGYCGKGPKYCG	8	1	8
CKGKGAKCSTIQFFDCCTSSCRSGKC	8	1	8
CKGKGAKCSHIMNWDMACCTGSCRSGKC	7	1	7
VGECVRGRCSVISCCSQFGYCGKGPKYCG	6	1	6
VGECVRGRCSQTSCCSQFGYCGKGPKYCG	5	1	5
CKGKGAKCSYLYFDPGVCCTGSCRSGKC	5	1	5
CKGKGAKCSFLWSNDCCTGSCRSGKC	5	1	5
VGECVRGRCSYVSCCSQFGYCGKGPKYCG	4	1	4
CKGKGAKCSHMWDQEGDCCTGSCRSGKC	3	1	3
VGECVRGRCAQMSCCSQFGYCGKGPKYCG	2	1	2

Table S7. NGS results for lib E panning against human IgG Fc. Top sequences are shown. S, number of the sequences shown in selection against the target protein; N, number of the sequences shown in selection against BSA + 1.

sequence	S	N	S/N
GCYWAAQWAYHMCKQDSDCLAGWGRCG	86	1	86
GCGFWYHKWFCKQDSDCLAGCVCSKTGCG	79	1	79
GCYWYARWFCKQDSDCLAGCVCMNRQHCG	28	1	28
VVSRITSTLSVKDSDCLAGCVCGPNGFCG	16	1	16
GCYWISVWCKQDSDCLADCG	8	1	8
GCNTEKFKCKQDSVCSGIRACG	6	1	6
GCPRILMRCKQDSDCLAGCVCRTGWKCS	6	1	6
GCYWITKWCKQDSDCLRGRCG	6	1	6
GCWQLFVSCKQDSDCLAGCVCGPNGFCG	5	1	5
GCYWYRQWCKQDSDCLAGCG	5	1	5
GCPCILMRCKQDSDCLAGCVCHIAKMCG	5	1	5
GCWLIMWESPCKQDSDCLAGCVCHYNCG	4	1	4
GCPRILMRCKQDSDCLAGCVCEDPGECG	4	1	4
GCQYIGWECKAMELPCLAGCVCPHPDYCG	4	1	4
GCWLRPWICKQDSDCLAGCVCGPNGFCG	4	1	4
GCYWKMVWCKQDSDCLAGCG	4	1	4

GCPRILMRCKQDSDCLAGCVCKGWSMCG	4	1	4
GCLHGFHLCKQDSDCLAGCVCNQAKQCG	3	1	3
GCPRILMRCKQDSDCLAGCVCRPGWRCS	3	1	3
GCPRILMRCKQDSDCLAGCVCVPLFFCG	3	1	3
GCPRILMRCKQDSDCLAGCVCSAVIYCG	3	1	3
GCEAMWHDCKQDSDCLAGCVCGPNGFCG	3	1	3
GCWMVSGRCKQDSDCLAGCVCIQWLHCG	3	1	3
GCHWWNWSCKQDSDCLAGCVCSPNGFCG	3	1	3
GCPRILMRCKQDSDCLAGCVCPWTGRCG	3	1	3
GCPRILMRCKQDSDCLAGCVCFWFQVCG	3	1	3
GCSMVQYFCKQDSDCLAGCVCFIYFPCG	3	1	3
GCPRILMRCKQDSDCLAGCVCYPWYKCG	3	1	3
GCWWWSWVCKQDSDCLAGCVCRTVWSCG	3	1	3
GCWDKHERCKQDSDCLAGCVCRAGWKCS	3	1	3

Table S8. NGS results for lib 1 panning against human serum albumin. Top sequences are shown. S, number of the sequences shown in selection against the target protein; N, number of the sequences shown in selection against BSA + 1.

sequence	S	N	S/N
GKGKGAKCSLVSERIQFCCTGSGRSGKC	574	2	287
CKGKGAKCSGYTMKTCCTGSCRSGKC	218	1	218
CKGKGAKCSFVTANTQMCCTGSCRSDKC	68	1	68
CKGKGAKCSQLWPGMEFCCTGSCRSDKC	82	2	41
CKGKGAKCSEFFPGYAWCCTGSCRSDKC	30	1	30
CKGKGAKCSQLFDNFYWCCTGSCRSGKC	29	1	29
CKGKGAKCSPVTAHTQWCCTGSCRSGKC	26	1	26
CKGKGAKCSKLTDKINFCCTGSCRSDKC	23	1	23
CKGKGAKCSPLTENTLWCCTGSCRSGKC	20	1	20
CKGKGAKCSQVSQNVMYCCTGSCRSDKC	16	1	16
CKGKGAKCSKVWGDYFWCCTGSCRSGKC	30	2	15
CKGKGAKCSQLTERTWWCCTGSCRSGKC	15	1	15
CKGKGAKCSPLTKHTMYCCTGSCRSGKC	15	1	15
CKGKGAKCSEIMPKIYYCCTGSCRSDKC	14	1	14
CKGKGAKCSPFTANTMWCCTGSCRSGKC	12	1	12
CKGKGAKCSYLTERTQWCCTGSCRSGKC	12	1	12
CKGKGAKCSPVTTNTMWCCTGSCRSGKC	10	1	10
CKGKGAKCSRLTQHTMWCCTGSCRSGKC	10	1	10
CKGKGAKCSVLTTRTMYCCTDSCRSGKC	10	1	10
CKGKGAKCSQLTLNTMWCCTGSCRSGKC	9	1	9
CKGKGAKCSPLTARTQWCCTGSCRSGKC	8	1	8

CKGKGAKCSPLWPGMEWCCTGSCRSGKC	8	1	8
CKGKGAKCSYFTQNTLYCCTGSCRSGKC	14	2	7
CKGKGAKCSRFTQHTMWCCTGSCRSGKC	7	1	7
CKGKGAKCSMISPTIMYCCTGSCRSDKC	7	1	7
CKGKGAKCSLFTSGTYWCCTGSGRSGKC	7	1	7
CKGKGAKCSMMTEHTMWCCTGSCRSGKC	6	1	6
CKGKGAKCSKLTENVMYCCTGSCRSGKC	6	1	6
CKGKGAKCSLFMPGILWCCTGSCRSGKC	6	1	6

Table S9. NGS results for lib 2 panning against human serum albumin. Top sequences are shown. S, number of the sequences shown in selection against the target protein; N, number of the sequences shown in selection against BSA + 1.

sequence	S	N	S/N
VGECVRGRCKDTKCCSQFGYCGKGPKYCG	8	1	8
VGECVRGRCTKKECCSQFGYCGKGPKYCG	8	1	8
VGECVRGRCKSKECCSQFGYCGKGPKYCG	6	1	6
VGECVRGRCAKIKCCSQFGYCGKGPKYCG	6	1	6
VGECVRGRCKKSECCSQFGYCGKGPKYCG	6	1	6
VGECVRGRCKHNKCCSQFGYCGKGPKYCG	6	1	6
VGECVRGRCILKCCSQFGYCGKGPKYCG	8	2	4
VGECVRGRCSKHKCCSQFGYCGKGPKYCG	8	2	4
VGECVRGRCRVQCCSQFGYCGKGPKYCG	7	2	3.5
VGECVRGRCKKDNCCSQFGYCGKGPKYCG	8	3	2.7
VGECVRGRCEPECCSQFGYCGKGPKYCG	7	3	2.3
VGECVRGRCKHTKCCSQFGYCGKGPKYCG	7	3	2.3
VGECVRGRCEKSKCCSQFGYCGKGPKYCG	6	3	2
VGECVRGRCKHKECCSQFGYCGKGPKYCG	7	4	1.8
VGECVRGRCEKHKCCSQFGYCGKGPKYCG	7	4	1.8
VGECVRGRCKHEKCCSQFGYCGKGPKYCG	10	6	1.7
VGECVRGRCKHDKCCSQFGYCGKGPKYCG	9	6	1.5
VGECVRGRCKQKECCSQFGYCGKGPKYCG	6	4	1.5
VGECVRGRCKKTECCSQFGYCGKGPKYCG	6	4	1.5
VGECVRGRCKAKECCSQFGYCGKGPKYCG	6	4	1.5
VGECVRGRCDHKKCCSQFGYCGKGPKYCG	8	6	1.3
VGECVRGRCKVHKCCSQFGYCGKGPKYCG	9	7	1.3
VGECVRGRCKHQICCSQFGYCGKGPKYCG	5	4	1.3
VGECVRGRCKKECCSQFGYCGKGPKYCG	7	6	1.2
VGECVRGRCGKGPKYCG	7	6	1.2
VGECVRGRCKHKCCSQFGYCGKGPKYCG	18	16	1.1
VGECVRGRCHKHKCCSQFGYCGKGPKYCG	7	7	1

VGECVRGRCHKEKCCSQFGYCGKGPKYCG	7	7	1
VGECVRGRCSKKECCSQFGYCGKGPKYCG	6	6	1
VGECVRGRCDKKCCSQFGYCGKGPKYCG	7	8	0.9
VGECVRGRCKDKCCSQFGYCGKGPKYCG	6	8	0.8

Table S10. NGS results for lib E panning against human serum albumin. Top sequences are shown. S, number of the sequences shown in selection against the target protein; N, number of the sequences shown in selection against BSA + 1.

sequence	S	N	S/N
GCLYKKWHGNCKQDSDCLAGCVCHWYQSCG	467	2	234
GCLYKKWHAACKQDSDCLAGCVCYFSQSCG	69	1	69
GCYQMGAFCKQDSDCLAGCVCQMFGWCG	40	1	40
GCLYRKWHNQCKQDSDCLAGCVCHWYQSCG	21	1	21
GCKKHSMYWPCKQDSDCLAGCVCGPNGFCG	20	1	20
GCKKYGMWWPCKQDSDCLAGCVCGPNGFCG	12	1	12
GCFYRRWHQDCKQDSDCLAGCVCHWYQSCG	7	1	7
GCKKYWLIWPCKQDSDCLAGCVCGPNGFCG	7	1	7
GCKKYHMLWPCKQDSDCLAGCVCGPNGFCG	6	1	6
GCLYKKWHHMCKQDSDCLAGCVCHWYQSCG	5	1	5
GCHKYGLYWPCKQDSDCLAGCVCGPNGFCG	5	1	5
GCRKYDLIWPCKQDSDCLAGCVCGPNGFCG	5	1	5
GCPRILMRCKQDSDCLAGCVCWRAQQCG	5	1	5
GCWWRAHHCKQDSDCLAGCVCHFVQLCG	5	1	5
GCPRILMRCKQDSDCLAGCVCWSLSMCG	5	1	5
GCPRILMRCKQDSDCLAGCVCLFIMICG	4	1	4
GCPRILMRCKQDSDCLAGCVCHWYQSCG	11	3	3.7
GCVGSMGMCKQDSDCLAGCVCWDTGWYG	6	2	3
GCEWSGGCKQDSDCLAGCVCHWYQSCG	5	2	2.5
GCFPSYDFCKQDSDCLAGCVCPDPYMCG	5	2	2.5
GCRVFHSQCKQDSDCLAGCVCKLYMFCG	5	2	2.5
GCWITFHVSCKQDSDCLAGCVCVWYVSCG	5	2	2.5
GCVWVSTKCKQDSDCLAGCVCLAAICG	5	2	2.5
GCKLFGLQCKQDSDCLAGCVCGPNGFCG	4	2	2
GCRKTHRTKECKQDSDCLAGCVCPKKHCG	4	2	2
GCHWIAVSCKQDSDCLAGCVCWWRWRCG	9	7	1.3
GCDQAPTLCKQDSDCLAGCVCGPNGFCG	6	5	1.2
GCPKKMHKCKQDSDCLAGCVCKQSKHCG	6	6	1
GCWWMVSHCKQDSDYLAGCVCGPNGFCG	5	5	1
GCRFQANMCKQDSDCLAGCVCPFFWWCG	5	6	0.8

Table S11. Recombinant expression and folding analysis of Ig-DCPs and Hs-DCPs. Protein concentration was estimated by measuring the UV absorbance at 280 nm using Nanodrop. After removing the His(6)-SUMO tag using TEV protease, folding was analyzed using LC-MS. U.D.: undetectable.

name	sequence	Yield (mg/L)	3 S-S
Ig-AMP-1	VGECVRGRCSKISCCSQFGYCGKGPKYCG	5.4	U.D.
Ig-AMP-2	VGECVRGRCSAISCCSQFGYCGKGPKYCG	6.6	U.D.
Ig-AMP-3	VGECVRGRCSQISCCSQFGYCGKGPKYCG	7.0	yes
Ig-AMP-4	VGECVRGRCSSISCCSQFGYCGKGPKYCG	9.5	yes
Ig-CPI-1	HADPICNKPCMLFAPCSGAWFCQACWNSARTCGPYVG	7.0	yes
Ig-CON-1	ECKGKGAKCSSLSYHEIQSPCCTGSCRSGKC	18.9	yes
Ig-CON-2	ECKGKGAKCSFIQWTPSYQDPQCCTGSCRSGKC	7.0	yes
Ig-CON-3	ECKGKGAKCSSVSYAEDWGHQGCCTGSCRSGKC	7.2	yes
Ig-CON-4	ECKGKGAKCSSMSVLDTHYDCCTGSCRSGKC	7.1	yes
Ig-CON-5	ECKGKGAKCSSISIHDAHCCTGSCRSGKC	20.2	yes
Ig-CON-6	ECKGKGAKCSYMIYEEPIQHCCTGSCRSGKC	8.4	yes
Ig-CON-7	ECKGKGAKCSSISSYHRDYAWECCTGSCRSGKC	6.7	yes
Ig-EET-1	GCQAHYWYRVWFCKQDSDCLAGCVCGPNGFCG	2.6	yes
Ig-EET-2	GCGFWYMQWFCKQDSDCLAGCVCDPMGNCG	4.6	yes
Ig-EET-3	GCGFWYHKWFCKQDSDCLAGCVCSKTGCG	5.2	yes
Ig-EET-4	GCGYWYMQWFCKQDSDCLAGCVCRPKPECG	2.0	U.D.
Hs-Chr-1	VSCTTSKECWSVCQRLHNTSKGRCDEICGCRW	3.6	yes (weak)
Hs-Chr-2	VSCTTSKECWSVCQRLHNTSRGRCEEICGCSN	12.9	yes
Hs-Chr-3	VSCTTSKECWSVCQRLHNTSYGTGCMRNTCYCTP	5.2	U.D.
Hs-EET-1	GCLYRKWHNQCKQDSDCLAGCVCHWYQSCG	3.0	U.D.
Hs-EET-2	GCLYKKWHAACKQDSDCLAGCVCYFSQSCG	3.7	yes
Hs-EET-n2	GCYQMGAFCKQDSDCLAGCVCQMFGWCG	4.8	yes
Hs-CON-n1	ECKGKGAKCSFVTANTQMCCTGSCRSDKC	11.7	yes
Hs-CON-n2	ECKGKGAKCSQLWPGMEFCCTGSCRSDKC	5.3	yes

Table S12. Ig-DCPs and Hs-DCPs bind to their targets with sub-micromolar affinity. DCP variants were selected and produced through chemical synthesis and folding. Final products were quality controlled using LC-MS. The IC50s of the DCPs were measured using phage competition (synthetic DCPs competing with their own phage for binding to the target) ELISA. The K_D valuess of the potential DCP binders were measured using SPR. Values were averaged from at least three independent runs. N.A.: no detectable binding. *: estimated value due to possible non-specific binding.

name	sequence	IC_{50} (μM)	K_D (μM)
WT AMP	VGECVRGRCPSGMCCSQFGYCGKGPKYCG		N.A.
Ig-AMP-1	VGECVRGRCSKISCCSQFGYCGKGPKYCG	0.055	0.243
Ig-AMP-2	VGECVRGRCSAISCCSQFGYCGKGPKYCG	0.041	aggregation
Ig-AMP-3	VGECVRGRCSQISCCSQFGYCGKGPKYCG	0.062	0.353
Ig-AMP-4	VGECVRGRCSSISCCSQFGYCGKGPKYCG	0.151	> 1
WT CON	CKGKGAKCSRLMYDCCTGSCRSGKC		> 20
Ig-CON-5	ECKGKGAKCSSISIHDAHCCTGSCRSGKC	0.02	>0.03*
Ig-CON-7	ECKGKGAKCSSISSYHRDYAWECCTGSCRSGKC	0.107	0.161
WT EETI	GCPRILMRCKQDSDCLAGCVCGPNGFCG		N.A.
Ig-EET-1	GCQAHYWYRVWFCKQDSDCLAGCVCGPNGFCG	0.447	0.228
Ig-EET-4	GCGYWYMQWFCKQDSDCLAGCVCRPKPECG	0.823	0.948
WT Chr	VSCTTSKECWSVCQRLHNTSKGGCQGSFCTCGP		N.A.
Hs-Chr-1	VSCTTSKECWSVCQRLHNTSKGRCDEICGCRW	1.09	5.33
Hs-Chr-2	VSCTTSKECWSVCQRLHNTSRGRCEEICGCSN	3.30	
Hs-Chr-3	VSCTTSKECWSVCQRLHNTSYGTGCMRNTCYCTP	0.09	0.44

Table S13. Sequence identity scores of human, rabbit, rat and mouse serum albumin or IgG Fc. Sequence alignment was performed using ClustalW.

IgG1 Fc	Rabbit	Rat	Mouse	Albumin	Rabbit	Rat	Mouse
Human	72%	65%	64%	Human	71%	72%	71%

Table S14. NGS results for lib 1, 2, E (mixed together) panning against VEGF-A. Top sequences are shown. S, number of the sequences shown in selection against the target protein; N, number of the sequences shown in selection against BSA + 1.

sequence	S	N	S/N
GCTTPWVPHHIMCKQDSDCLAGCVCGPNGFCG	410	3	137
CKGKGAKCSAVWATDQEYHVICCTGSCRSGKC	330	4	83
GCDAKKYAYGCKQDSDCLAGCVCRATSSCG	70	1	70
VGECSQPWFINMCNMVDCCSQFGYCGKGPKYCG	304	5	61
CKGKGAKCSGVWQTPGSTHVVCCTGSCRSGKC	122	3	41
GCQTPWFPHQILCKQDSDCLAGCVCGPNGFCG	38	1	38
VGECQSAWFISMCNMVNCCSQFGYCGKGPKYCG	25	1	25
GCAGKSCEMKTYENCDAGCFCRILQLKDCV	22	1	22
GCMTPWFPFTINCKQDSDCLAGCVCGPNGFCG	22	1	22
GCTTPWSPFLVMCKQDSDCLAGCVCGPNGFCG	22	1	22
GCSWYWSMMRWCKQDSDCLAGCVCAEAHPCG	41	2	21
CKGKGAKCSDTWVVQNHAYTICCTGSCRSGKC	38	2	19
GCAGKSCGQHMETECDAGCFCRMFRFKDCV	17	1	17

GCQTPWPPYDILCKQDSDCLAGCVCGPNGFCG	17	1	17
VGECNSPWVISVCQVIDCCSQFGYCGKGPKYCG	76	5	15
GCKVEMAAFGCKQDSDCLAGCVCAFYDSCG	15	1	15
VGECEKEWLVSMCQWVDCCSQFGYCGKGPKYCG	14	1	14
GCDMTMPMWGCKQDSDCLAGCVCRYYESCG	14	1	14
CKGKGAKCSATWFTAGDGHVICCTGSCRSGKC	66	5	13
VGECMPAWQRSYCKWVDCCSQFGYCGKGPKYCG	13	1	13
GCNHSRFKQYCKQDSDCLAGCVCGPNGFCG	13	1	13
GCAGKSCKKWSSVHCDAGCFCQIGQQVVYIPGCV	111	9	12
GCKISAFRQYCKQDSDCLAGCVCGPNGFCG	12	1	12
GCQPDKMHWGCKQDSDCLAGCVCQHYNSCG	1447	174	8
GCAGKSCIKRNMDNCDAGCFCKIFQFKDCV	66	10	7
GCAGKSCVTEYQSNCDAGCFCTLLGFRDCV	11	3	4
GCAGKSCNILGSDPCDAGCFCTKYIYWMDHHKCV	10	3	3
CKGKGAKCSGSWQIDDTHYVICCTGSCRSGKC	40	13	3
GCAGKSCITPVFDECDAGCFCKIFGWSDCV	15	6	3
GCAGKSCTGYTQIYCDAGCFCVVFESIVHVPGCV	11	6	2

Table S15. NGS results for lib 1, 2, E (mixed together) panning against human Ly6E (Rat IgG2b Fc fusion, A) and PDGF bb (B). Top sequences are shown. S, number of the sequences shown in selection against the target protein; N, number of the sequences shown in selection against BSA + 1.

A

sequence	S	N	S/N
GCNMYSGPWRYFCKQDSDCLAGCVCGPNGFCG	1721	4	430
GCRMYYGTWRYTCKQDSDCLAGCVCGPNGFCG	1070	7	153
GCLMYIQTHQYPCKQDSDCLAGCVCGPNGFCG	99	1	99
GCAIYHMGWMFQCKQDSDCLAGCVCGPNGFCG	178	2	89
GCKMYGLGHWYGCKQDSDCLAGCVCGPNGFCG	64	1	64
GCRLYSMGWFYSCKQDSDCLAGCVCGPNGFCG	48	1	48
GCSQFRSPMMWQCKQDSDCLAGYVCGPNGFCG	40	1	40
GCKMYAMGWLYTCKQDSDCLAGCVCGPNGFCG	37	1	37
GCQIYQSQWSYLCKQDSDCLAGCVCGPNGFCG	103	3	34
GCPEIMGPMTWHCKQDSDCLAGYVCGPNGFCG	34	1	34
GCKLYTGGWMYLCKQDSDCLAGCVCGPNGFCG	28	1	28
GCKHFWWVCKQDSDCLAGCVCGPNGFCG	28	1	28
GCTMYQQGWSYSCKQDSDCLAGCVCGPNGFCG	24	1	24
GCKHFWWVCKQDSDCHAVCGPNGFCG	20	1	20
GCYDWWSPRTWTCKQDSDCLAGYVCGPNGFCG	19	1	19
GCWDGMILIGYWCKQDSDCLAGCVCDWEPMCS	19	1	19
GCRIYQGEYSYPCKQNSDCLAGCVCGPNGFCG	17	1	17

GCQMFQSPHTWDCKQDSDCLAGYVCGPNGFCG	17	1	17
GCGMYHMDWFYMCKQDSDCLAGCVCGPNGFCG	14	1	14
GCDHFWVVCKQDSDCLAGCVCEVMSACG	14	1	14
GCKYWWMQIFSMCKQDSDCLAGCVCWLYGDCG	12	1	12
GCHMYMGAWKFPCKQDSDCLAGCVCGPNGFCG	12	1	12
GCSFYTMGWSYQCKQDSDCLAGCVCGPNGFCG	11	1	11
GCQMYSKHWLYPCKQDSDCLAGCVCGPNGFCG	11	1	11

B

sequence	S	N	S/N
GCMDPDWVCKQDSDCLAGCVCWQPEGCG	95	5	19
GCHSWLQEMFGFCKQDSDCLAGCVCEISETCG	33	1	33
GCAFEGESCNVQFYPCCVSMKLMCIPGNPDGTCYYL	13	1	13
GCFDDNPFIACKQDSDCLAGCVCGPNGFCG	11	1	11
GCWAPSSGFDFTCKQDSDCLAGCVCSMMNGCG	8	1	8
GCFDDNPFIACKQDSDCLAGCVCWYDTWCG	7	1	7

Table S16. Discovery of DCPs binding to human VEGF-A or PDGF bb. Potential DCPs were selected based on NGS analysis and sequence variation. They were synthesized, folded, and purified. Final products were quality controlled using LC-MS. The K_D values against VEGF-A (for V-DCPs) or PDGF bb (for P-DCPs were measured using SPR. Values were averaged from at least three independent runs. Estimated K_D values are shown due to possible non-specific binding.

name	sequence	S	S/N	K_D (μM)
V-CON-1	CKGKGAKCSAVWATDQEYHVICCTGSCRSGKC	330	83	>4.6
V-MCH-1	GCAGKSCKKWSSVHCDAGCFCQIGQQVVYIPGCV	111	12	>50
V-AMP-1	VGECSQPWFINMCNMVDCCSQFGYCGKGPKYCG	304	61	>3.6
V-EET-1	GCTTPWVPHHIMCKQDSDCLAGCVCGPNGFCG	410	137	>20
V-EET-2	GCQTPWFPHQILCKQDSDCLAGCVCGPNGFCG	38	38	>30
V-EET-3	GCMTPWFPFTINCKQDSDCLAGCVCGPNGFCG	22	22	>1.5
P-EET-2	GCSHWLQEMFGFCKQDSDCLAGCVCEISETCG	33	33	>20
P-EET-6	GCFDDNPFIACKQDSDCLAGCVCWYDTWCG	11	11	>6.7
P-AST-3	GCAFEGESCNVQFYPCCVSMKLMCIPGNPDGTCYYL	13	13	>50

Table S17. Discovery of DCPs binding to rat Fc-Ly6E. Potential DCPs were selected based on NGS analysis and sequence variation. They were synthesized, folded, and purified. Final products were quality controlled using LC-MS. The K_D values against rat Fc-Ly6E (highlighted in red), rat Fc (highlighted in blue) or human Fc (highlighted in green) were measured using SPR. Values were averaged from at least three independent runs. N.A.: no binding. Estimated K_D values are shown due to possible non-specific binding.

name	sequence	S	S/N	K_D (μM)		
L-EET-5	GCNMYSGPWRYFCKQDSDCLAGCVCGPNGFCG	1721	430	>20	>20	
L-EET-7	GCAIYHMGWMFQCKQDSDCLAGCVCGPNGFCG	178	89	0.022	0.065	N.A.
L-EET-8	GCQIYQSQWSYLCKQDSDCLAGCVCGPNGFCG	103	34	>20	>20	N.A.
